# Genome-wide profiling of adenine base editor specificity by EndoV-seq

**DOI:** 10.1038/s41467-018-07988-z

**Published:** 2019-01-08

**Authors:** Puping Liang, Xiaowei Xie, Shengyao Zhi, Hongwei Sun, Xiya Zhang, Yu Chen, Yuxi Chen, Yuanyan Xiong, Wenbin Ma, Dan Liu, Junjiu Huang, Zhou Songyang

**Affiliations:** 1 The First Affiliated Hospital, Sun Yat-sen University; MOE Key Laboratory of Gene Function and Regulation, Guangzhou Key Laboratory of Healthy Aging Research, SYSU-BCM Joint Research Center, School of Life Sciences, Sun Yat-sen University, 510275 Guangzhou, China; 2 Key Laboratory of Reproductive Medicine of Guangdong Province, School of Life Sciences and the the First Affiliated Hospital, Sun Yat-sen University, 510275 Guangzhou, China; 3 State Key Laboratory of Experimental Hematology, Institute of Hematology and Blood Diseases Hospital, Chinese Academy of Medical Sciences and Peking Union Medical College, 3000000 Tianjin, China; 4 Verna and Marrs Mclean Department of Biochemistry and Molecular Biology, Baylor College of Medicine, One Baylor Plaza, 77030 Houston, TX USA; 5 State Key Laboratory of Ophthalmology, Zhongshan Ophthalmic Center, Sun Yat-sen University, Guangzhou, 510060 China; 6 Key Laboratory of Reproductive Medicine of Guangdong Province, the Third Affiliated Hospital of Guangzhou Medical University, 510150 Guangzhou, China

## Abstract

The adenine base editor (ABE), capable of catalyzing A•T to G•C conversions, is an important gene editing toolbox. Here, we systematically evaluate genome-wide off-target deamination by ABEs using the EndoV-seq platform we developed. EndoV-seq utilizes Endonuclease V to nick the inosine-containing DNA strand of genomic DNA deaminated by ABE in vitro. The treated DNA is then whole-genome sequenced to identify off-target sites. Of the eight gRNAs we tested with ABE, 2–19 (with an average of 8.0) off-target sites are found, significantly fewer than those found for canonical Cas9 nuclease (7–320, 160.7 on average). In vivo off-target deamination is further validated through target site deep sequencing. Moreover, we demonstrated that six different ABE-gRNA complexes could be examined in a single EndoV-seq assay. Our study presents the first detection method to evaluate genome-wide off-target effects of ABE, and reveals possible similarities and differences between ABE and canonical Cas9 nuclease.

## Abstract (web summary)

Adenine base editors are an important contribution to the genome editing toolbox. Here the authors present EndoV-seq, an endonuclease-based assay for evaluating genomewide off-target effects of base editing.

## Introduction

The recently developed targeted base replacement strategy using deaminases holds great promise for treating human diseases caused by pathogenic single nucleotide polymorphisms (SNPs). These RNA-directed programmable base editors can carry out single base pair conversions without inducing double strand breaks (DSBs)^1^. Cytosine base editors (CBEs) such as base editor 3 (BE3), which catalyze C•G to T•A base pair conversion^1^, have been successfully used to edit target bases in zebrafish, mouse, and human^2–7^. Base A deamination results in I (inosine) or X (xanthosine), where base I can pair with C and be replicated as G. Adenosine base editors (ABEs) rely on the tRNA-specific adenosine deaminase (TadA) from *Escherichia coli* to convert A to I on the non-complementary strand, and Cas9 nickase (nCas9) to nick the complementary strand of the target site, thus achieving A•T to G•C pair conversions^8^. We and others have shown efficient adenine base editing by ABEs in human cells, mouse embryos, and rat embryos^8–13^.

Approximately 48% of known pathogenic SNPs may be corrected by A•T-to-G•C conversion, and >20% of these may be targetable with SpCas9-based ABEs, indicating tremendous potential for SpCas9-based ABEs in gene therapy^8,14^. The advent of xCas9, with its broadened PAM sequence range (5′-NGN, 5′-GAA, 5′-GAT, and 5′-CAA), promises even wider utility of ABE, as more pathogenic G•C-to-A•T SNPs may be corrected by xCas9-ABE^14^. However, critical questions regarding the specificity and off-target effects of ABEs remain and must be addressed before any possible clinical translation^15^.

Digenome-seq has been developed to study genome-wide off-target effects of genome editing tools, where sequencing reads of in vitro processed genomic DNA are mapped to reference genomes with chromosomal sites scored based on DNA reads with identical 5′ or 3′ ends^16,17^. The method has been successfully used to evaluate genome-wide off-target effects of Cas9, Cpf1, and BE3^16–22^. Because the enzymes used in these previous reports cannot cleave ABE-modified DNA^22^, new assays for assessing ABE activities are thus necessary.

In this study, we describe a method (EndoV-seq) to investigate ABE specificity genome-wide, where in vitro deaminated genomic DNA is digested with Endonuclease V (EndoV) before being subjected to whole-genome sequencing (WGS). EndoV-seq enables us to evaluate both on-target and off-target deamination by ABE. We further validate the results through target site deep sequencing to confirm the in vivo specificity of ABE. In addition, our findings show that EndoV-seq is amenable to multiplexing and offers clues to how ABE specificity may be improved.

## Results

### Using EndoV-seq to detect on-target deamination by ABE

The Cas9 nuclease can cleave genomic sites with mismatches to the gRNA^23^. We therefore investigated the effects of mismatch on the A•T-to-G•C conversion efficiency of ABE at target sites. A series of 20-nt mismatched gRNAs (with 1–3 base changes) targeting three endogenous sites (*HEK293-2*, *VEGFA3*, and *HBG2*) were generated and co-expressed in 293T cells with the ABE variant ABE7.10 or Cas9 (Supplementary Figure 1). Similar to the canonical Cas9 nuclease, ABE appeared to tolerate mismatches between the gRNA and its target sites, especially for 1 or 2-nt mismatches at positions distal to the PAM (Supplementary Figure 1), highlighting the need of developing a genome-wide method to detect ABE off-target effects.

ABE specificity and off-target assessment requires an endonuclease that can recognize base I, the deaminated product of base A. EndoV (also known as deoxyinosine 3′ endonuclease) from *Thermotoga maritima* is a repair enzyme that recognizes deoxyinosines and hydrolyzes the second phosphodiester bond 3′ of the inosine base, resulting in nicked DNA^24^. We reasoned that EndoV digestion of ABE-treated DNA would generate DSBs, which should enable detection of ABE-mediated base conversion^8^. To test the feasibility of this idea, we investigated the possibility of inducing DSBs at ABE target sites following treatment with the gRNA-ABE7.10 complex and EndoV^8^.

**Fig. 1:**
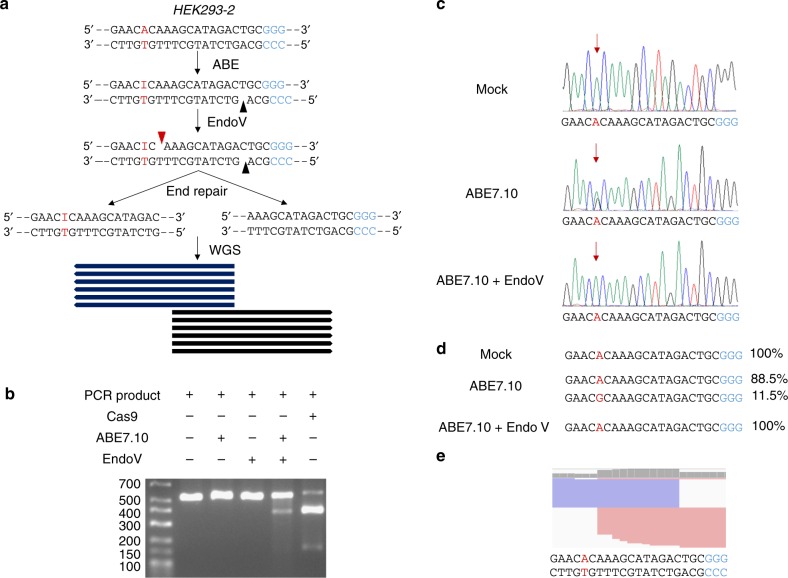
Using EndoV-seq to evaluate on-target deamination by ABE. **a** A flow chart for assessing in vitro ABE off-target effects by EndoV-seq is shown, using sequences from the *HEK293-2* site as an example. Genomic DNA is first incubated with recombinant ABE7.10 and the appropriate gRNA and then digested with EndoV, thereby allowing the DNA to be nicked by both nCas9 nickase (black triangle) and EndoV (red triangle, one residue downstream of base I). The cleaved DNA is subsequently fragmented and end repaired for whole-genome sequencing (WGS) with ~30–40 fold coverage. **b** Genomic DNA of 293T cells was used to PCR amplify regions spanning the *HEK293-2* site. The PCR products (100 ng) were incubated with ABE7.10 (300 nM) and *HEK293-2* gRNA (900 nM) for 3 h before EndoV (1U) incubation (30 min). The treated products were resolved by agarose gel electrophoresis. Recombinant Cas9 was used as a positive control for DNA cleavage. Molecular weight marker size is in base pairs. Source data are provided as a Source Data file. **c** Sanger sequencing chromatograms of PCR products amplified from the *HEK293-2* gRNA target site using genomic DNA (10 µg) treated with ABE7.10 (300 nM, 8 h) ± EndoV (8U, 3 h). Mock treated genomic DNA served as a control. PAM, blue. Target base A, red and highlighted with red arrow. Peaks on the chromatograph, green for A, red for T, blue for C, and black for G. **d** PCR products from **c** were deep sequenced. The frequency of each allele is shown on the right. PAM, blue. Target base A, red. **e** Alignment of whole-genome sequencing reads of the *HEK293-2* gRNA target region as visualized by the Integrative Genomics Viewer (IGV). Target base A, red. PAM, blue

First, we PCR amplified the region spanning the well-characterized *HEK293-2* site and incubated the PCR products with recombinant ABE7.10 protein (Supplementary Figure 2) and the corresponding gRNA, for A-to-I conversion and nCas9 nicking^22^. The treated PCR products were subsequently digested with EndoV to generate DSBs (Fig. 1a). As predicted, the PCR products were indeed cleaved into smaller fragments after both ABE7.10 and EndoV treatment (Fig. 1b). Similar in vitro cleavage by ABE7.10 and EndoV was observed when we analyzed a 19-nt gRNA targeting exon 66 in the mouse *Dmd* gene locus (Supplementary Figure 3a and b)^12^.

For further testing, genomic DNA (from human or mouse) was first deaminated by recombinant ABE7.10 and the corresponding gRNA (human *HEK293-2* or mouse *Dmd*). Target base deamination was then confirmed through Sanger and deep sequencing (Fig. 1c and d and Supplementary Figure 3c and d). The A-to-I conversion rate at the *Dmd* locus appeared higher than that of *HEK293-2* (29.9% vs. 11.5%), suggesting possible sequence-dependence of ABE activity. EndoV digestion completely depleted the base G peak, indicating highly efficient cleavage of the deaminated strand by EndoV (Fig. 1c, d and Supplementary Figure 3c, d). The ABE/EndoV-treated genomic DNA was then whole genome sequenced (WGS, 30–40-fold coverage) (Fig. 1a and Supplementary Figure 3a). WGS results showed many DNA reads with identical 5′ or 3′ ends at the on-target sites of *HEK293-2* and *Dmd* (Fig. 1e and Supplementary Figure 3e). We hence named this method EndoV-seq, which appeared to effectively detect ABE on-target sites in human and mouse genomes.

### EndoV-seq profiles genome-wide off-target effects of ABE

Next, we selected another six-well-characterized gRNAs that target seven human genes—*VEGFA3*, *RNF2*, *HBB-28 (A* > *G)* mutant allele, *EMX1*, *FANCF*, *HBG1*, and *HBG2*^7,8,18,25^—to evaluate their specificity by EndoV-seq. Of these, the *HBB-28 (A* *>* *G)* mutant allele is targeted by the *HBB-28 (T* > *C)* gRNA (18-nt)^7^, while the *HBG* gRNA can target both *HBG1* and *HBG2*^8^. Except for *HBB*, qPCR analysis of EndoV-treated genomic DNA showed varying degrees of reduction in the copy number of intact target genes (Supplementary Figure 4), indicating successful cleavage of on-target sites by ABE7.10 and EndoV. Because the *HBB-28 (T* > *C)* gRNA recognizes the *HBB-28 (A* > *G)* mutant allele, it may not efficiently target the wild-type *HBB* allele in HEK-293T genomic DNA.

**Fig. 2:**
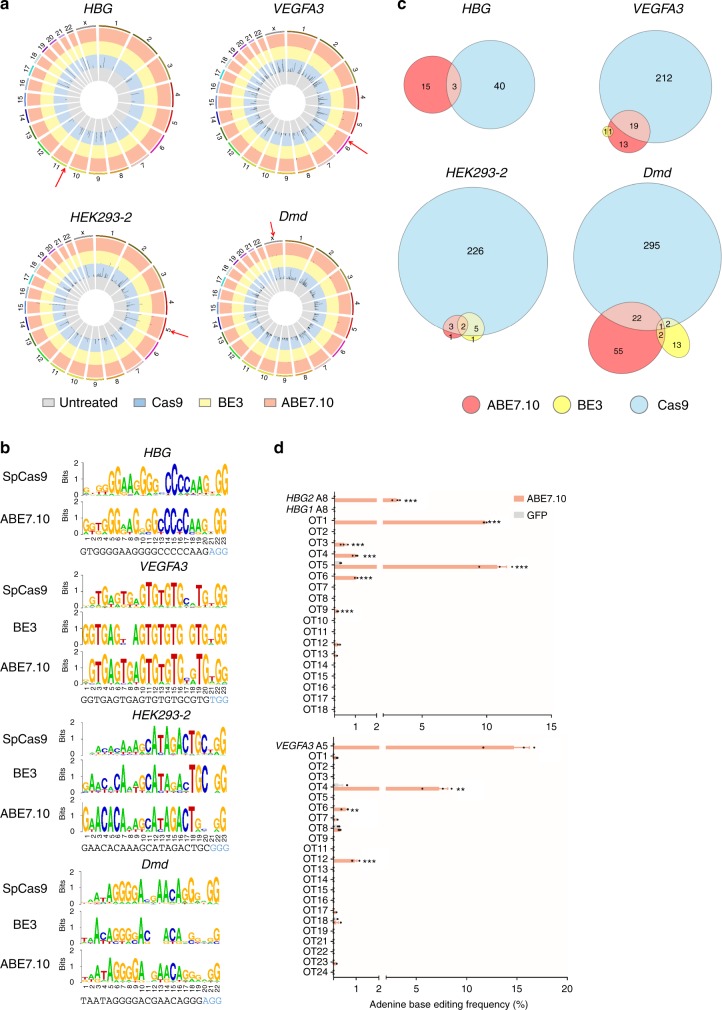
Using EndoV-seq to profile genome-wide off-target deamination by ABE. **a** Genome-wide cleavage scores (cutoff score of >2.5) of genomic DNA treated with Cas9 (blue), BE3 (yellow), or ABE7.10 (coral) using human *HBG*, *VEGFA3*, *HEK293-2*, or mouse *Dmd* gRNAs. Untreated genomic DNA (gray) served as controls. Red arrows, on-target sites. **b** Sequence logos of EndoV-captured (ABE7.10) and Digenome-captured (Cas9 and BE3) off-target (with scores of >2.5) and on-target sites of the listed gRNAs. Target sequences are shown with PAM in blue. Note: The length of *Dmd* gRNA is 19-nt. **c** Venn diagrams that compare Digenome-captured sites for Cas9 and BE3 with EndoV-seq captured sites of ABE7.10 (score of >0.1 for ABE7.10 and BE3, score of >2.5 for Cas9) are shown for the target sites listed. **d** HEK-293T cells were co-transfected with vectors encoding ABE7.10 together with *HBG* gRNA (that targets both *HBG1* and *HBG2*) and *VEGFA3* gRNA. At 48 h after transfection, genomic DNA was extracted for PCR amplification and deep sequencing. GFP-transfected cells were used as controls. Error bars represent SEM (*n* = 3). Statistical significance was calculated using a two-tailed unpaired *t*-test (***p < 0.001). OT, off-target. OT10 of *VEGFA3* failed to be amplified by PCR. Source data are provided as a Source Data file

In order to capture the genome-wide off-target sites of ABE, we further parsed EndoV-seq results to score each genomic position using a program reported by Kim et al. ^22^. Since the specificity of base editors is dictated by both the Cas9 and the deaminase, we sought to compare ABE7.10 with Cas9, as well as BE3 (the latter two examined via Digenome-seq) (Supplementary Figure 2). For ABE7.10, we found 2–19 (8.0 on average) potential off-target sites for the tested gRNAs, much lower than those of canonical Cas9 (7-320, 160.7 on average) (with cutoff cleavage score > 2.5) (Fig. 2a, Supplementary Figure 5a, b, and Supplementary Tables 1–8 and 27). In agreement with previous findings, fewer off-target sites were found with BE3 than Cas9 (Fig. 2a, Supplementary Figure 5a, and Supplementary Tables 9–27)^22^. Weblogo also revealed higher sensitivity of ABE7.10 and BE3 to sequence mismatches in PAM-distal regions (Fig. 2b and Supplementary Figure 5c, d), suggesting that base editors may be more reliant on specific gRNA sequences than canonical Cas9 nuclease.

To rule out the possibility that the observed difference in potential off-target sites for ABE7.10 and Cas9 (8.0 vs. 160.7) was caused by differing sensitivities of EndoV-seq vs. Digenome-seq, we diluted genomic DNA treated with ABE7.10*-HEK293-2* gRNA or Cas9*-HEK293-2* gRNA with untreated DNA before further analysis. At 2.5-fold dilution, both EndoV-seq and Digenome-seq could still robustly detect respective editing by ABE and Cas9 (score > 0.1) (Supplementary Figure 6). The ability of both methods to detect editing dropped precipitously at five-fold dilution (score < 0.1), and neither was able to detect any editing upon further dilution, suggesting comparable sensitivities of ABE EndoV-seq and Cas9 Digenome-seq.

When we lowered the cutoff cleavage score to 0.1, as previously reported for Digenome-seq analysis of BE3∆UGI^22^, more off-target sites were identified for ABE7.10 (5–80, 24.1 on average) and BE3 (0–31, 11.3 on average) (Supplementary Figure 7a and b, Supplementary Tables 1–14 and 27). Closer examination revealed overlapping cleavage sites of ABE7.10 and BE3 with Cas9-cleaved sites, especially for sites with scores above 2.5 (Fig. 2c, Supplementary Figure 7c, d, and Supplementary Table 28), implying that the gRNA sequence is a major determinant of specificity. Although Cas9 Digenome-seq captured many more off-target sites than either ABE or BE3 (Fig. 2c, Supplementary Figure 7c and d, and Supplementary Table 27), there were also unique off-target sites for both ABE7.10 and BE3 that were not found for Cas9, consistent with the notion that base editors have unique off-target spectra compared with Cas9.

To validate in vivo off-target effects at sites captured by EndoV-seq, we co-expressed ABE7.10 and various gRNAs in HEK-293T cells and carried out target site deep sequencing. Of the eight gRNAs tested, in vivo A-to-G conversion was observed at six out of nine on-target sites, indicating efficient editing of these sites (Fig. 2d and Supplementary Figure 8). On-target deamination was found at the *HBG2* site but not *HBG1*, which may be a result of epigenetic modifications and/or chromatin structures that blocked ABE access^26–28^, a possibility that may also explain the lack of editing at the *EMX1* target site. Again, the wild-type *HBB* locus in HEK-293T cells was not edited by the *HBB-28 (T* *>* *C)* mutant gRNA, consistent with our in vitro findings (Supplementary Figure 5a).

A-to-G conversion was found in these transfected cells at nine off-target sites detected by EndoV-seq for *HBG* (six) and *VEGFA3* (three) (Fig. 2d), underlining the effectiveness of using EndoV-seq to detect ABE off-target sites. Interestingly, neither Cas9 Digenome-seq nor BE3 Digenome-seq detected six out of the nine validated off-target sites, demonstrating that EndoV-seq is a more specific method for ABE off-target detection (Supplementary Tables 1 and 2). Furthermore, we were able to confirm the EndoV-seq detected off-target site (HBG-OT9) that was edited at the frequency of 0.13% in vivo (Fig. 2d), further attesting to the sensitivity of EndoV-seq. We failed to identify off-target deamination by ABE for the remaining six gRNAs in vivo (Supplementary Figure 8). To ensure that we did not miss off-target sites due to small sample size, we examined an additional 100 off-target sites for the same gRNAs that contain base A within the deamination window and had been identified by Digenome-seq (53 for Cas9 and 47 for BE3) but not by ABE EndoV-seq (Supplementary Tables 9–20). Again, no apparent A-to-G conversion could be found at any of these sites (Supplementary Figure 9), indicating that ABE off-targets may be rare. Collectively, our findings suggest that Digenome-seq may be less suitable for probing ABE specificity and support EndoV-seq as an effective and sensitive method to detect genome-wide off-target effects of ABE.

### Genome-wide off-target profiles by multiplex EndoV-seq

**Fig. 3:**
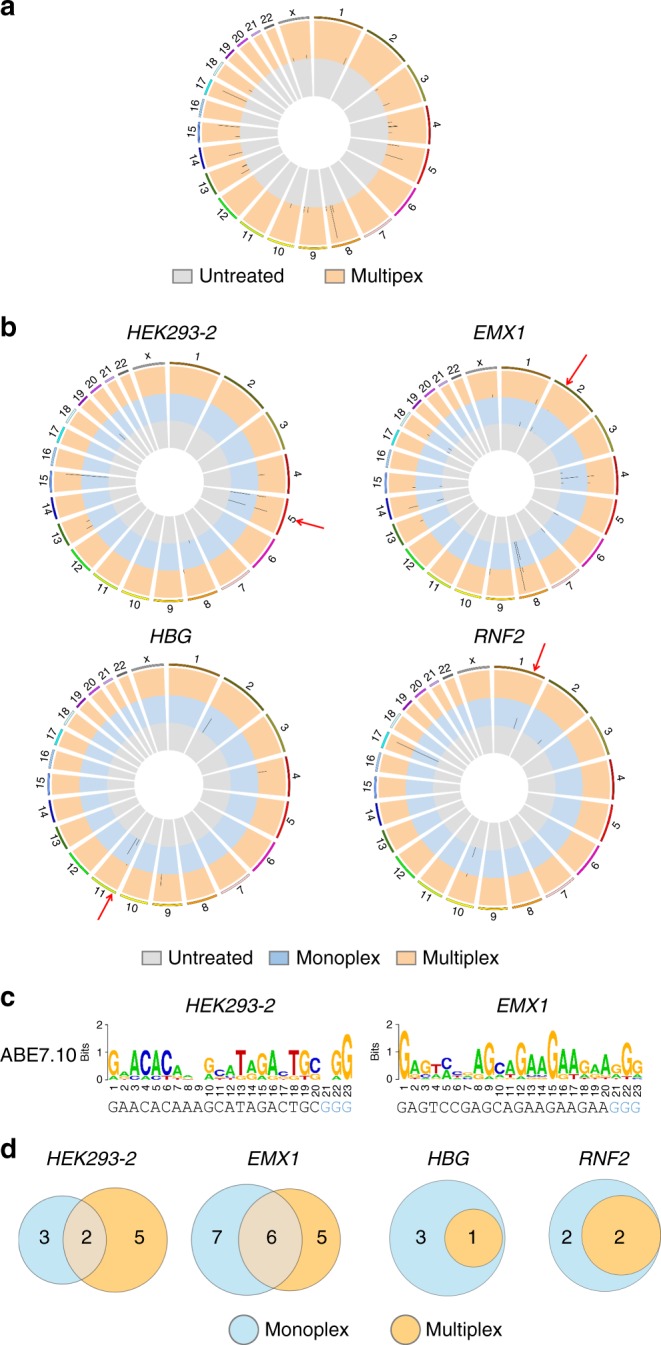
Using multiplex EndoV-seq to profile off-target effects of ABE. **a** Human genomic DNA (10 μg) was treated with a mixture of 300 nM ABE7.10 and six gRNAs (200 nM each) (*HEK293-2*, *EMX1*, *FANCF*, *HBB-28 (T* *>* *C)*, *RNF2* and *HBG*) for 8 h and then with EndoV (8 U) for 3 h. The treated gnomic DNA was subsequently sequenced with ~30–40-fold coverage and genome-wide cleavage scores calculated. Sites with cleavage score of >2.5 are plotted here. Orange, genomic DNA treated with ABE7.10 and multiplex gRNAs. Gray, untreated genomic DNA. **b** Genome-wide cleavage scores (cutoff > 2.5) of untreated (gray), monoplex EndoV-seq (blue), and multiplex EndoV-seq (orange) for the indicated gRNAs are plotted. Red arrows, on-target sites. **c** Sequence logos (by WebLogo) of multiplex EndoV-seq captured off-target (DNA cleavage scores of >2.5) and on-target sites. **d** A comparison of monoplex vs. multiplex EndoV-seq captured off-target sites (DNA cleavage scores of >2.5, on-target sites not shown)

Multiplex Digenome-seq was recently used to capture potential off-target sites of Cas9 using 11 gRNAs^18^. While multiplex EndoV-seq would be considerably more cost-effective, whether it can reliably and accurately detect off-target sites of ABEs needs to be determined. To this end, we carried out multiplex EndoV-seq using six gRNAs (*HEK293-2*, *EMX1*, *HBG*, *RNF2*, *FANCF*, and *HBB-28 (T* *>* *C)*). Genomic DNA was treated with a mixture of ABE7.10 protein and the six gRNAs and further digested with EndoV. Cleavage of target sites was confirmed by qPCR (Supplementary Figure 10a), and the treated DNA was whole-genome sequenced (30–40-fold coverage) with each genomic position scored as previously described^22^. With scores of >2.5, multiplex EndoV-seq detected 25 sites compared to 32 in all from six monoplex EndoV-seq assays (Fig. 3a and Supplementary Tables 2, 3–7, and 29). Lowering the score to >0.1 slightly increased the number of sites found over monoplex assays (103 vs. 85) (Supplementary Tables 1, 3–7, and 29). Next, we used Site Allocator (Supplementary Software 1), developed in-house and based on the program for Cas9 multiplex Digenome-seq^18^, to estimate the similarity (edit distance) between the six gRNA target sites (or edit distance), which ranged from 0 (each site against itself) to 17.0 (e.g., *HBG*-*FANCF*) with a mean edit distance of 13.1 (Supplementary Table 30). We then used Site Allocator to calculate the edit distance between the 103 sites and each of the six gRNA target sites. Assuming each site was captured by the gRNA with the smallest edit distance^18^, the 103 sites could thus be divided into six groups (Fig. 3b, Supplementary Figure 10b, and Supplementary Table 31). Further analysis revealed sequence motifs of the captured sites that matched those identified through monoplexed EndoV-seq (Figs. 2b, 3c and Supplementary Figure 5d), and substantial overlap (except for *FANCF*) between sites captured by multiplex and monoplex EndoV-seq (Fig. 3d and Supplementary Figure 10c). In particular, one *HBG* off-target site (HBG-OT1) was not only captured by both multiplex and monoplex EndoV-seq, but also validated in vivo by target site deep sequencing (Fig. 2d and Supplementary Table 31). Collectively, these data demonstrate the utility of multiplex EndoV-seq in detecting off-target sites of ABE.

### Improving the specificity of ABE by gRNA engineering

**Fig. 4:**
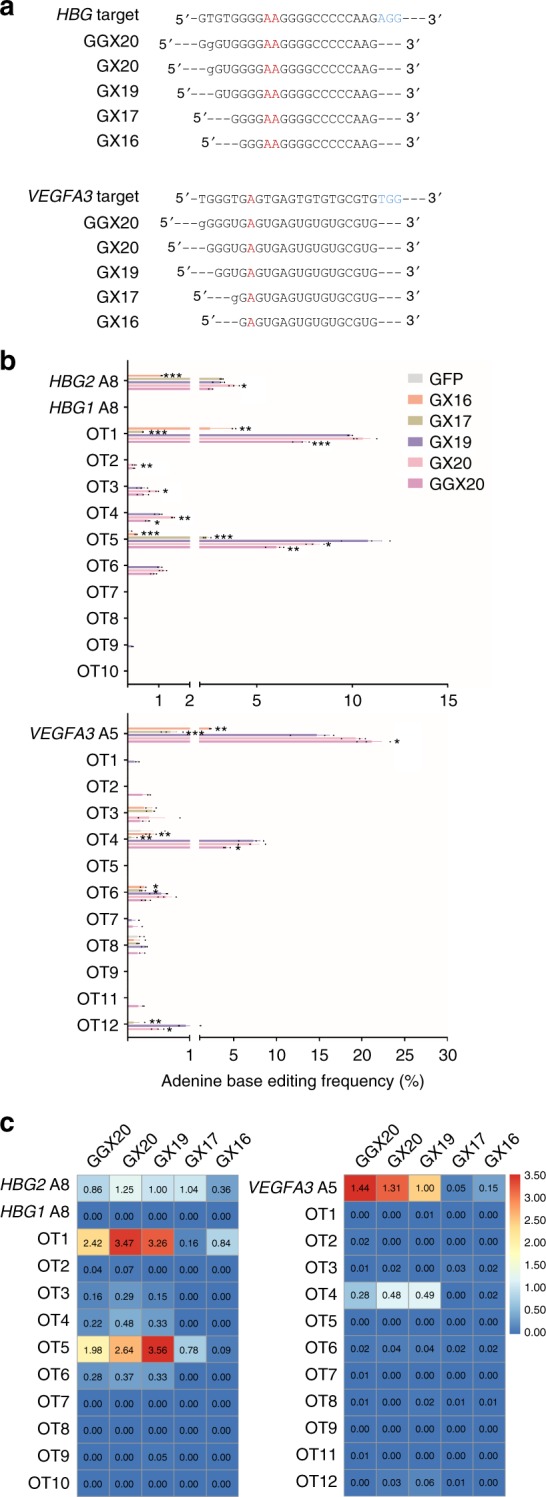
The length of gRNAs affects ABE7.10 specificity. **a** *HBG* and *VEGFA3* gRNAs of different length were designed based on the 20-mer gRNA (GX19) validated in Fig. 3d. Mismatched bases are in lower case. Target bases are in red. PAM sequences are in blue. **b** HEK-293T cells were co-transfected with the ABE7.10 expression vector and individual *HBG* or *VEGFA3* gRNAs from **a**. Genomic DNA was then extracted for target site deep sequencing. GFP-transfected cells were used as controls. The frequencies of A-to-G conversion for each gRNA at both on-target and top potential off-target (OT) sites were calculated. OT10 of *VEGFA3* was failed to be amplified. Error bars represent SEM (*n* = 3). Statistical significance was calculated using a two-tailed unpaired *t-*test. *p < 0.05; **p < 0.01; and ***p < 0.001. Source data are provided as a Source Data file. **c** The relative activity at each site was calculated by normalizing the A-to-G conversion frequency at that site to the on-target frequency of the GX19 gRNA. The ratios are presented as heat maps where higher values correspond to higher activities

Published reports have shown that extended or truncated gRNAs can improve the specificity of Cas9 and BE3^18,22,29,30^. We decided to investigate how gRNA length might affect the specificity of ABE. Based on the *HBG* and *VEGFA3* gRNAs with validated off-targets sites from Fig. 2d (named GX19 here), we generated two extended gRNAs with additional 5′ extra guanines (GX20 and GGX20) and two 5′ truncated gRNAs (GX17 and GX16) (Fig. 4a)^16,31^. The 5′ extra guanines in the extended gRNAs may become mismatched after hybridization with the target site (Fig. 4a)^16,31^. These gRNAs were individually co-expressed with ABE7.10 in human HEK-293T cells for deep sequencing and calculation of both on-target and off-target A-to-G conversion efficiency (Fig. 4b). Although *HBG1* and *HBG2* have identical target site sequences for the gRNA, we only found A-to-G conversion at the latter site, consistent with our findings in Fig. 2d and suggesting possible differential accessibility of these two sites in HEK-293T cells (Fig. 4b).

When the editing efficiency at each site was normalized to the on-target site of the original GX19 gRNA (Fig. 4c), both *HBG* extended gRNAs (GGX20 and GX20) appeared to retain high on-target conversion efficiencies. While *HBG* GGX20 had similar or lower off-target conversion compared to GX19, GX20 led to increased conversion at several off-target sites (OT1, OT2, OT3, OT4), perhaps a reflection of its higher overall efficiency. Truncated *HBG* gRNA GX16 had diminished activities at both on-target and off-target sites (Fig. 4c); in comparison, *HBG* GX17 registered no change in on-target activity while showing decreased efficiencies at all the off-target sites tested (Fig. 4c). Similarly for *VEGFA3* gRNAs, extensions preserved or increased on-target efficiencies and mostly decreased off-target deamination (Fig. 4c). Notably, truncating *VEGFA3* gRNAs essentially abolished conversion activity at all sites, suggesting stricter gRNA length requirement of ABE at this target site. Collectively, these results demonstrate that the specificity of ABE may be improved through selective modification of gRNA length without sacrificing its on-target efficiency.

## Discussion

The rapid development of gene editing tools has revolutionized both basic and clinical research. Application of such tools remains hampered by their off-target effects, especially regarding disease gene therapy, which necessitates continued efforts to develop sensitive and robust methodologies to study genome-wide off-target effects^32,33^. Approaches that enable analysis of genome-wide off-targets are therefore of broad interest and should prove invaluable to improving gene editing efficacy and specificity. We report here the development of EndoV-seq because the variety of methods that have been reported so far (e.g., ChIP-seq, HTGTS, IDLV, BLESS, GUIDE-seq, Digenome-seq, CICRLE-seq, SITE-seq, and BLISS^16,22,34–44^) cannot be used to study the genome-wide off-target effects of ABE.

Similar to Digenome-seq, EndoV-seq relies on enzymes to process modified DNA in vitro, and can be used to profile various ABE variants (e.g., ABE6.3/7.8/7.9 and xCas9-ABE)^8,14^. We show here that EndoV-seq is a robust in vitro assay to probe potential off-target sites on naked DNA. Since certain epigenetic modifications and chromatin structures may prevent access by ABE, EndoV-seq eliminates possible complications from steric hindrance and may in fact overestimate the number of potential off-targets, as not all of the EndoV-seq detected off-targets may be edited in vivo^26–28^. EndoV-seq provides a list of potential off-target sites that should prove particularly informative for in vivo off-target effect investigation and gRNA design. Our evidence indicates that EndoV-seq could identify off-target sites that were deaminated by ABE in vivo at very low efficiency (0.13%), and exhibits sensitivities comparable to Digenome-seq in dilution assays. Whether more sequencing depth can further improve the sensitivity of EndoV-seq warrants investigation. It should be noted that EndoV-seq cannot detect deamination at sites not nicked by Cas9 nickase on the complementary strand.

Our EndoV-seq data indicate that ABE7.10 is highly specific, with far fewer off-targets than canonical CRISPR/Cas9 (8.0 vs. 160.7 on average). In vivo validation found only nine bona fide off-target sites for the eight tested gRNAs. In addition, of another 100 sites that contain base A within the deamination window and had been detected only by Cas9 or BE3 Digenome-seq using the six gRNAs (*HBG*, *VEGFA3*, *HEK293-2*, *RNF2*, *HBB -28(T* *>* *C)*, and mouse *Dmd*), none appeared to be edited by ABE in cells. Taken together, these data demonstrate that ABE off-target sites are rare. Similar to other gene editing enzymes, modifying gRNA length could further improve ABE specificity. Additionally, using Cas9 variants with higher specificity or split Cas9^14,25,45–51^, optimizing reagent delivery (ribonucleoprotein complexes vs. DNA)^25,52–56^, and adjusting exposure time of genomic DNA targets to editors (e.g., an inducible system) should help enhance ABE specificity and reduce possible off-targets^57–59^.

## Methods

### Vectors

pcDNA3.1(-)-ABE7.10, pET42b-ABE7.10, and pET28a-His-Cas9 were synthesized by Guangzhou IGE biotechnology Ltd. pET42b-BE3 was purchased from Addgene. pUC19-Cas9 gRNA expression vector was generated previously^7^. Primers used for gRNA cloning into the pUC19-Cas9 gRNA expression vector are listed in Supplementary Table 32. For in vitro transcription of gRNAs, PCR amplicons of pUC19-Cas9 gRNA expression vectors (primers listed in Supplementary Table 33) were used with the MEGAshortscript T7 kit (Life Technologies).

### Cell culture and transfection

HEK-293T cells (ATCC) and mouse embryonic fibroblasts (MEFs) were cultured in DMEM supplemented with 10% FBS. The mouse embryonic fibroblasts were isolated from 13.5-day B6 mouse embryo. For transfection, pcDNA3.1(-)-ABE7.10 (1.2 µg) and pUC19-Cas9 gRNA expression plasmids (0.6 µg) were transfected into HEK-293T cells (12-well plates, 2.5 × 10^5^/well) using PEI (Sigma-Aldrich) or into MEFs (1 × 10^5^ cells) using the Amaxa 4D-Nucleofector system (Lonza). Genomic DNA was isolated 72 h after transfection using the DNeasy Blood & Tissue Kit (Qiagen) for on-target and off-target site PCR amplification. All transfection and deep-sequencing assays were repeated ≥3 times.

### Protein expression and purification

His-tagged recombinant proteins were purified as previously reported with minor modifications^25^. Briefly, BL21 Star^TM^ (DE3) *E. coli* cells (Thermo Fisher) transformed with pET42b-ABE7.10, pET42b-BE3, or pET28a-His-Cas9 were cultured overnight until OD_600_ of 0.5–0.6 before addition of IPTG (0.5 mM) and induction at 18 °C for 14–16 h. For ABE7.10 and BE3, cells were lysed in lysis buffer (100 mM Tris–HCl, pH 8.0, 1 M NaCl, 20% glycerol, 5 mM tris(2-carboxyethyl)phosphine (TCEP; Sigma-Aldrich), 20 mM imidazole (Sigma-Aldrich), and protease inhibitors) followed by sonication. The supernatant was then incubated with Ni-NTA agarose resin (GE Healthcare) and washed in wash buffer (100 mM Tris–HCl, pH 8.0, 0.5 M NaCl, 20% glycerol, 5 mM TCEP, and 20 mM imidazole) before elution (100 mM Tris–HCl, pH 8.0, 0.5 M NaCl, 20% glycerol, 5 mM TCEP, and 270 mM imidazole). For Cas9, cells lysis and resin washing were carried out using the same buffer (50 mM Tris–HCl, pH 8.0, 0.5 M NaCl, 5% glycerol, 20 mM imidazole, and protease inhibitors) before elution of Cas9 proteins (50 mM Tris–HCl, pH 8.0, 0.5 M NaCl, 5% glycerol, and 270 mM imidazole). All proteins were further purified on a 5 mL Hi-Trap HP SP cation exchange column (GE Healthcare), concentrated with the Microcon-30 kDa Centrifugal Filter Unit (30 kDa cutoff) (EMD Millipore), sterile filtered (0.22 μm PVDF membrane) (EMD Millipore), and quantified using the Reducing Agent Compatible Bicinchoninic acid assay (Pierce Biotechnology). The purified proteins were aliquoted and snap-frozen in liquid nitrogen for storage at −80 °C.

### ABE deamination and EndoV treatment

PCR products (100 ng) amplified from the target sites (primers listed in Supplementary Table 34) were incubated with 300 nM recombinant ABE7.10, 900 nM gRNA, and 2 µL 10 × NEB Buffer 3 (NEB) in a 20 µL reaction at 37 °C for 3 h. The reaction mixture was purified using the PCR Cleanup Kit (Qiagen) before incubation with EndoV (Thermo Fisher) (1 U per 100 ng of PCR products) at 65 °C for 30 min. The digested PCR products were resolved on a 3% agarose gel.

Genomic DNA (10 µg) (purified using the DNeasy Blood & Tissue Kit (Qiagen)) was incubated with 300 nM recombinant ABE7.10, 900 nM gRNA, and 50 µL 10 × NEB buffer 3 in 500 µL reaction for 8 h at 37 °C. Following RNase A (50 µg/mL) and proteinase K (20 mg/mL) treatment, inosine-containing genomic DNA was extracted with phenol:chloroform:isoamyl alcohol (Sigama) and ethanol precipitated. The purified DNA (4 µg) was then incubated with EndoV (eight units) in 100 µL reaction at 65 °C for 3 h. The resultant products were again extracted with phenol:chloroform:isoamyl alcohol (Sigma) and ethanol precipitated. Target sites were then PCR-amplified for sequencing analysis. Intact gene copy numbers were quantified by qPCR using the KAPA SYBR FAST Universal qPCR kit (KAPA Biosystems, KK4601) with primers listed in Supplementary Table 35.

### Assessment of BE3 and Cas9 activity

For BE3 deamination and USER enzyme treatment, 10 µg Genomic DNA was incubated with 300 nM recombinant BE3 proteins, 900 nM gRNA, and 50 µL 10 × NEBuffer 3 (NEB) in 500 µL reaction for 8 h at 37 °C. After RNase A (50 µg/mL) and proteinase K (20 mg/mL) treatment, uracil-containing genomic DNA was extracted with phenol:chloroform:isoamyl alcohol (Sigma), ethanol precipitated, and then incubated with USER enzyme (6 U per 4 µg of DNA) (NEB) in 100 µL reaction at 37 °C for 3 h. As described in the section above, USER-digested products were similarly extracted and precipitated, target sites PCR amplified and sequenced, and intact gene copy numbers quantified by qPCR (primers listed in Supplementary Table 35).

For Cas9 cleavage, DNA sequences containing the target sites were amplified using primers listed in Supplementary Table 34, and the products (100 ng) treated with 300 nM recombinant Cas9, 900 nM gRNA, and 1 µL 10 × NEBuffer 3 (NEB) in a 10 µL reaction volume at 37 °C for 3 h. The digested products were resolved on a 3% agarose gel. Alternatively, 10 µg genomic DNA was incubated with 300 nM recombinant Cas9, 900 nM gRNA, and 50 µL 10 × NEBuffer 3 (NEB) in 500 µL reaction for 8 h at 37 °C. The reaction mixtures were similarly extracted and processed as described above for intact gene copy number analysis by qPCR using primers listed in Supplementary Table 35.

### Whole genome and deep sequencing

Whole-genome sequencing was carried out using the Novaseq 6000 sequencing system (Illumina) at HaploX Biotechnology Co., Ltd. Genomic DNA (1 µg) was fragmented (to 400–500 bp), blunt end repaired, and sequenced at 30–40× depth. Genomic sites were scored using Digenome 2.0 as reported by Kim et al.^22^. PCR products were deep sequenced using the Hiseq 2000 (Illumina) as paired-end 150 reads. Sequence reads were aligned to reference sequences by BWA with default parameters (v0.7.13). Samtools (v1.3, http://samtools.sourceforge.net) and Picard tools (v2.2.2, http://picard.sourceforge.net) were used to build indices and sort reads. VarScan (v2.4.2, mpileup2snp and mpileup2indel with—min-reads 2 10—min-var-freq 0.01) was used to call variants for all samples and SelectVariants was used to divide the combined variants into indels and SNVs.

### Data analysis and scoring

For DNA cleavage score calculation, genomic DNA cleavage was assessed using the Digenome 2.0 tool (http://www.rgenome.net/digenome-js/standalone) as described by Kim et al.^16,17,22^. For each target position (*i*), the sequencing depth (*D*_*i*_) at position *i*, and the numbers of forward (*F*_*i*_) and reverse (*R*_*i*_) sequencing reads starting at position *i* were calculated. These three values were then used to calculate the cleavage score for each target position (*i*) as follows:

$$\begin{array}{l}\mathop {\sum}\limits_{a = 1}^5 {\frac{{\left( {F_i - 1} \right)}}{{D_i}}} \times \frac{{\left( {R_{i + 8 + \alpha } - 1} \right)}}{{D_i}} \times \left( {F_i + R_{i + 8 + \alpha } - 2} \right)\\ + \mathop {\sum}\limits_{a = 1}^5 {\frac{{\left( {R_{i + 11} - 1} \right)}}{{D_{i + 11}}} \times \frac{{\left( {F_{i - 3 + \alpha } - 1} \right)}}{{D_{i - 3 + \alpha }}} \times \left( {R_{i + 11} + F_{i - 3 + \alpha } - 2} \right)} \end{array}$$

Site Allocator (Supplementary Software 1) was developed based on the program described for Cas9 multiplex Digenome-seq^18^, and was used to calculate the edit distance (or sequence similarity) between different gRNA target sites. Edit distance was measured by the Levenshtein algorithm from Python that shows the similarity between two strings, and aggregates the minimum edit steps (insertion = 1, deletion = 1, and substitution = 1) required to transform one string into the other.

### Code availability

Site Allocator, the open-source Python package for multiplex EndoV-seq data analysis, is provided as Supplementary Software 1.

### Reporting summary

Further information on experimental design is available in the Nature Research Reporting Summary linked to this article.

## Supplementary information

Supplementary Information

Description of Additional Supplementary Files

Supplementary Software 1

Source Data

Reporting Summary

## Data Availability

The deep sequencing data from this study have been deposited in the NCBI Sequence Read Archive database under Accession Number SRP169835 [ftp://ftp-trace.ncbi.nlm.nih.gov/sra/review/SRP169835_20181120_135510_cb5ae17636e975f9bf71ddf5bc542075]. All other relevant data are available upon request. ABE7.10 plasmids for prokaryotic protein expression are available from Addgene with accession ID 120398 (pET42b-ABE7.10). ABE7.10 plasmids for mammalian protein expression are available from Addgene with accession ID 120399 (pcDNA3.1(-)-ABE7.10). The source data for Figs. 1b, 2d, 4b and Supplementary Figures 1, 2, 3b, 4, 6a, 8, 9 and 10a are provided as a Source Data file.
