# Supplementary material for: Genome-wide profiling of adenine base editor specificity by EndoV-seq
Source: Nat Commun. 2019 Jan 8;10:67. doi: 10.1038/s41467-018-07988-z (PMC6325126; doi:10.1038/s41467-018-07988-z)
Supplement: Supplementary file 1 — Supplementary Information [file 41467_2018_7988_MOESM1_ESM.pdf]

Genome-wide Profiling of Adenine Base Editor Specificity by EndoV-seq

Liang *et al.*

Supplementary Information

Supplementary Figure 1a

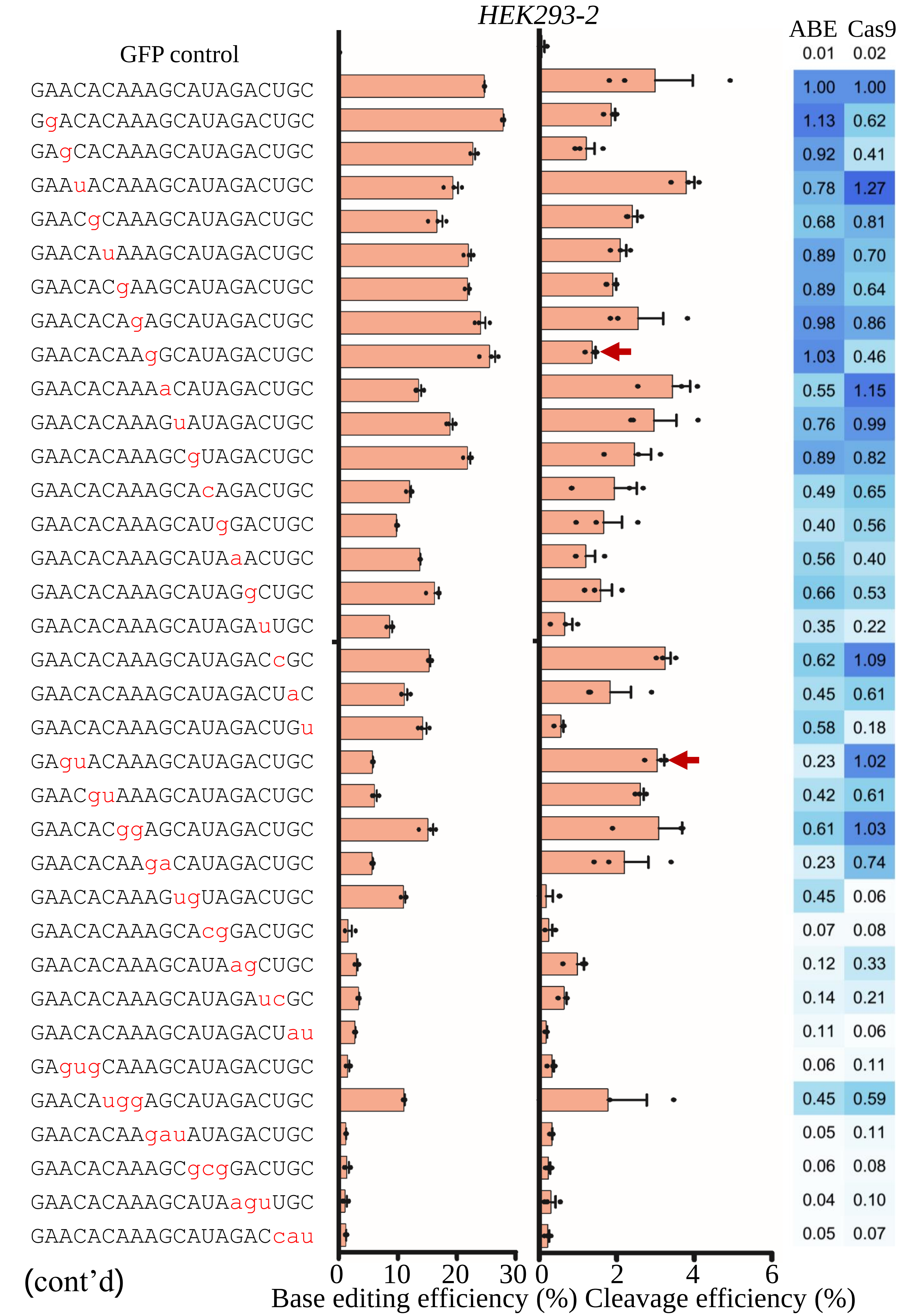

(cont’d)

Supplementary Figure 1b

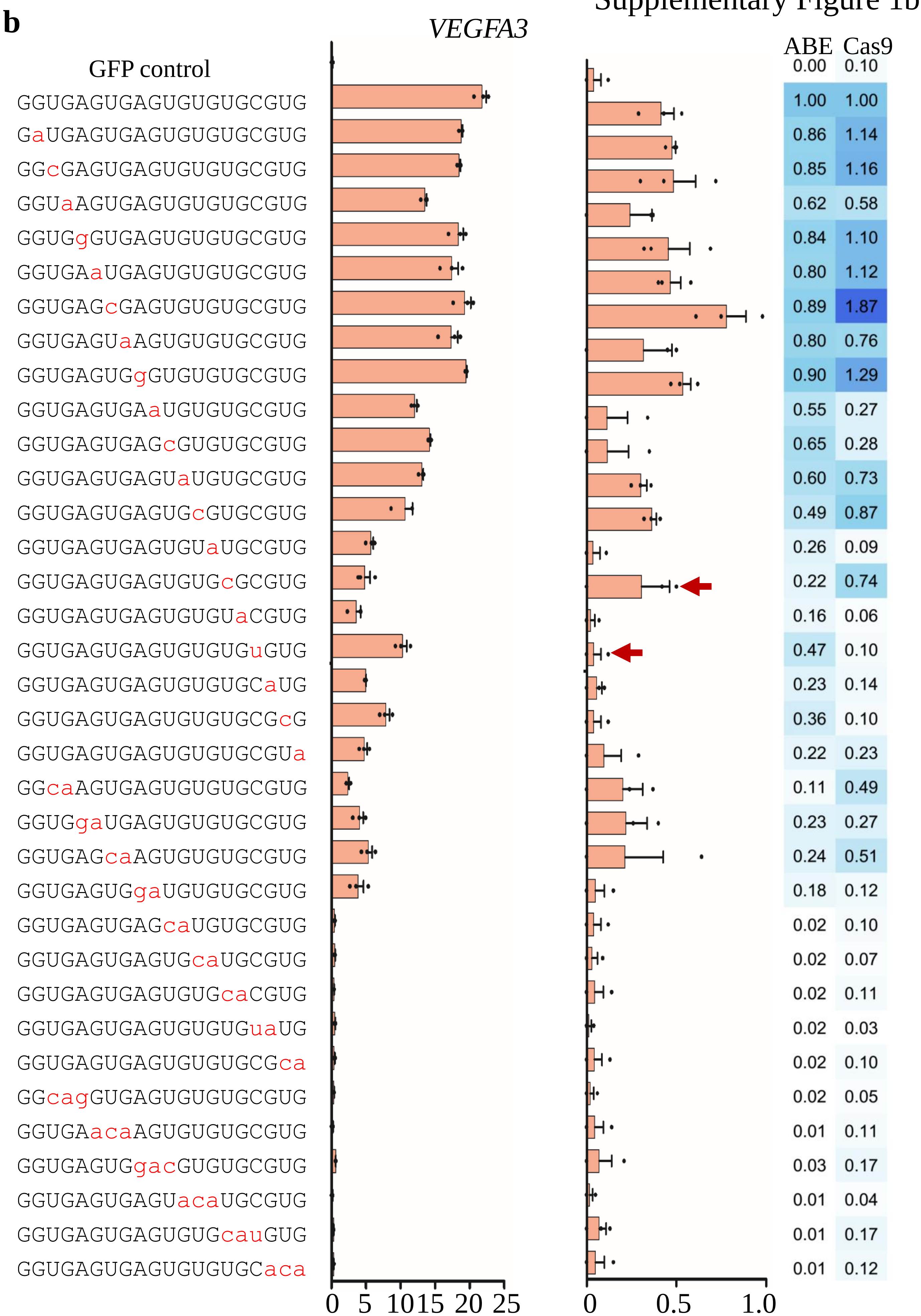

(cont'd)

c

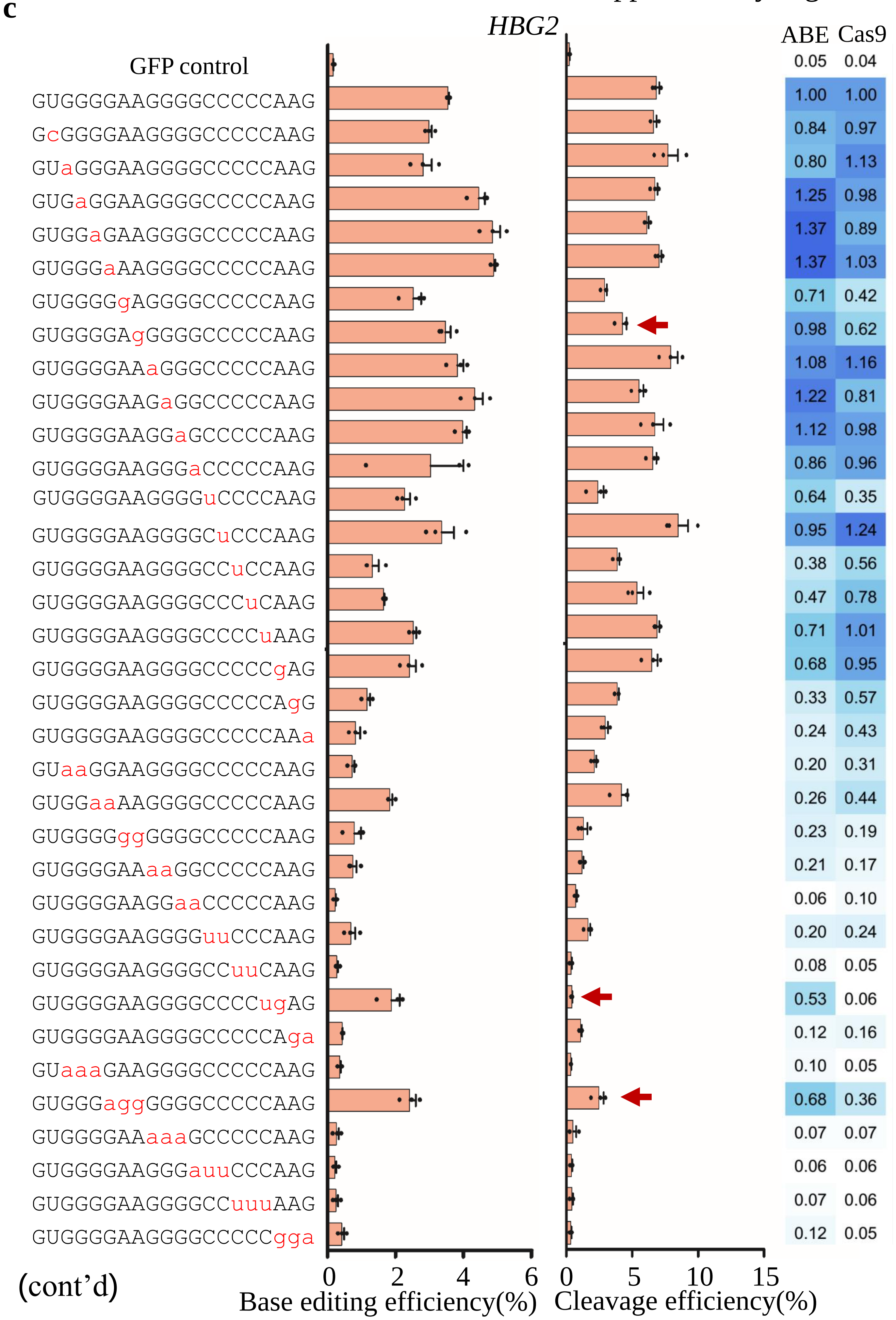
HBG2
ABE Cas9
GFP control 0.05 0.04
GUGGGGAAGGGGCCCCCAAG 1.00 1.00
GcGGGGAAGGGGCCCCCAAG 0.84 0.97
GUaGGGAAGGGGCCCCCAAG 0.80 1.13
GUGaGGAAGGGGCCCCCAAG 1.25 0.98
GUGGaGAAGGGGCCCCCAAG 1.37 0.89
GUGGGaAAGGGGCCCCCAAG 1.37 1.03
GUGGGGgAGGGGCCCCCAAG 0.71 0.42
GUGGGGAgGGGGCCCCCAAG 0.98 0.62
GUGGGGAAaGGGCCCCCAAG 1.08 1.16
GUGGGGAAGaGGCCCCCAAG 1.22 0.81
GUGGGGAAGGaGCCCCCAAG 1.12 0.98
GUGGGGAAGGGaCCCCCAAG 0.86 0.96
GUGGGGAAGGGGuCCCCAAG 0.64 0.35
GUGGGGAAGGGGCuCCCAAG 0.95 1.24
GUGGGGAAGGGGCCuCCAAG 0.38 0.56
GUGGGGAAGGGGCCCuCAAG 0.47 0.78
GUGGGGAAGGGGCCCCuAAG 0.71 1.01
GUGGGGAAGGGGCCCCCgAG 0.68 0.95
GUGGGGAAGGGGCCCCCAgG 0.33 0.57
GUGGGGAAGGGGCCCCCAAa 0.24 0.43
GUaaGGAAGGGGCCCCCAAG 0.20 0.31
GUGGaaAAGGGGCCCCCAAG 0.26 0.44
GUGGGGggGGGGCCCCCAAG 0.23 0.19
GUGGGGAAaaGGCCCCCAAG 0.21 0.17
GUGGGGAAGGaaCCCCCAAG 0.06 0.10
GUGGGGAAGGGGuuCCCAAG 0.20 0.24
GUGGGGAAGGGGCCuuCAAG 0.08 0.05
GUGGGGAAGGGGCCCCugAG 0.53 0.06
GUGGGGAAGGGGCCCCCAga 0.12 0.16
GUaaaGAAGGGGCCCCCAAG 0.10 0.05
GUGGGaggGGGGCCCCCAAG 0.68 0.36
GUGGGGAAaaaGCCCCCAAG 0.07 0.07
GUGGGGAAGGGauuCCCAAG 0.06 0.06
GUGGGGAAGGGGCCuuuAAG 0.07 0.06
GUGGGGAAGGGGCCCCCgga 0.12 0.05
0 2 4 6
Base editing efficiency(%)
0 5 10 15
Cleavage efficiency(%)

(cont'd)

Supplementary Figure 1. ABE tolerates mismatches between gRNA and genomic DNA.

A series of mismatched gRNAs (with 1-3 base transitions in the 20-nt guide sequence) targeting *HEK293-2* (**a**) or *VEGFA3* (**b**) or *HBG2* (**c**) were co-transfected into 293T cells with expression vectors encoding ABE7.10 or Cas9. Genomic DNA was then extracted from the cells and processed for deep sequencing. ABE base editing efficiency and Cas9 cleavage efficiency were calculated and plotted as shown. Relative activity of each mismatched gRNA compared to the canonical gRNA was also calculated and shown on the far right. Mismatched bases are indicated by red lower case letters. Red arrows indicate gRNAs with very significant different efficiencies between ABE7.10 and Cas9. GFP transfected cells were used as controls. Error bars represent SEM (n=3). Source data are provided as a Source Data file.

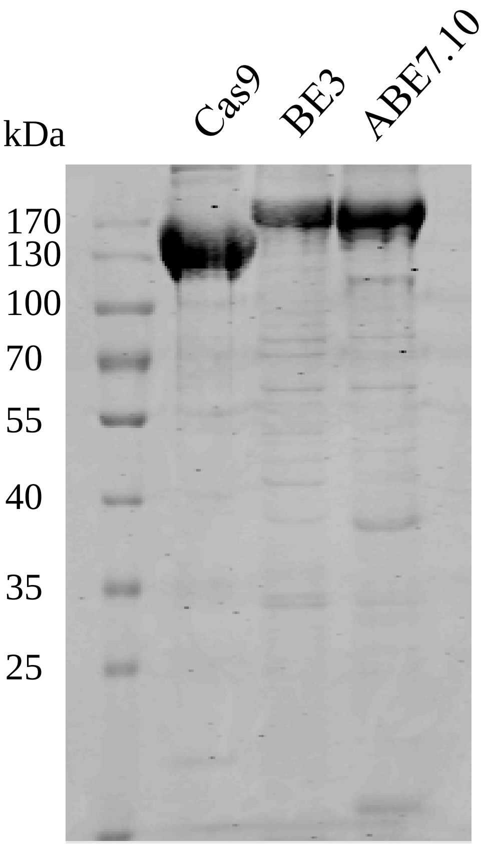

Supplementary Figure 2. Recombinant Cas9, BE3, and ABE7.10 proteins were purified from bacteria and examined by SDS-PAGE and Coomassie staining. Source data are provided as a Source Data file.

a

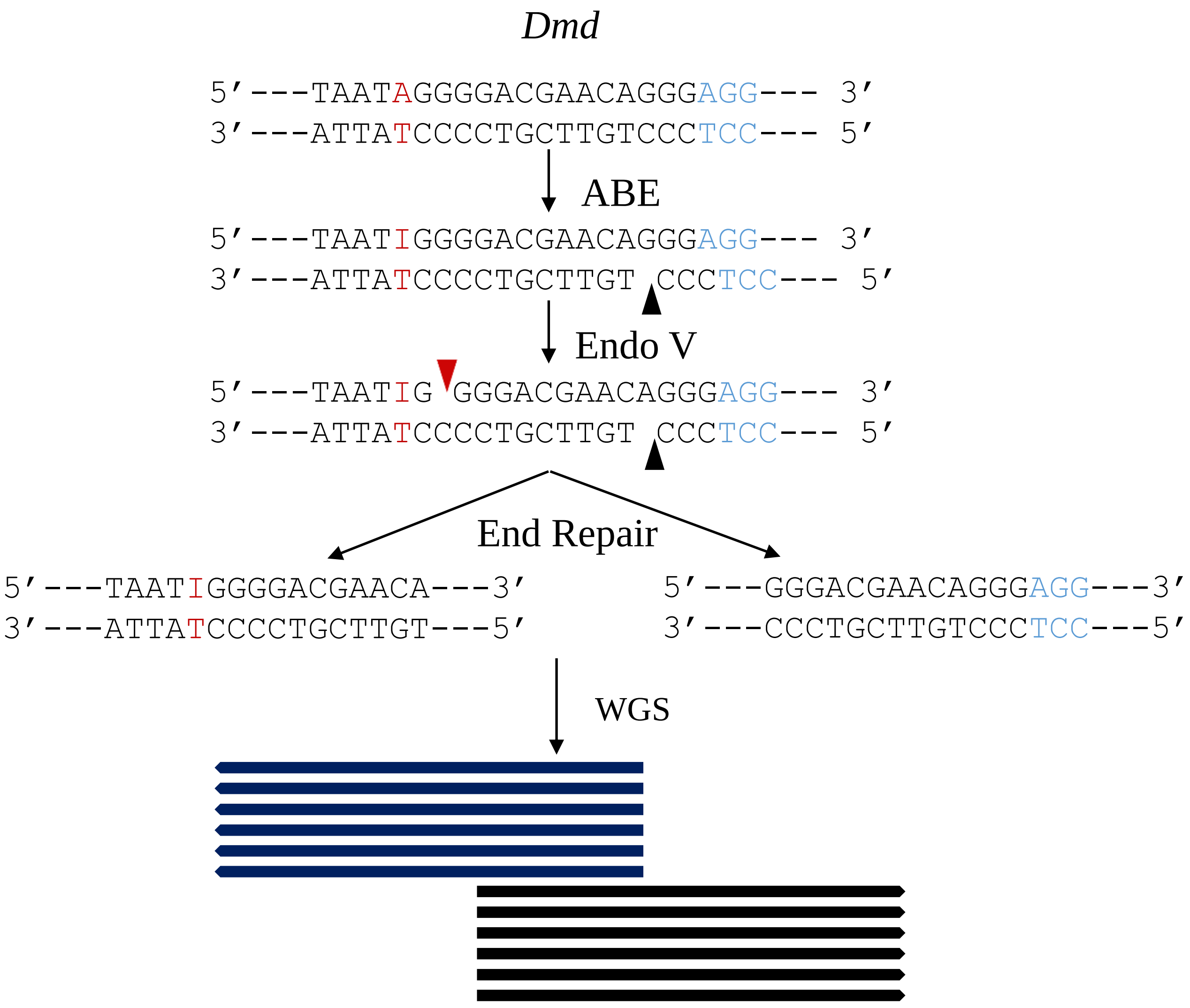
Dmd
5′---TAATAGGGGACGAACAGGGAGG--- 3′
3′---ATTATCCCCTGCTTGTCCCTCC--- 5′
ABE
5′---TAATIGGGGACGAACAGGGAGG--- 3′
3′---ATTATCCCCTGCTTGT CCCTCC--- 5′
Endo V
5′---TAATIG GGGACGAACAGGGAGG--- 3′
3′---ATTATCCCCTGCTTGT CCCTCC--- 5′
End Repair
5′---TAATIGGGGACGAACA---3′
3′---ATTATCCCCTGCTTGT---5′
5′---GGGACGAACAGGGAGG---3′
3′---CCCTGCTTGTCCCTCC---5′
WGS

b

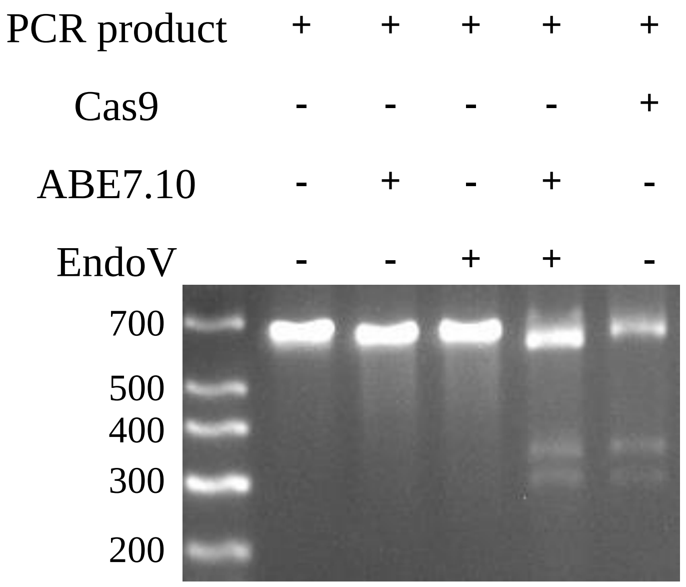
PCR product + + + + +
Cas9 - - - - +
ABE7.10 - + - + -
EndoV - - + + -
700
500
400
300
200

(cont'd)

c

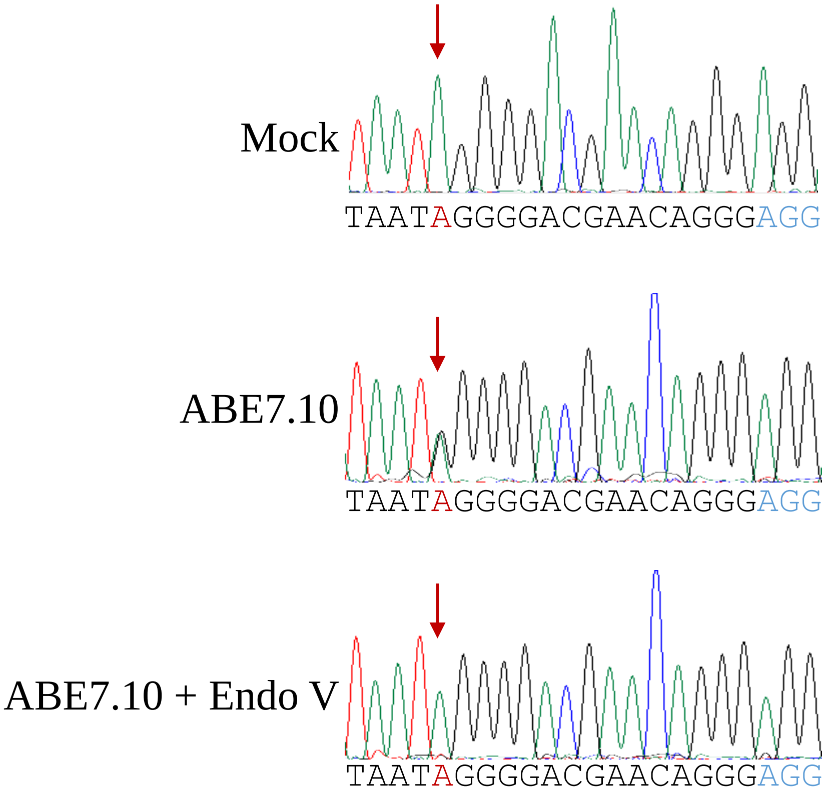

d

Mock	TAATAGGGGACGAACAGGGAGG	100%
ABE7.10	TAATAGGGGACGAACAGGGAGG	70.1%
	TAATGGGGGACGAACAGGGAGG	29.9%
ABE7.10 + Endo V	TAATAGGGGACGAACAGGGAGG	100%

e

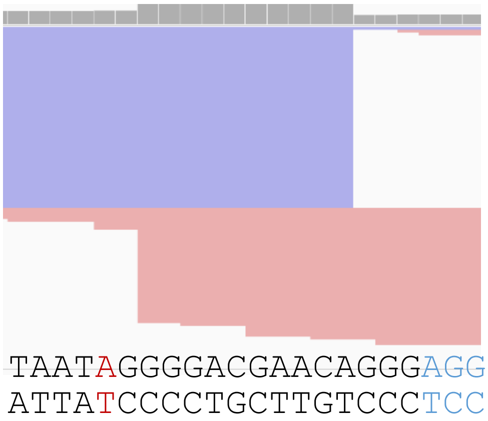

(cont'd)

Supplementary Figure 3. EndoV-seq profiling of genome-wide off-target effects of ABE7.10 using the mouse *Dmd* gRNA.

(**a**) A flow chart for assessing *in vitro* ABE activity by EndoV-seq is shown, using sequences from the mouse *Dmd* site as an example. Genomic DNA is first incubated with recombinant ABE7.10 and the appropriate gRNA and then digested with EndoV, thereby allowing the DNA to be nicked by both nCas9 nickase (black triangle) and EndoV (red triangle, one residue downstream of base I). The cleaved DNA is subsequently fragmented and end repaired for whole-genome sequencing (WGS) with ~30-40 fold coverage.

(**b**) Genomic DNA of mouse embryonic fibroblast cells was used to PCR amplify regions spanning the mouse *Dmd* site. The PCR products (100 ng) were incubated with ABE7.10 (300nM) and HEK293-2 gRNA (900nM) first (3hrs) before EndoV (1U) incubation (30 mins). The treated products were resolved by agarose gel electroporesis. Recombinant Cas9 was used as a positive control for DNA cleavage. Source data are provided as a Source Data file.

(**c**) Genomic DNA (10 μg) was incubated with ABE7.10 (300nM) and *Dmd* gRNA (900nM) for 8 hours before ± EndoV (8U) incubation (3hrs). Sanger sequencing chromatograms of PCR products of the *Dmd* gRNA target region amplified from the treated DNA are shown. Mock treated genomic DNA served as a control. PAM, blue. Target base A, red. Peaks on the chromatograph, green for A, red for T, blue for C, and black for G.

(**d**) PCR products from (c) were deep sequenced. The frequency of each allele is shown on the right. PAM, blue. Target base A, red.

(**e**) Alignment of whole-genome sequencing reads of the *Dmd* gRNA target region as visualized by the Integrative Genomics Viewer (IGV). Target base A, red. PAM, blue.

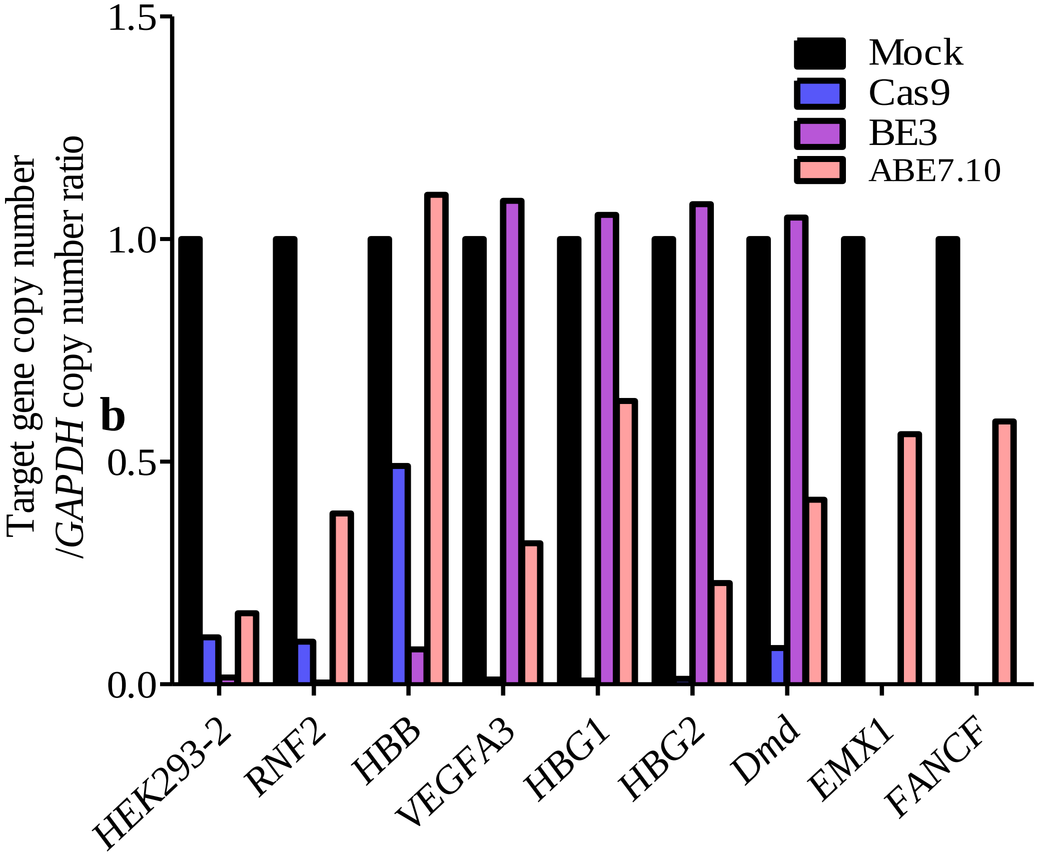

Supplementary Figure 4. Efficient cleavage of target sites by ABE7.10 compared to SpCas9 and BE3.

Genomic DNA (10 µg) was incubated with ABE7.10 (300nM) and gRNA (900nM) for 8 hours before EndoV (8U) incubation (3 hours). qPCR analysis was then used to determine the copy number of various target genes. GAPDH sequences were also amplified as internal control.

For BE3, genomic DNA (10 µg) was incubated with BE3 (300nM) and gRNA (900nM) for 8 hours before USER (8U) incubation (3 hours). For Cas9, genomic DNA (10 µg) was incubated with Cas9 (300nM) and gRNA (900nM) for 8 hours.

For *EMX1* and *FANCF*, only ABE EndoV-seq was performed. Source data are provided as a Source Data file.

Supplementary Figure 5a-b

a

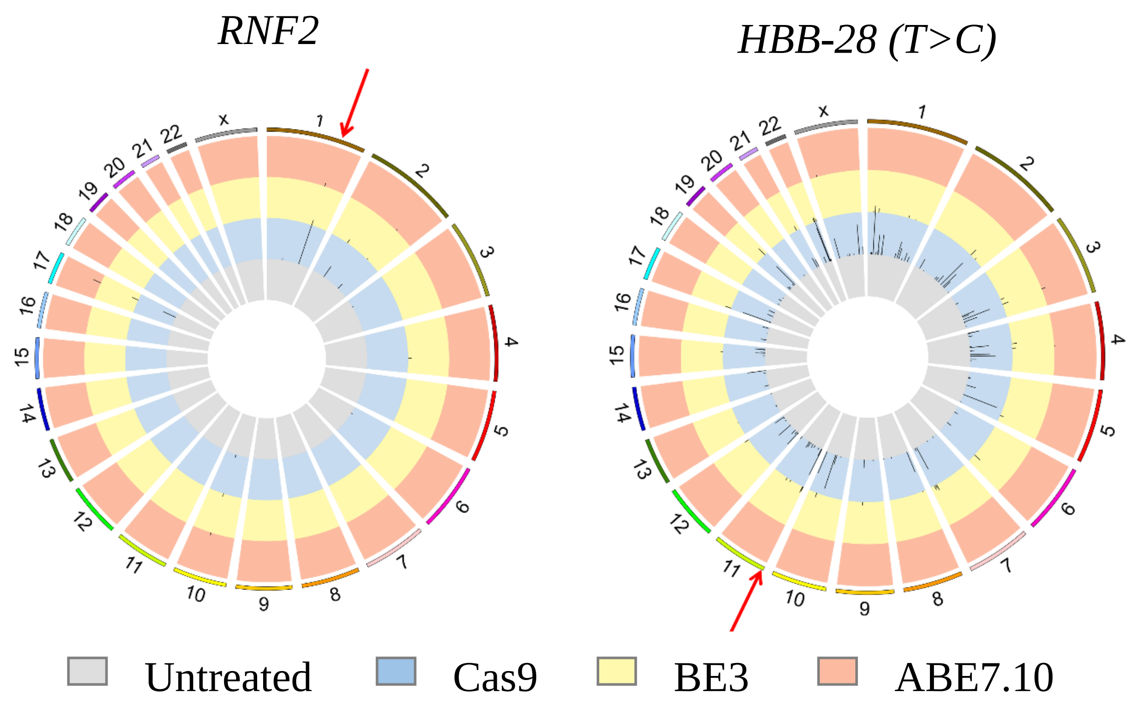

b

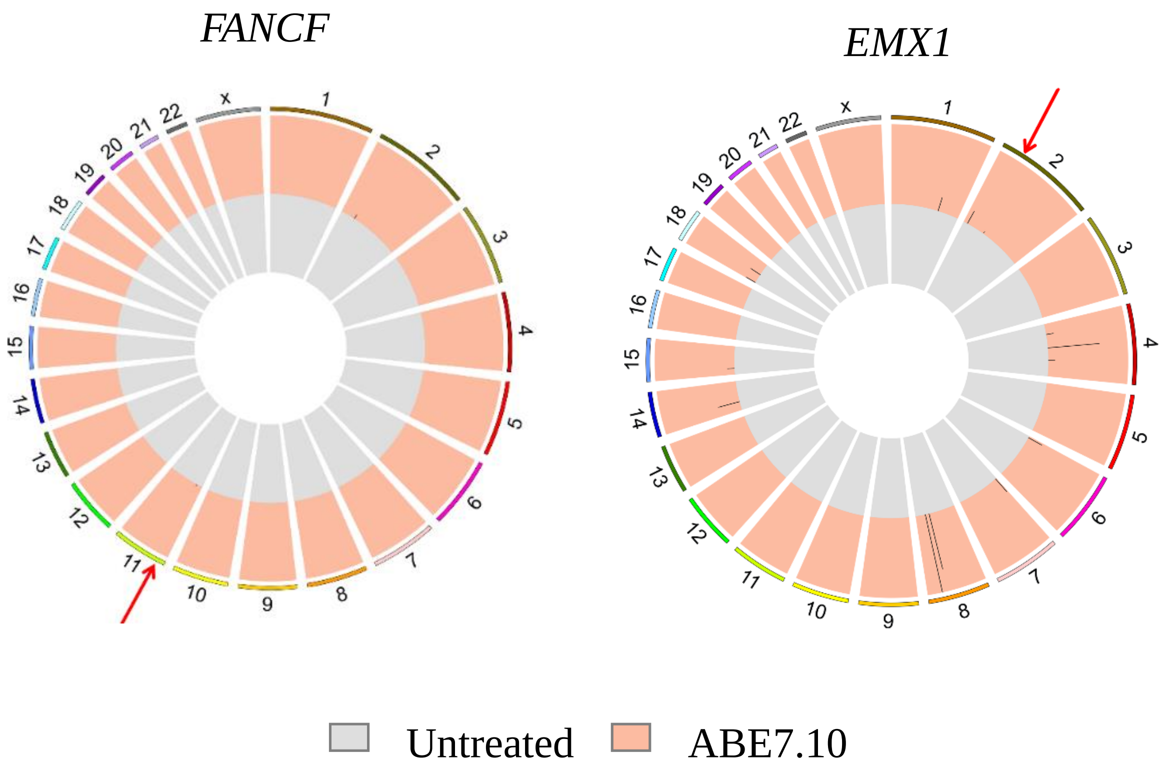

(cont'd)

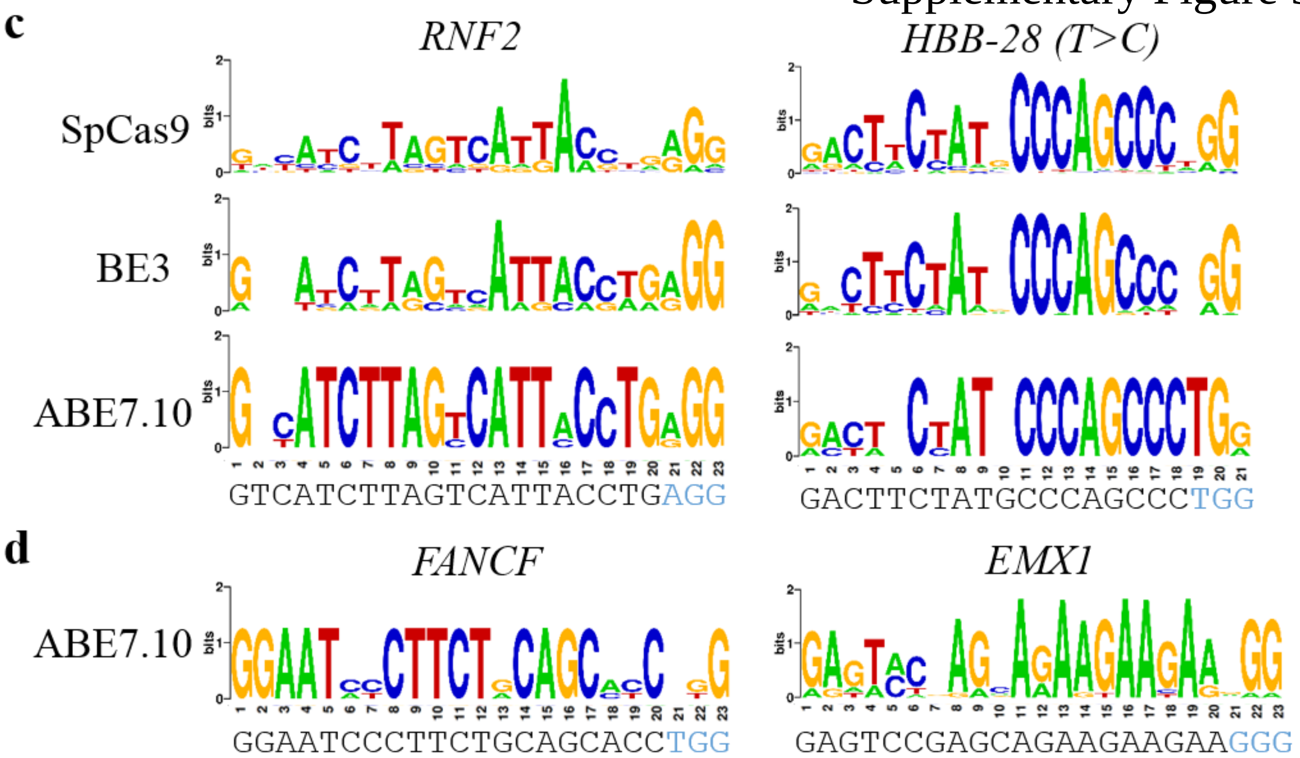

Supplementary Figure 5. Profiling genome-wide off-target effects of ABE.

(a) Genome-wide cleavage scores (cutoff score of >2.5) (Circos plots) of DNA treated with Cas9 (blue), BE3 (yellow), or ABE7.10 (coral) along with the human *RNF2* and *HBB*-28(T>C) gRNAs. Untreated samples (gray) served as controls. Red arrows, on-target sites. Note: The length of *HBB*-28(T>C) gRNA is 18-nt.

(**b**) EndoV-seq analysis using the human *FANC*F and *EMX1* gRNAs (coral). Untreated samples (gray) served as controls. Genome-wide cleavage scores are presented with a cutoff score of >2.5. Red arrow indicates the on-target site.

(**c**) WebLogo sequence logos of EndoV-captured ABE off-targets (with cutoff scores of >2.5) and on-target sites for *RNF2* and *HBB*-28 (T>C) gRNAs. EndoV results were compared to those from Digenome-seq of Cas9 or BE3 (with cutoff scores of >2.5). Target sequence is shown below with PAM in blue.

(**d**) WebLogo sequence logos of EndoV-captured off-targets (with cutoff scores of >2.5) and on-target sites for *FANC*F and *EMX1* gRNAs. Target sequences are shown with PAM in blue.

a

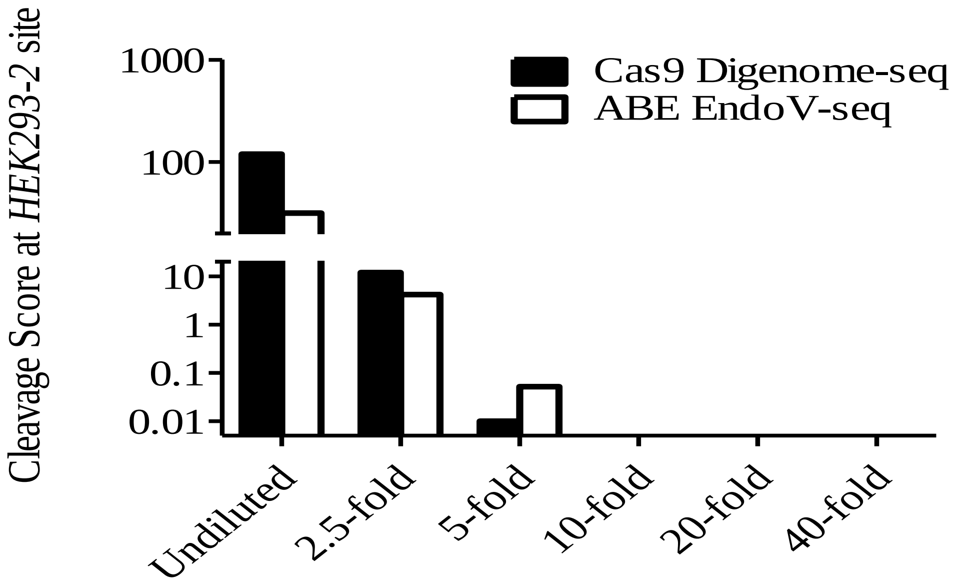

b

	Undiluted	Fold Dilution				
		2.5	5	10	20	40
**Cas9 Digenome-seq**	+	+	-	-	-	-
**ABE EndoV-seq**	+	+	-	-	-	-

Supplementary Figure 6. Comparable sensitivity of ABE EndoV-seq and Cas9 Digenome-seq.

Genomic DNA (10 μg) treated with ABE7.10 (300nM) and *HEK293-2* gRNA (900nM) (8 hours) was diluted with untreated genomic DNA as indicated before EndoV (8U) incubation (3 hours) and whole-genome sequencing. For Cas9, genomic DNA (10 μg) treated with Cas9 (300nM) and gRNA (900nM) (8 hours) was similarly diluted with untreated before whole-genome sequencing. *GAPDH* sequences were amplified by qPCR as internal controls. Cleavage scores at the gRNA target site were plotted in (**a**), and detection of on-target editing by Cas9 Digenome-seq and ABE EndoV-seq was summarized in (**b**). +, score > 0.1. -, score < 0.1. Source data are provided as a Source Data file.

Supplementary Figure 7a

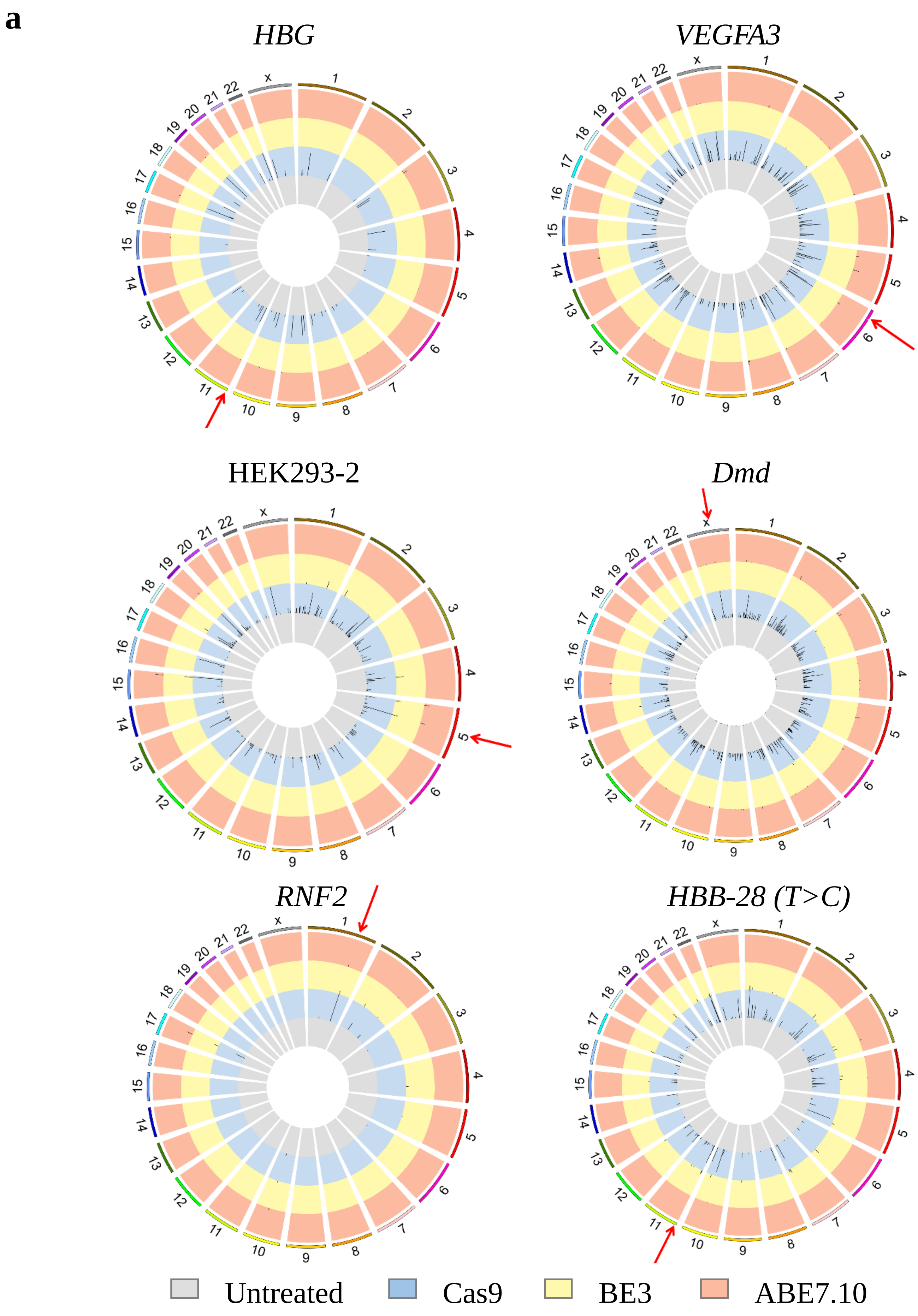

(cont'd)

Supplementary Figure 7b-c

b

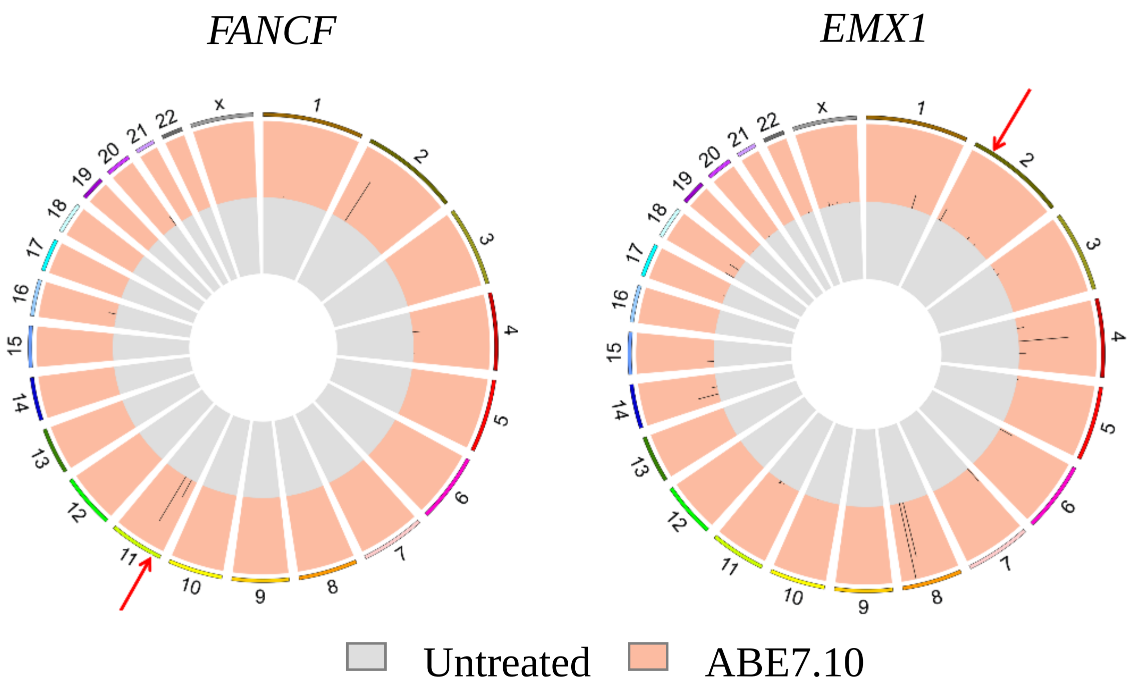

c

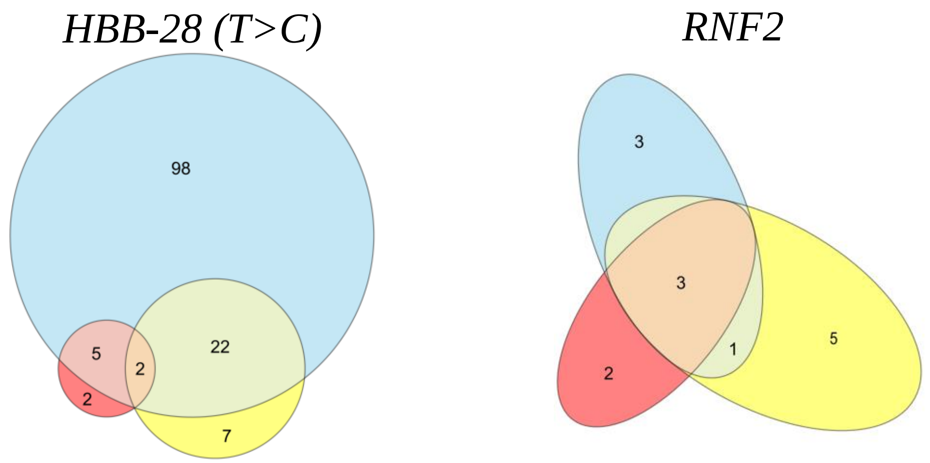

(cont'd)

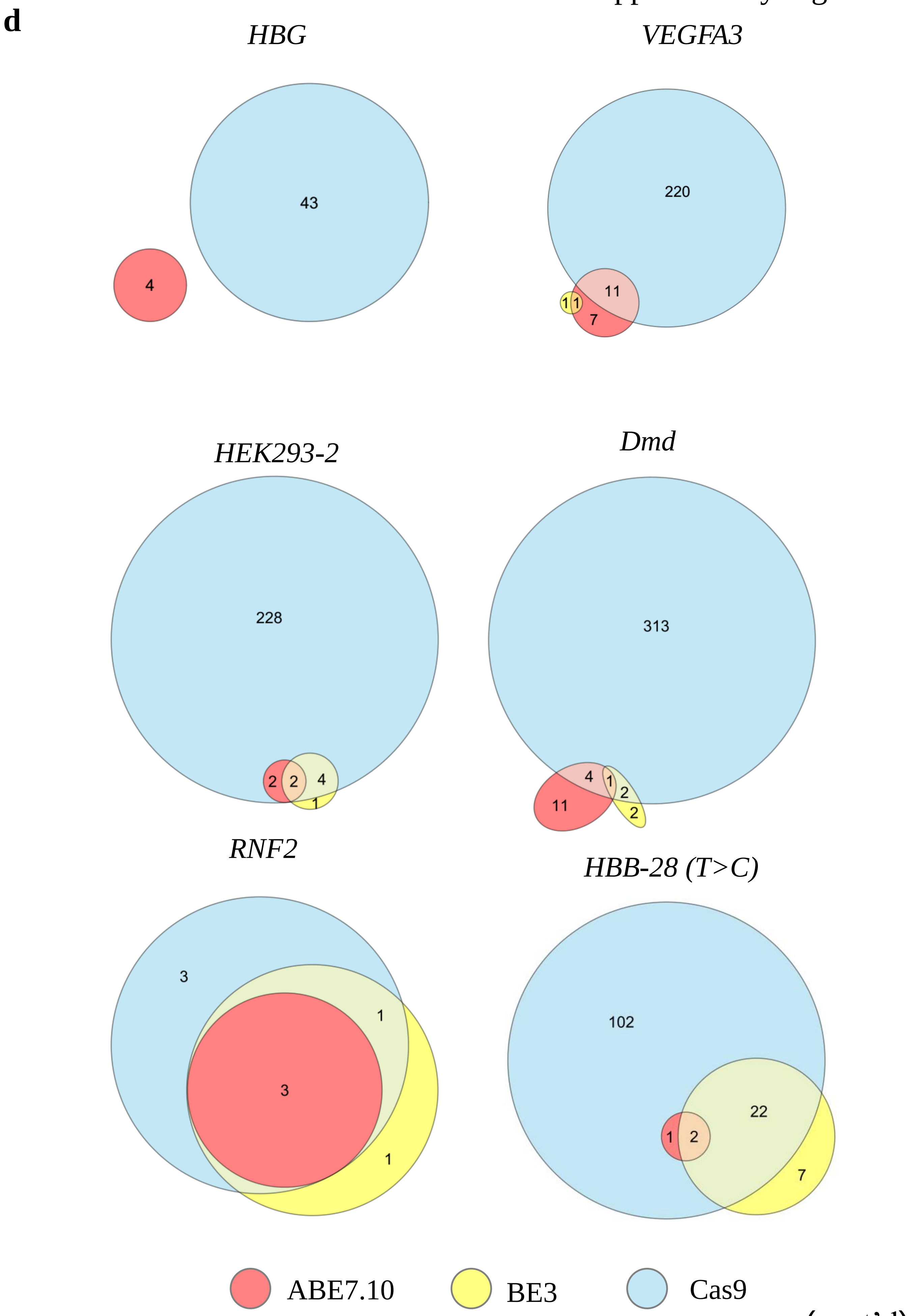
d
HBG
43
4
VEGFA3
220
11
1
1
7
HEK293-2
228
2
2
4
1
Dmd
313
4
1
2
11
2
RNF2
3
1
3
1
HBB-28 (T>C)
102
22
1
2
7
ABE7.10
BE3
Cas9

(cont'd)

Supplementary Figure 7. Profiling genome-wide off-target effects of ABE.

(**a**) Circos plots showing genome-wide cleavage scores of DNA treated with Cas9 (blue), BE3 (yellow), or ABE7.10 (coral) along with the indicated gRNA. Untreated DNA (gray) served as a control. On-target sites are indicated with red arrow. Cutoff scores of >0.1 for ABE7.10 and BE3, and >2.5 for Cas9.

(**b**) EndoV-seq analysis was carried out using the human *FANCF* and *EMX1* gRNAs (coral). Untreated samples (gray) served as controls. Genome-wide cleavage scores are presented as Circos plots, with a cutoff score of >0.1. Red arrow indicates the on-target site.

(**c**) Venn diagram that compares Digenome-captured sites for Cas9 and BE3 with EndoV-seq captured sites of ABE7.10. Cutoff scores of >0.1 for ABE7.10 and BE3, and >2.5 for Cas9.

(**d**) Venn diagram that compares Digenome-captured sites for Cas9 and BE3 with EndoV-seq captured sites of ABE7.10. Cutoff scores of >2.5 for ABE7.10, BE3, and Cas9.

a

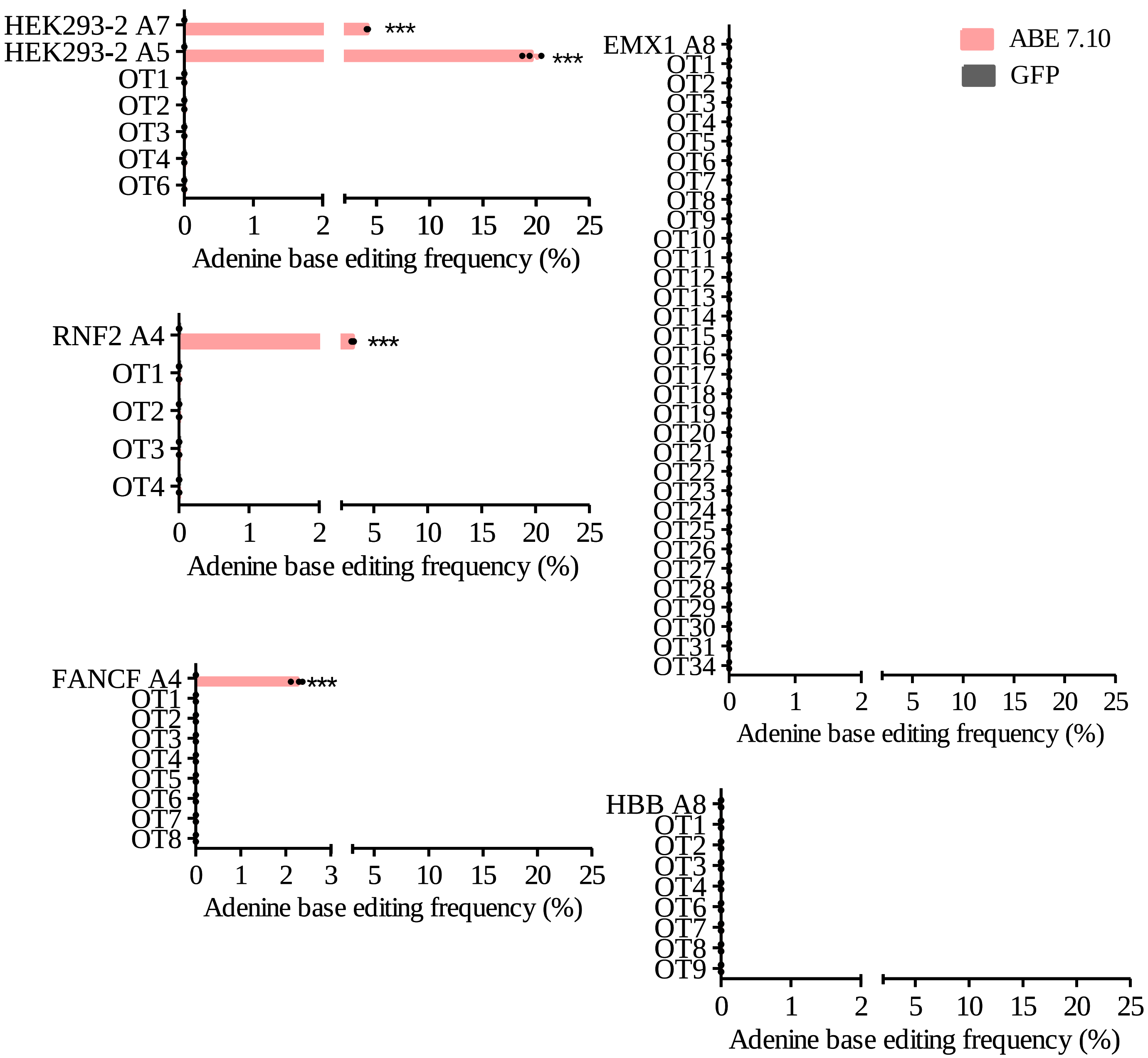
HEK293-2 A7
HEK293-2 A5
OT1
OT2
OT3
OT4
OT6
***
***
0 1 2 5 10 15 20 25
Adenine base editing frequency (%)
RNF2 A4
OT1
OT2
OT3
OT4
***
0 1 2 5 10 15 20 25
Adenine base editing frequency (%)
FANCF A4
OT1
OT2
OT3
OT4
OT5
OT6
OT7
OT8
***
0 1 2 3 5 10 15 20 25
Adenine base editing frequency (%)
ABE 7.10
GFP
EMX1 A8
OT1
OT2
OT3
OT4
OT5
OT6
OT7
OT8
OT9
OT10
OT11
OT12
OT13
OT14
OT15
OT16
OT17
OT18
OT19
OT20
OT21
OT22
OT23
OT24
OT25
OT26
OT27
OT28
OT29
OT30
OT31
OT34
0 1 2 5 10 15 20 25
Adenine base editing frequency (%)
HBB A8
OT1
OT2
OT3
OT4
OT6
OT7
OT8
OT9
0 1 2 5 10 15 20 25
Adenine base editing frequency (%)

(cont'd)

b

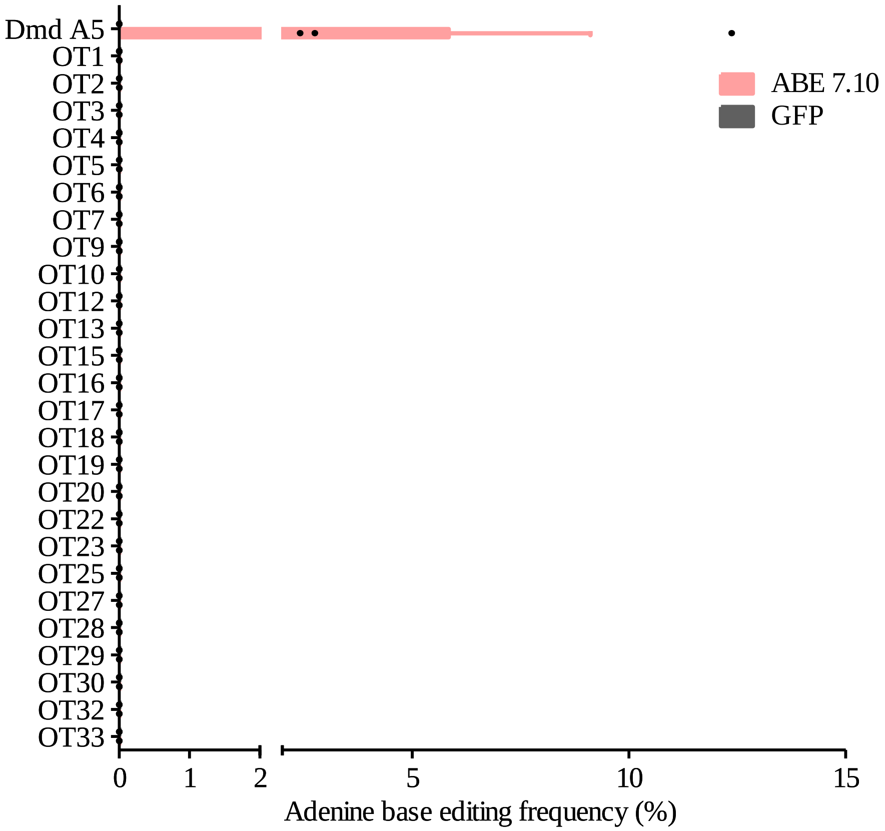

Supplementary Figure 8. Validation of EndoV-seq captured ABE7.10 sites by deep sequencing. Error bars represent SEM (n=3). Statistical significance was calculated using the two-tailed unpaired *t* test (***p < 0.001). Source data are provided as a Source Data file.

(a) Genomic DNA was extracted from HEK293T cells co-expressing ABE7.10 and various gRNAs for PCR amplification and deep sequencing. GFP transfected cells were used as controls. Both the on-target and off-target (OT) sites were examined. Cutoff scores of >0.1 for ABE7.10.

(b) Genomic DNA was extracted from MEFs co-expressing ABE7.10 and the *Dmd* gRNA for PCR amplification and deep sequencing. GFP transfected cells were used as controls. Both the on-target (*Dmd* A5) and top off-target (OT) sites were examined. Cutoff scores of >1 for ABE7.10.

Supplementary Figure 9a

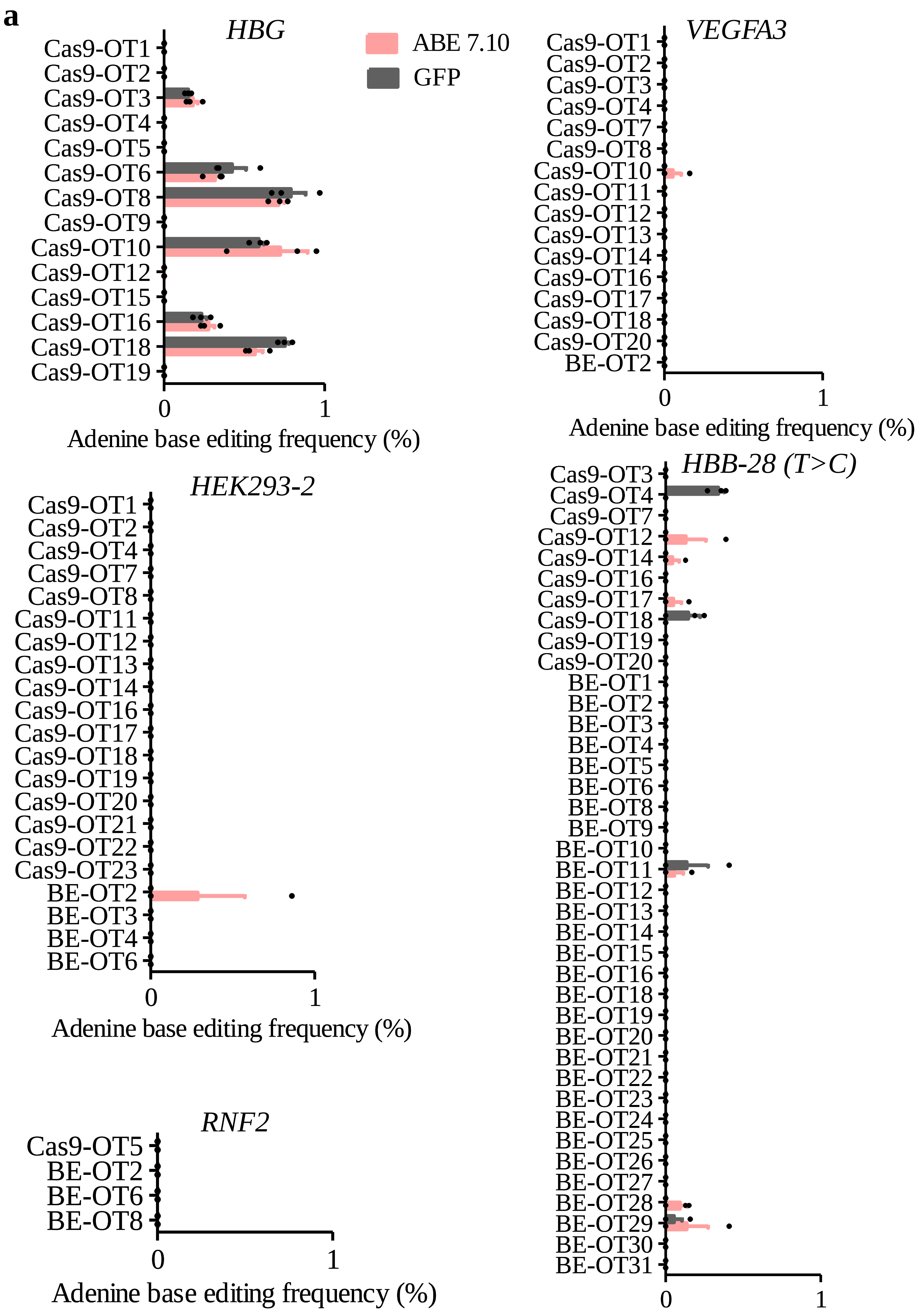

(cont’d)

b

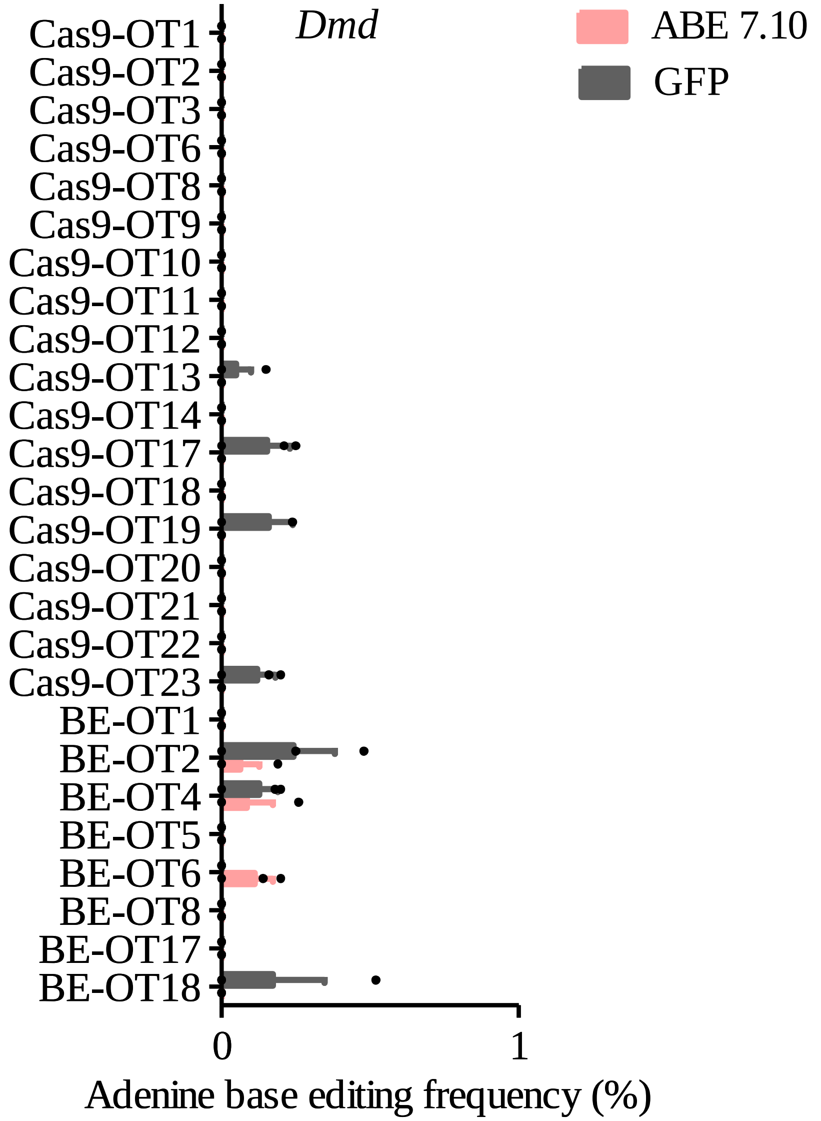

Supplementary Figure 9. Off-target deamination by ABE at sites identified by Cas9 or BE3 Digenome-seq only. Error bars represent SEM (n=3). Statistical significance was calculated using the two-tailed unpaired *t* test. No significant difference was found ($p > 0.05$). Source data are provided as a Source Data file.

(a) Genomic DNA was extracted from HEK293T cells co-expressing ABE7.10 and various gRNAs for PCR amplification and deep sequencing. GFP transfected cells were used as controls. Off-target (OT) sites unique for BE3 and Cas9 were examined.

(b) Genomic DNA was extracted from MEFs co-expressing ABE7.10 and the *Dmd* gRNA for PCR amplification and deep sequencing. GFP transfected cells were used as controls. Off-target (OT) sites unique for BE3 and Cas9 were examined.

Supplementary Figure 10

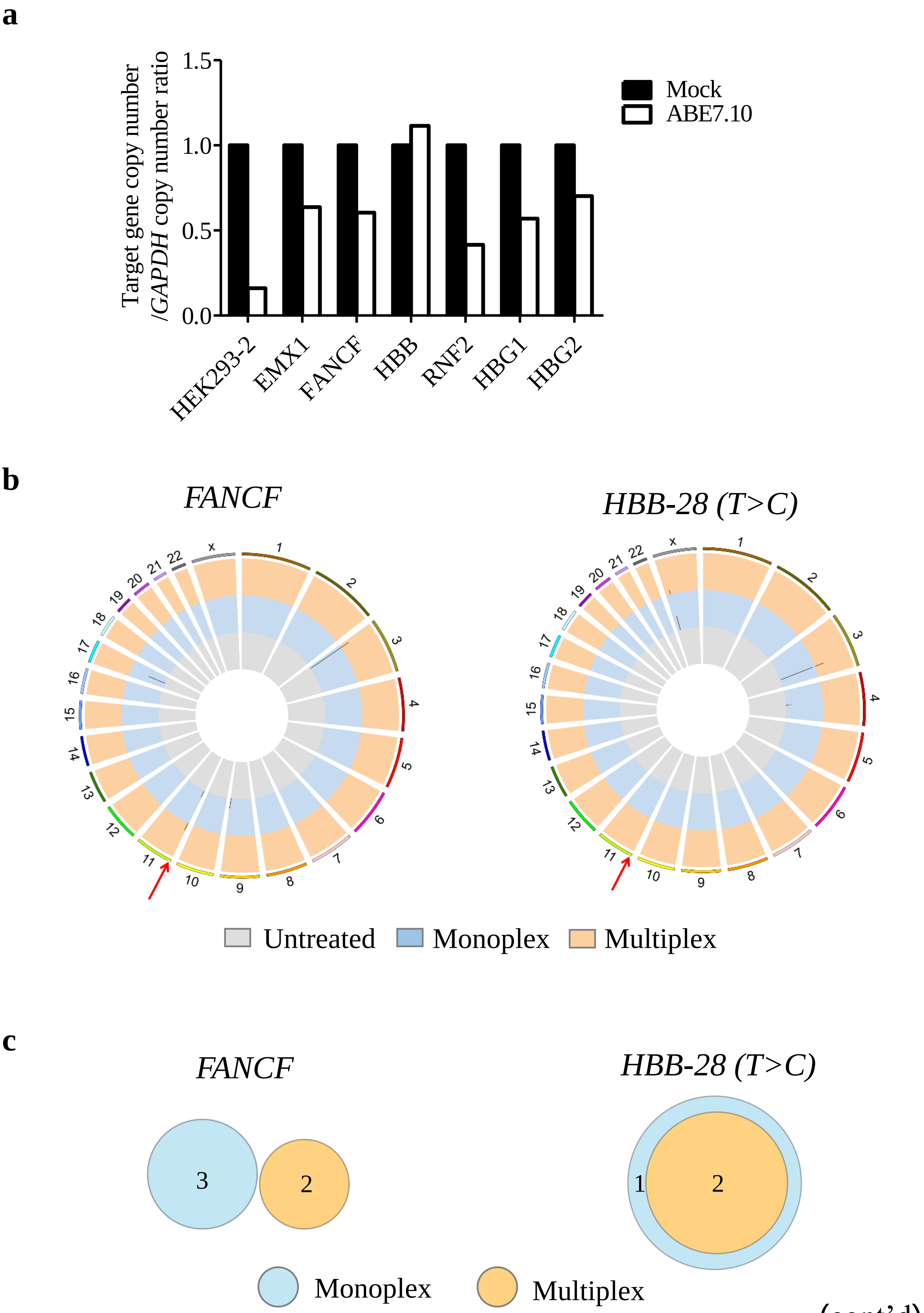

(cont'd)

Supplementary Figure 10. Using multiplex EndoV-seq to examine ABE activity and specificity.

(**a**) Genomic DNA (10 μg) was first incubated with recombinant ABE7.10 (300 nM) and 6 different gRNAs (200 nM each) targeting *HEK293-2*, *EMX1*, *FANCF*, *HBB-28 (T>C)*, *RNF2*, and *HBG* for 8 hours, and then with EndoV (8 U) for 3 hours. qPCR analysis was then carried out to determine the copy number of various target genes (intact alleles). *GAPDH* sequences were also amplified as internal control. Source data are provided as a Source Data file.

(**b**) Circos plots showing genome-wide DNA cleavage scores (cutoff score of >2.5) of untreated sample (gray), monoplex EndoV-seq (blue), and multiplex EndoV-seq (orange) for human *FANCF* and *HBB-28 (T>C)* gRNAs. On-target sites are indicated by red arrows.

(**c**) Venn diagrams comparing off-target sites captured by monoplex and multiplex EndoV-seq (cleavage score of > 2.5, on target sites not shown).

Supplementary Table 1. EndoV-seq captured sites using HBG gRNA and ABE7.10

Site name	Position	DNA sequence at cleavage site	DNA cleavage score			Bulge	Deep seq primer		Validated
			ABE7.10	BE3	Cas9		FP	RP	ABE7.10
HBG1-TA	chr11:5271278	GTGGGGAAGGGGCCCCCAAGAGG	2.0		42.3		TCCTGGTATCCTCTATGATGGGA	GTGGAGTTTAGCCAGGGACC	No
HBG2-TA	chr11:5276202	GTGGGGAAGGGGCCCCCAAGAGG	1.4		42.4		TGGTGGGAGAAGAAAACTAGC	GTGGAGTTTAGCCAGGGACC	Yes
HBG-OT1	chr3:13705838	GgtGGGAtGGGGtCCCCAAGTGG	11.4				ATGGCTGCAAATCCAAGGGT	AAATGCTTCTCGGGCTCTCC	Yes
HBG-OT2	chr17:35304222	GgtaGGgAGaGGCCCCCAgaGGG	4.8				GAGGTTGAAACTCCTCGCCA	GGAATTAAGATGCAACTGAGAGTAC[illegible]	No
HBG-OT3	chr9:138419302	GgtGGGgAGcGGCCCCCcAGTGG	2.8				GGCTGTCCCTGGTTGTCTGG	CGAGCACTGAGGCCTGGTTA	Yes
HBG-OT4	chr15:84049561	agtGGGgAGGcGCCCtCAAGTGG	2.5				GTGCCTTGGCTTGCTATTTGG	TTCTGGGAGGAGAGTTGGGGT	Yes
HBG-OT5	chr10:73282210	GTGGGG-AGtGGCCCCCAAGAGG	2.2			RNA bulge	TATTATATCCATTCCAGTGGGTTT[illegible]	CACACAGATGTAAGTGGTAAGAGCA[illegible]	Yes
HBG-OT6	chr9:138419300	GTGGGG-AGcGGCCCCCcAGTGG	2.2			RNA bulge	GCAGAAAGTGTAGCGGGTGAC	CTTCCTGAGCAGCTGATTGGT	Yes
HBG-OT7	chr19:33976063	GTGaGGGgAGGGaCCCtCAAGAGG	2.1			DNA bulge	ACTGGCTTCTTTCCGGGC	GCTGGGATTACAGGTGTGAGTC	No
HBG-OT8	chr7:88444871	GTGGGGAAaGGtGCCCaCAAGGGG	2.0			DNA bulge	GTCCCTTGTATGTAACCAATCTCA[illegible]	TGTCAGAGCCTGAGAAGGTGAA	No
HBG-OT9	chr3:4746491	aTGaGGAAGcGaCCCCCAAGAGG	1.7				GTTGATGTCTGTGGAACGGT	GTACACCCGTCACAGCTAGA	Yes
HBG-OT10	chr5:117819228	GTGGGGtAaGaaCCCCCAAaTGG	1.4				GATGGTGGCCCTCTTCTCACA	GGATGCCCAGACAAAAGTATGC	No
HBG-OT11	chr9:21122154	GgtGaGAAGGaGCCCaCAAGTGG	1.0		43.9		TTGTTATAGAGGAACCCCAGCC	TTTGCTTAACATACAGAGTTCCTG	No
HBG-OT12	chr3:34824512	GgtaGGAAGGGGCtCCCAAGAGG	0.7		39.4		TTCCCTTACTGATCCGTGTCC	AAACACTCTGTGAGTCACTTTTGA	No
HBG-OT13	chrX:12298826	GTGGGaAAGGacCCCCaAtGAGG	0.7		54.1		CCCATGAAGTCCCCACTGTC	CAGGTGGCTAGGCTGAAACA	No
HBG-OT14	chr5:134498847	GTGGaaAAGGaGaCCCCAAGAGG	0.5				GCGACACTTTTGCTCTACGTG	CTCCACCCTATGACACCACCT	No
HBG-OT15	chr17:7365166	GTGtG-AAGGGGCtCCCAAGGGG	0.4			RNA bulge	TCTCACACAAAGCAGCCTGA	CTTTCACCAGATGCCACCCT	No
HBG-OT16	chr10:79889820	GTGaGGAAGatGtCCCCAAGTGG	0.4				AGTGTACTTTGCAAGCAAAAGGG	TCCTGAAGTCCAGTTCCCTTCC	No
HBG-OT17	chr7:2663814	GTGaGGAcAGGGtCCCCCAAGGGG	0.4			DNA bulge	AACCTAGAGGGTGCAGGACA	TGGAAAAGGGGTGTCCAAGG	No
HBG-OT18	chr20:24950449	aTGGGGAA-GGGCCCCCAGGGGC	0.3			RNA bulge	GAAGAAGGTGACTCCGCCTC	GAGGGGTATTCCTGTCCAGC	No

Mismatched bases are in lower case. And deleted bases are indicated with dash. Inserted bases are in purple. Column is left blank when cleavage is not detected. ABE7.10 and gRNA expression vectors were cotransfected into 293T cells for validation.

Supplementary Table 2. EndoV-seq captured sites using VEGFA3 gRNA and ABE7.10

Site name	Position	DNA sequence at cleavage site	DNA cleavage score			Bulge	Deep seq primer		Validated
			ABE7.10	BE3	Cas9		FP	RP	ABE7.10
VEGFA3-TA	chr9:110103695	GGTGAGTGAGTGTGTGCGTGTGG	18.1		214.5		GTGCAGACGGCAGTCACTAGG	CTATTGGAATCCTGGAGTGACCC	Yes
VEGFA3-OT1	chr5:89440969	aGaGAGTGAGTGTGTGCaTGAGG	41.5		175.3		GTGGGACCTGGTGGGAGT	ACAATCATGGAAGAATGCAAAG	No
VEGFA3-OT2	chr8:143890817	GGTGtaTGAGTGTGTGtGTGAGG	28.7				GTGTGACTAAGTGTGAGAGTATGTG	GTGACGATTTATGCTAATGTGT	No
VEGFA3-OT3	chr5:29367379	tGTGAGTGAGTGTGTGtaTGGGG	20.3		49.1		TTTTGTTTCTAAAAATTAAACTAAG	TGTTCCATTGTCTGAAATGTAT	No
VEGFA3-OT4	chr14:65569159	aGTGAGTGAGTGTGTGtGTGGGG	15.7		111.0		GCTCATTTCCTACGGCCCAG	CTGCAGTGAGGAGGTGGTTC	Yes
VEGFA3-OT5	chr1:181557193	GGaGAGTGAGTGTGTGCaTGTGC	14.9				GTGGGTTTTGAAACCAAATGTTCT	AATTGAGCTGAAACAGGACCAG	No
VEGFA3-OT6	chr14:62078773	tGTGAGTaAGTGTGTGtGTGTGG	11.2		56.0		GCCACAGGCACTAACTTCTTCA	GATGAAGCTGCCTTTCCTAAGC	Yes
VEGFA3-OT7	chr3:193993884	aGTGAaTGAGTGTGTGtGTGTGG	10.3		72.3		CCCTTTGTGACCCAAAAGATTCC	TAAGGCACGAGTCAGGATGGG	No
VEGFA3-OT8	chr2:230506241	GGTGAGcaAGTGTGTGtGTGTGG	9.2	9.3			CAGATGCAAATGCAAAAGAACAATA	AATGGATCAGAGGACCAACATTGTA	No
VEGFA3-OT9	chr8:141037917	aGTGAGTGAGTGTGTGtGTGAAG	7.7				ATCATGGCAGAAGGGGAAGA	CACGTGTCGAGGGAGGGAC	No
VEGFA3-OT10	chr22:37662824	GcTGAGTGAGTGTaTGCGTGTGG	7.2		163.4		Failed to be amplified		
VEGFA3-OT11	chr7:152671378	aGTGAGTGAGTGaGTGaGTGAGG	7.2		101.5		TGTATTTACTCCATTCACCATCA	CCATGAAGTATGTTCCATCTGA	No
VEGFA3-OT12	chr2:177463426	GGTGAGTGtGTGTGTGCaTGTGG	7.0		76.5		GCGCTTTCCCTTTGCTAGAATC	CTCAGCAATGCTTATATTACTGGC	Yes
VEGFA3-OT13	chrX:41726218	GGTGAGTGAGTGaGTGaGTGAGG	7.0		12.5		GCATACTAGACTAGGGGTTCTGC	TTCCCAAACAGTGTGCCATGAT	No
VEGFA3-OT14	chr11:79178512	aGTGAGTGAGTGaGTGaGTGGGG	4.8		121.1		TCCTTAATGTTTTTGCATTGGAGG	GAACCTCTAGTAGGAGTCGCT	No
VEGFA3-OT15	chr16:80016320	tGTGAGTGAGTGTGTGCGTGTGA	4.0				GCATTGGGTATTTGTGTGTATAA	TGAGCCTTTGAAGTGTGTCTCT	No
VEGFA3-OT16	chr12:114752926	tGTGAGTGAGTGTGTGCaTGTGA	3.8				CCTCTCAGACATGGAAAGGG	CACACTCAAACATGCTCACACA	No
VEGFA3-OT17	chr5:150224710	GGTGAGTGAGaGTGTGtGTGTGG	3.2				ATGTTAGGGTGTGTGAACGTG	ACAGCCACTCATACCTGGTG	No
VEGFA3-OT18	chrX:56327306	tGTGAGTGtGTGTGTGCaTGTGG	3.0		72.7		ATGAACACCCACATACCCTT	TGACCTCTATTCCACTCACTTT	No
VEGFA3-OT19	chr6:157078327	GaTGAGTGAGTGaGTGaGTGGGG	2.2		145.2		AGTGTCCAGTGTTGATAAAGTCTA	TTAAAATGATTCACCTGTATAAGG	No
VEGFA3-OT20	chr14:98442523	GGTGAGTGtGTGTGTGaGTGTGG	2.0		38.8		Failed to be amplified		
VEGFA3-OT21	chrX:42430834	aGTGAGTGAGTGTGaGCGTGAAG	1.8		92.2		ACATTGCTACACCTTTGGATTCT	ACTGACAAGGTCATTTGATTGGAC	No
VEGFA3-OT22	chr5:115434669	tGTGgGTGAGTGTGTGCGTGAGG	1.8		64.3		CAATGTGATGATTTGATAGCTG	TCTAATGTATGGCATGGTGACT	No
VEGFA3-OT23	chr10:109378067	GGTGAGTGAGTGaGTGaGTGAGG	1.4		41.7		AAAGTCTGTGGTAGTGTATAGTAAT	ATATAGTATAAGAGATAAAAAATGG	No
VEGFA3-OT24	chr6:24224733	GGTGAGcGtGTGTGTGCaTGTGG	1.1		57.8		GGGGTACAATGGTGCACAGA	TGCCACCCCAGTTTTGAGTT	No
VEGFA3-OT25	chr16:12264602	aGTGAGTGAGTGTGTGtGTGTGA	1.0				Failed to be amplified		
VEGFA3-OT26	chrX:149380335	aaTGAaTGAGTGaGTGtGTGAGT	0.5				Failed to be amplified		
VEGFA3-OT27	chr11:7625795	GGTGAGTagGTGTGTGtGTGGGG	0.4				Failed to be amplified		
VEGFA3-OT28	chr10:107867368	aGaGAGTGAGTGTGTGtGTtGGG	0.3		38.4		Failed to be amplified		
VEGFA3-OT29	chr12:5100948	tGTGAaTGAGTGTGTGCaTGTGA	0.3		72.6		Failed to be amplified		
VEGFA3-OT30	chr1:47690894	tGTGAGaGAGaGTGTGCGTGTGG	0.2				Failed to be amplified		
VEGFA3-OT31	chr5:150224714	GGTGAGTGAGaGTGTGtGTGTGG	0.2				Failed to be amplified		
VEGFA3-OT32	chr1:4770551	aagtgtgtgagtgtgtgcgtGTA	0.1				Failed to be amplified		

Mismatched bases are in lower case. And deleted bases are indicated with dash. Inserted bases are in purple. Column is left blank when cleavage is not detected. ABE7.10 and gRNA expression vectors were cotransfected into 293T cells for validation.

Supplementary Table 3. EndoV-seq captured sites using HEK293-2 gRNA and ABE7.10

Site name	Position	DNA sequence at cleavage site	DNA cleavage score			Bulge	Deep seq primer		Validated
			ABE7.10	BE3	Cas9		FP	RP	ABE7.10
HEK293-2-TA	chr5:87240603	GAACACAAAGCATAGACTGCGGG	19.4	27.4	131.7		ACAATGATAACAAGACCTGGCTG	CCCCATCTGTCAAACTGTGCG	Yes
HEK293-2-OT1	chr15:93557679	GAACACA-tGCATAGACTGCTAG	35.3	37.0	126.9	RNA bulge	CTCTGAGAGTGCCGCCAG	ACTGGCGTTTACTACCTCCT	No
HEK293-2-OT2	chr5:7625827	GtACACA-AtaATAGACTGCAGG	21.7	25.2	38.4	RNA bulge	GTTTTGGTGTGAGGGCAGTTT	AAAGAACACTTCCGGGTAGC	No
HEK293-2-OT3	chr19:35505476	GAACAC-AAGCAcAGACTGaAGG	9.2	N.D.	30.2	RNA bulge	GTTCAGGCGCTGTTGCTAGA	CAGAGGAGGAAAGCAAGCTCA	No
HEK293-2-OT4	chr8:52596627	GAACACAtAGCATAGA-TatTGG	3.2	N.D.	47.9	RNA bulge	TACACTTGACAAATGGGCCAG	GCATGTGGATGCATGTGCTA	No
HEK293-2-OT5	chr5:177062733	agAttgcAAaaATtttCTcCCAT	0.8	N.D.	N.D.		Failed to be amplified		
HEK293-2-OT6	chr10:65717412	GAACAC-tctCATAcACTGCTGG	0.6	N.D.	17.4	RNA bulge	GTTGAACACTGTGAGGAACTGC	CCATTAGGATGGCTTAAATGAA	No

Mismatched bases are in lower case. And deleted bases are indicated with dash. Inserted bases are in purple. Column is left blank when cleavage is not detected. ABE7.10 and gRNA expression vectors were cotransfected into 293T cells for validation.

Supplementary Table 4. EndoV-seq captured sites using RNF2 gRNA and ABE7.10

Site name	Position	DNA sequence at cleavage site	DNA cleavage score			Bulge	Deep seq primer		Validated
			ABE7.10	BE3	Cas9		FP	RP	ABE7.10
RNF2-TA	chr1:185056773	GTCATCTTAGTCATTACCTGAGG	12.1	19.3	160.1		AACGGAACTCAACCATTAAGCA	CCAACATACAGAAGTCAGGAATGC	Yes
RNF2-OT1	chr17:53928587	GTCATCTTAGTCATTAC-TGAGG	30.1	23.1	47.9	RNA bulge	TCTGGCCATTGATGCCAAAAA	TCTGAATTTCCTGGAGAAAGGGT	No
RNF2-OT2	chr10:75832488	GcCATCTTAGTCATTcC-TGGGG	11.1	10.6	12.1	RNA bulge	GCCAGACCCGGGATTGTTT	GGCAGCAACTCTTTCACGGT	No
RNF2-OT3	chr2:177556598	GatATCTTAGcCATTACCT-AGG	6.0	4.9	22.3	RNA bulge	ACACGAGTTCATTGCTAACTCAG	CAGAGTGGTGTCCCAAGAAGTC	No
RNF2-OT4	chr18:24797589	GTCtaCTaAGTCATTAC-TGTGG	0.6			RNA bulge	TGTATGATTATCATGTGTCAATTAACTT	TTTCATGCCAAATACAATGTATA	No
RNF2-OT5	chr17:39300410	tTataacagcaagaaAaaTGAAC	0.2				Failed to be amplified		

Mismatched bases are in lower case. And deleted bases are indicated with dash. Inserted bases are in purple. Column is left blank when cleavage is not detected. ABE7.10 and gRNA expression vectors were cotransfected into 293T cells for validation.

Supplementary Table 5. EndoV-seq captured sites using HBB-28 (T>C) gRNA and ABE7.10

Site name	Position	DNA sequence at cleavage site	DNA cleavage score			Bulge	Deep seq primer		Validated
			**ABE7.10**	**BE3**	**Cas9**		**FP**	**RP**	**ABE7.10**
HBB-TA	chr11:5248323	GACTTCTATGCCCAGCCCTGG	N.D.	29.4	35.6		AAGAGCCAAGGACAGGTACG	ATGGTGTCTGTTTGAGGTTGC	No
HBB-OT1	chr3:144559579	GACTaCTATtCCCAGCCCTGG	18.6	29.7	133.5		TGATGCTGCTGAAGAGCCACT	AACTGTCCCTGTTTCAACCCAC	No
HBB-OT2	chrX:22104408	GACTaCcATaCCCAGCCCTGG	7.8		95.0		CCACTCTGATTTGGTGTCTCCT	CTGACCATCTTGGGTTCTTTGA	No
HBB-OT3	chr4:100539418	actacCTATGCCCAGCCCTGA	2.9	17.1	19.2		GAGGATGGGTGTAGGTTACATG	TCCTGCTAGAAAATAACAGACAAG	No
HBB-OT4	chr10:48743736	GACTaCcATcCCCAGCCCTGG	1.8		4.9		CCTGAAGAAAGAAATACCCTGAGA	CAATGTGGTGCTACTAAACAACCA	No
HBB-OT5	chr1:237449855	GACaaaaAgtaaagaatgGAT	1.7				Failed to be amplified		
HBB-OT6	chr4:3720348	GAaTTCTATaCCCAGCCCTGC	1.4		54.9		CAGGAGAAACATGGAGGAGGT	GTGGCTGGGAGAGAGTGGAG	No
HBB-OT7	chr5:15463671	GACTaCcATcCCCAGCCCTGG	1.1				ACCAGTGGAACAAACATTTTACAG	CTGTCCCTGCTTCTAGCCGA	No
HBB-OT8	chr10:72872815	GACTaCcATtCCCAGCCCTGG	1.1		45.1		ACTCTGACTTGGCATCTCCTTT	AATTAGTTTCCTCATTATTTTGGG	No
HBB-OT9	chr14:22056509	GACTaCcATcCCCAGCCCTGG	0.1		19.6		ACGAGTGCCTGAAAGGAGAAATA	GGTGTGGAACAAACCTCAACATG	No

Mismatched bases are in lower case. And deleted bases are indicated with dash. Inserted bases are in purple. Column is left blank when cleavage is not detected. ABE7.10 and gRNA expression vectors were cotransfected into 293T cells for validation. Note that the length of HBB -28 (T>C) gRNA is 18-nt.

Supplementary Table 6. EndoV-seq captured sites using FANCF gRNA and ABE7.10

Site name	Position	DNA sequence at cleavage site	DNA cleavage score of ABE7.10	Bulge	Deep seq primer		Validated
					FP	RP	ABE7.10
FANCF-TA	chr11:22647338	GGAATCCCTTCTGCAGCACCTGG	2.6		CGCCGTCTCCAAGGTGAAAG	GACCAAAGCGCCGATGGA	Yes
FANCF-OT1	chr11:47554037	GGAATCCCTTCTaCAGCAtCCTG	6.8		ACTCCAGTACAGGGGCTTTTG	ACACATTGGATTCACCTTTCCTC	No
FANCF-OT2	chr2:54853314	GGAATatCTTCTGCAGCcCCAGG	6.2		TCATCCGGCTCTGGGAATAC	TCTGTCGTCCTTCCTTTCGG	No
FANCF-OT3	chr20:60703092	GGAgTCCCccCTGCAGCACtcTGG	1.5	DNA bulge	TGACTACCACATACGGGAAACT	GTCACCCAAGCCCCTAATTTTG	No
FANCF-OT4	chr16:49671025	GGAgTCCCTcCTGCAGCACCTGA	1.1		CTTGTCGCAGCTCTCGCAC	AACTTTGAGAGCTTCCAGACCC	No
FANCF-OT5	chr4:76159966	ttAcTCaCTTCTGCAGGcACCTGG	1.0	DNA bulge	AGTCTCTTTTCTTAAGAGCCCAT	TGGTTTTATTCTAGCCTTTCCAGC	No
FANCF-OT6	chr1:75001257	tGgATCCCT-CTaCAGCACCTGG	0.4	RNA bulge	GAATCACAGAATGTAAGAAATGC	AATAGATTGAATTGTTCTAGTAGG	No
FANCF-OT7	chr4:159109759	GcAgaagtTTgTGtgtgtgtGTG	0.3		TGATACAGCCGAGTATGCCT	TCATCTCTGACTTGGGGACCTA	No
FANCF-OT8	chr16:22397909	ttttTCCaTTaTGtttCtaCTGA	0.1		CCTGGCCGACACACAGTTATTA	TCTAGTGATCCACCCGCCTC	No

Mismatched bases are in lower case. And deleted bases are indicated with dash. Inserted bases are in purple.ABE7.10 and gRNA expression vectors were cotransfected into 293T cells for validation.

Supplementary Table 7. EndoV-seq captured sites using EMX1 gRNA and ABE7.10

Site name	Position	DNA sequence at cleavage site	DNA cleavage score of ABE7.10	Bulge	Deep seq primer		Validated
					FP	RP	ABE7.10
EMX1-TA	chr2:73160981	GAGTCCGAGCAGAAGAAGAAGGG	0.3		CTCCGAGACGCAGGTGAAG	TTGCCCACCCTAGTCATTGG	No
EMX1-OT1	chr8:110129825	agtaCCaAGCAG-AGAAtAAAGG	32.6	RNA bulge	GACAGGGAGTGGGAGAATGTTG	TAAGCTTTGGAAGGAGATGTAGTG	No
EMX1-OT2	chr4:87256692	GAGTaaGAGaAGAAGAAGAAGGG	19.4		GAAATGGCGGACTAAAAATACG	TCAGGTATGTCTTTATAGCAGTGTGA	No
EMX1-OT3	chr8:96173267	GAGaaCcAagAGAAGAAGAAAGG	21.4		AAACCAGAGCTCTCAAGCTGT	AGAGCAAGACTCCATCTCAAAA	No
EMX1-OT4	chr14:31216722	agtaCCagagAGAAGAAGAgAGG	8.3		TAGCCCAGAGTCTCCCTGAATG	GCCTTTGGTAGTTTCCTCACAC	No
EMX1-OT5	chr7:3812781	GAGTCCtAGaAaAAGAAGAgAGG	6.6		ATACTTTCTGTTCTTTCTCCCTCTC	CAAAGGAAAATATTCTCAGGAATAG	No
EMX1-OT6	chr6:6263020	GAGTa-GAGtAGAAGAAGAgGGG	5.7	RNA bulge	GTCACAAAATGGCAATCACCTCAT	GAGTGGTTGGAATCCAGGGAAC	No
EMX1-OT7	chr2:15552105	GAaTCCaAGCAaA-GAAcAATGG	5.4	RNA bulge	TTTTGTCCCCCAGTTACCCCTA	GCCACATTTGTCTTTGAAACCATC	No
EMX1-OT8	chr1:172621530	GAGTaCcAGCAGAAGA-GAAGGA	5.4	RNA bulge	ATGTCAGTGGGTTTCCTTGCTG	TAGAGCTTGTAGGACCAGGGTT	No
EMX1-OT9	chr18:35137238	GAGTatcAGCAGAAGAA-AAAGG	4.3	RNA bulge	TCCCACAATATTTATGTGGATGTG	CAGTTCAGCAGCTGTTCCTAA	No
EMX1-OT10	chr18:1677040	GAGTCCaGAGCAaAAtAAGAAGGG	4.0	DNA bulge	CCTTGCGATCAGAAAGAGTGC	ATTCTGCATTTTATGCCCTGAAC	No
EMX1-OT11	chr4:38293023	GAGTatGAG-AaAAGAAGAACAG	2.8	RNA bulge	TTTCTTTATTCTCCTTGTTTAGG	AGTAGAGGGATTACAGAGCCTT	No
EMX1-OT12	chr15:22366612	GAGTa-GAGCAGAgGAAGAAGGG	2.7	RNA bulge	CAAGACATCAACACTCATTCACA	GCTTTTTTATTATTATGGGGACA	No
EMX1-OT13	chr4:131662222	GAaTCCaAG-AGAAGAAGAATGG	2.6	RNA bulge	TACCAATTTTCTGTTTGTCCCT	GCAGCATCAATGAATCTTGAG	No
EMX1-OT14	chr14:58383307	GAaTaCcAGC-GgAGAAGAAGGG	2.1	RNA bulge	GGGAAATTCTTAGTCATTATTGCTT	GAGCCTAGTACCCAATTCCAAA	No
EMX1-OT15	chrX:39754871	GAGTCaGAGCAG-AGAgGAATGG	1.6	RNA bulge	CACGGACACAGTCGGGAATA	TAGCTCAGAATGTCCTTTTCCTCC	No
EMX1-OT16	chr2:138279095	GAGTCCaAcaAGAAGAgaAAGGG	1.2		GTGCAGCACACCTTCATCTA	GACACTAACAACTTTATTCCCCTGT	No
EMX1-OT17	chr11:75983816	GAGTCC-AttAGAAGAAGAcAGG	1.2	RNA bulge	TCAATGGGCCCTAGACTGCC	TCGTGGAAGGTGAGGAATCAGA	No
EMX1-OT18	chr11:79484079	GAGTCCtA-aAGAAGAAGcAGGG	1.0	RNA bulge	ATGTTTGATTGCTCTCCGCTC	AGGGTCTAAAGTCCTCCCCTG	No
EMX1-OT19	chrX:71649612	GgactgGAGCAaAAGAAGAtTGG	0.8		CCTTGTGGGCTGCCAATTCT	CAGGCAACTGTGACAGGGAG	No
EMX1-OT20	chr3:45605387	GAGTCCacaCAGAAGAAGAAAGA	0.8		AATAGAAAATGGACTAAAACACTGC	ATAAAACTTTAAAAAAAGGAAATGT	No
EMX1-OT21	chr2:172374203	GAagtaGAGCAGAAGAAGAAGCG	0.8		TCCAGCTGCTCGTAGGACAT	TCTGGTGCATACAGAAGTCCAG	No
EMX1-OT22	chr17:30621475	ttGaCaGAGCAaAAGAAGAcAGG	0.6		AGGTTTGGCGCTACCTGACAT	AAATGAGTTTCAAGCAGCCATCC	No
EMX1-OT23	chr20:19258225	GAGTCtGAG-AaAAGAAGAATGA	0.5	RNA bulge	TGAGGACAAATGAACTGTGCTA	GATTCCAAAACCTGCAAACTTT	No
EMX1-OT24	chr5:14369519	GAGTaCaAGagAGAAGAAGAcTGG	0.5	DNA bulge	AGGGATACCAGTCGGCAGT	TTGCTGATCATGGGCGTCA	No
EMX1-OT25	chr11:62365273	GAaTCCaAGCAGAAGAAGAgAAGG	0.5	DNA bulge	CATCAACAGGGAGAGGGCAG	ATGGGCCTGTAGGAACCTGA	No
EMX1-OT26	chr8:109199399	GAGTCaGAGCAGAAGAA-AgAGG	0.5	RNA bulge	TTTACACAGTCATTGGTCCGC	TGGTTCTTGTTGCCTCTATCCT	No
EMX1-OT27	chr22:34716275	GtGaCaGAGCAaAAGAAGAAAGG	0.4		CTTTATCTCCGCAGCCACCTC	AAAGTCATGAGAAAACAGCTGAGAA	No
EMX1-OT28	chr2:9821543	agGTgGGAGCAGAAGAAGAAGGG	0.4		AGTTATTTCACCTCCTTTATTGCAG	AGGCAAATGCACACGTTCTG	No
EMX1-OT29	chrX:53467711	GAGTCCGgGaAGgAGAAGAAAGG	0.4		AGCAGGTAGGAATAAAGGCGA	TGCCTCTGACGACGAGCAA	No
EMX1-OT30	chr3:20706946	aAGaagGAaCAGAAGAAGAATGG	0.4		CTGTAGCCTTTCTTCTTTCCTT	CCCTTCAATATTATTTTTGGGA	No
EMX1-OT31	chrX:115538037	GAGTCCaAGC-cAAGAAaAATGG	0.4	RNA bulge	AATCAAGGTGTCAGGAGGGC	TTTTCAAAAGTGGAAGAGGGAG	No
EMX1-OT32	chr10:81985204	GAGaagGAGaAGgAGAAGAAGAA	0.2		Failed to be amplified		
EMX1-OT33	chr6:82349278	tctctCtgtgtGtgtgtGtgTGT	0.1		Failed to be amplified		
EMX1-OT34	chr19:22423670	GAaTCCatGCAGAAGAAGAgAGT	0.1		CCATCTCAAAAAAAGAGAGAAAAG	CCTACAGGGGACATTATGAAATAT	No

Mismatched bases are in lower case. And deleted bases are indicated with dash. Inserted bases are in purple.ABE7.10 and gRNA expression vectors were cotransfected into 293T cells for validation.

Supplementary Table 8. EndoV-seq captured sites using Dmd gRNA and ABE7.10

Site name	Position	DNA sequence at cleavage site	DNA cleavage score			Bulge	Deep seq primer		Validated
			ABE7.10	BE3	Cas9		FP	RP	ABE7.10
Dmd-TA	chrX:85107580	TAATAGGGGACGAACAGGGAGG	10.4		149.7		CCCTCTTGCCCCTTAACTAGG	CTGTACTTGTCTTCCAAGTGTGC	Yes
Dmd-OT1	chr5:68074548	gAATAGGGGAaGAACtGGaGAG	26.2				CTCACTCCCATTCTTACCTCTC	TGTTGTACTTCTATGCACACACA	No
Dmd-OT2	chr2:38630648	TAATA-GaGAaGAACAGGaAGG	20.6		62.0	RNA bulge	TGTTATCACGTGCAGTCTACCC	CTTAGCCTGTTGCCTTGTGG	No
Dmd-OT3	chr3:18685277	TAAcAGGGGA-GtACAaGGTGG	18.9	10.0	43.6	RNA bulge	GAAAAGCCCGTCCATCCAAC	CTTATCTAGGCATCCCAATTAGCA	No
Dmd-OT4	chr8:109357064	ggATAGGGGAaGAACAGGcTGG	14.9		32.6		TGACCTCATCATCTGCTTCGTC	CTCATCATTTTAGGAAGGCAGCTA	No
Dmd-OT5	chr6:21107031	TAATAGaG-ACaAACAGaGCAG	10.3			RNA bulge	CGTTCTGTGTTCTGGCTCAAAG	TTCATTTCATGGACATTTTCTGGTG	No
Dmd-OT6	chr3:7712276	TAATAGGGGA-GAACA-GGaGT	9.8			RNA bulge	TCCCCACTTCCCGAGGTT	CTGAAGGGCCCAAGTGACG	No
Dmd-OT7	chr15:56278377	TAATAaGGGA-GAACAGtGAGG	9.7		35.7	RNA bulge	AACGATTAAGTGGCTGAACTTGC	CCCGAACCTTTACATCTACCCAT	No
Dmd-OT8	chr1:50953768	TgATAGGGGAaGAACAaaGAGG	9.0				AGACAGAAAGAGAGAGGAGGCT	CACCCAACAGTTACCTAGCAAC	No
Dmd-OT9	chrX:44557778	gtgTAGGGGAaGAgCAGGGAGG	7.1				AATCCTCCCACGTCTGCC	ATTTTATTCAAATGCTTTCTCAGC	No
Dmd-OT10	chr9:28811460	gtAaAGGGGAgGAACAtGGGGG	6.8		37.5		GTTTCAGAATGAAGGTGGAATAGTT	CCATTTCAACTCTTTTCCTCTATGT	No
Dmd-OT11	chr10:19308177	TtggAGtGtgtGtgtgtGtGTG	5.1				Failed to be amplified		
Dmd-OT12	chr4:113100247	gtgTAGGGGAaGAgCAGGGAGG	3.9				CAGTCTTCTCGTGTCCACCC	GGCCATGAGGTAAATGCAACA	No
Dmd-OT13	chr11:76386116	cAATAGGGGAtGAACAGaaGTG	3.4				TGCAAGAGCTGGGGAGATTG	TGTTTGTACTCAGTGTACGTGGT	No
Dmd-OT14	chr4:154691742	gAATgGGtGgacAgAtGGaTGG	3.2				Failed to be amplified		
Dmd-OT15	chr3:118742145	gtATAGGGGACttACAGaGAGG	3.0				ATGAAATTAGCTCCTTTTTTATTAG	GAGAGTCTCTTGGTCTTGTGAAC	No
Dmd-OT16	chr18:38382219	aAgaAaGaGAgaAACAGaGAGA	2.8				TGCCCTCTTCTGGTGTATCT	CCTTTGCTTTATGAAGGAGCC	No
Dmd-OT17	chr14:119571143	ggATAGGGGACtAACAGaGGGG	2.2				CATTTCAAATGTTATTCCCCT	TTGGTCCTGTGGAGGCTC	No
Dmd-OT18	chr17:94115973	gAATAGGGGAaGAAgAGGGGAG	2.2		54.4		CACCCTTCTGTTTTTGTTGATTCTA	TGAGAAGGCCTTGTATCTCTCTA	No
Dmd-OT19	chr7:144589742	gtgatGGGGAtGAACAGGGTGA	2.1		27.1		AAGGATGCTGACTTGGCTCC	GCACAAGGCCATTATGCAGG	No
Dmd-OT20	chr7:129549035	gtAatGGGGAaGAACAGGaTAG	2.0		52.2		CATCAACAGCCTGTCAAAATGT	TGGTTACATTATAACAACAGGGAC	No
Dmd-OT21	chr10:105155697	agAgAGGcagaGAcaAGaGAGA	1.8	1.0			Failed to be amplified		
Dmd-OT22	chr5:25564921	gtAataGGGACagACAGGGAAG	1.7		22.9		CATTCTCCAAATCCAATACCCC	AGGCTGCTGAGAGATTCCATAG	No
Dmd-OT23	chr1:114713080	TcATAGGaGAtGAACAGGGTGG	1.7		38.7		CTCACTGACTCTCTCCTGGTGA	CACTTCTATGTGGGATATGTTCTA	No
Dmd-OT24	chr9:113229999	gtgTAtGtGtattgCAtGtGTG	1.6				Failed to be amplified		
Dmd-OT25	chr17:48683821	TAATAGGGcA-GAACAGaGGGG	1.5		87.0	RNA bulge	CAGCCATGGAAAGTACCAGAGTA	TAACCTTAGAGATGGGGGAGGC	No
Dmd-OT26	chrX:75689900	TtggAcattcaaActgccaAGT	1.5				Failed to be amplified		
Dmd-OT27	chr7:64084969	cAtTgtGacACcAcaAGGGAGC	1.5				GACATCACAGTTCTCCGCTG	GCACTGTTACACCCAATGGA	No
Dmd-OT28	chr6:70661921	gAATAaaGGAgGAACAGGGAGG	1.3				TCCCTTCTCAGAATCTTCTCTTCC	GTCACAATGATGGATTTCCTTGC	No
Dmd-OT29	chr4:35623375	atATAGGGGAatAACAGGGTGG	1.1		62.4		GAGGATGGCCTTGTGCAT	TTCTCCTTTCCAAGTCTCCC	No
Dmd-OT30	chr4:105750585	gtATAGaGGAtGAAC-GGGAGG	1.1			RNA bulge	GTACGGAGTAGAGGGTCAACTA	AGAGTCACAGTTCAGATCCACC	No
Dmd-OT31	chr10:60144734	cAcacacacACacACAGacACA	1.0				Failed to be amplified		
Dmd-OT32	chr18:69242933	TAATAaGGGAaGAACAtGaGGG	1.0		24.7		TCGATGGCTATGCGCTCTAGT	ATCATTGTGTTCAGGGTCTCCAA	No
Dmd-OT33	chr14:67559380	TAATAGtt-ACaAACAGGGTGA	1.0			RNA bulge	GTCCTGATGGTTCAGGATCTTCC	TCTCCACAGGTCAAGGTGGC	No
Dmd-OT34	chr9:115194975	gAgagaGaGAgagAgAGaGAGA	1.0	1.3			Failed to be amplified		
Dmd-OT35	chr9:63897192	TAAgAGGGGACaAACA-tGGGG	1.0		15.0	RNA bulge	Failed to be amplified		
Dmd-OT36	chr5:122999874	acAatatttggaAtgAactACA	1.0				Failed to be amplified		
Dmd-OT37	chrX:15755397	aAtctGGGGAaGAACAGGGGTG	0.9						
Dmd-OT38	chr2:86793703	TAATAGGGcA-GAACAGaGGGG	0.9		100.1	RNA bulge			
Dmd-OT39	chr9:36441999	atATAGaGGACGAACAGGaTGA	0.8						
Dmd-OT40	chr2:93155713	ggggAGGGGAgGAgagGGGAGG	0.8						
Dmd-OT41	chr5:30265308	gAAgAGGGGAaGAAaAGGGTGG	0.8						
Dmd-OT42	chr2:121399128	aAATAGGtGA-GAACAGGGAGG	0.8			RNA bulge			
Dmd-OT43	chr11:11833290	gAATAGGGaAaGAACAGGcCGG	0.8						
Dmd-OT44	chr14:68672898	gAAgAGGGGAtGAAaAGGGAGG	0.7						
Dmd-OT45	chr2:107810930	gAATAGaGaAaGAACAGtGAGG	0.7						
Dmd-OT46	chr1:100466391	TAtTAGGGGAaGAA-AGGaAGG	0.7			RNA bulge			
Dmd-OT47	chr2:101548761	agAgAGaaGAaGAAgAaGGAGG	0.7						
Dmd-OT48	chr18:16493349	ctAgtaaaaAaatAaAaaaAAA	0.7						
Dmd-OT49	chr4:63795864	TtATAGGGGAtGAA-AGGtAGG	0.6		39.8	RNA bulge			
Dmd-OT50	chr1:72514314	gtAatatGGgatAACAtGGTGG	0.6		25.8				
Dmd-OT51	chr1:193706728	aAATAGGGGAtG-ACAGaGGGG	0.5			RNA bulge			
Dmd-OT52	chr8:11449501	atAccacacAtatACAacaCAC	0.5						
Dmd-OT53	chr3:38034017	gAAgAGGGGAaGAACtGGGAGG	0.5						
Dmd-OT54	chr15:96524134	gAAgAGGaGggGgAgAtGGAAG	0.5						
Dmd-OT55	chr6:110656172	aAATAGaGGAtGAgCAGGGTGG	0.5		23.5				
Dmd-OT56	chr2:142550445	gAgataaatgttAcaAataTAT	0.5						
Dmd-OT57	chr11:80045519	gAAgAGGaGAgGgAgAGGGAGA	0.4						
Dmd-OT58	chr7:15654203	aAgTtccaGggagtCctGGAAC	0.4						
Dmd-OT59	chr18:47959047	gAATAGGaGAtaAACAGGaTGG	0.4		19.0				
Dmd-OT60	chr4:150750392	gAtagatacAgGAAgagGGGAGG	0.4			DNA bulge			
Dmd-OT61	chr2:146577012	TAAaAGGGG-tGAgCAGGGAGG	0.4			RNA bulge			
Dmd-OT62	chr8:23640273	aAggAaGGaAgGAAggAaGGAGG	0.4			DNA bulge			
Dmd-OT63	chr10:32029870	atgTAGGGGAgGAACAGGGAGG	0.3						
Dmd-OT64	chr1:28001408	gtATAGGGaAaGAAgAGGGAGG	0.3		26.1				
Dmd-OT65	chr4:152964295	gggcAtGtGtgtgtgtGtGTGT	0.3						
Dmd-OT66	chr2:121124960	gggaAGGGGA-GAACAGGaAGG	0.3		31.5	RNA bulge			
Dmd-OT67	chr12:99562813	gttgcattGAgGAgaAaaaAAA	0.3						
Dmd-OT68	chr12:65206823	gAgagaGaGAgagAgAGaGAGG	0.3						
Dmd-OT69	chrX:11959147	gggTAGGGGAC-AACAGGaAGG	0.3		27.4	RNA bulge			
Dmd-OT70	chr11:18138193	attgttGaGctcgAggGaGGAA	0.3						
Dmd-OT71	chr17:70450249	aAcTAGttGAgagAgAGGGAGG	0.3						
Dmd-OT72	chr4:8423230	TAATAGttaACGAAaAGGGGGG	0.2						
Dmd-OT73	chr4:69130277	gggTAGGGGA-GAAaAGGGGGG	0.2		9.5	RNA bulge			
Dmd-OT74	chr2:39910896	gAAgAGGaGAgGAggAGaGGGG	0.2						
Dmd-OT75	chr7:3723287	gAAgAaGaaGaagAagaGGAGG	0.2						
Dmd-OT76	chr19:21565132	gAAagGaaaggagAggaaaGGA	0.2						
Dmd-OT77	chr15:91368329	gAgagGaGGAaagggAGGaAGG	0.2						
Dmd-OT78	chr11:102822533	gtcTAcaGaggGAgCttcaGGA	0.1						
Dmd-OT79	chr4:127557505	gtcTcaaaagaGAAagaaaGAA	0.1						
Dmd-OT80	chr6:118582311	aAAggGGaGggGAggAaGGAAG	0.1						

Mismatched bases are in lower case. And deleted bases are indicated with dash. Inserted bases are in purple. Column is left blank when cleavage is not detected. ABE7.10 and gRNA expression vectors were cotransfected into 293T cells for validation. Note that the length of Dmd gRNA is 19-nt.

Supplementary Table 9. Digenome-seq captured sites using HBG gRNA and BE3

Site name	Position	DNA sequence at cleavage site	DNA cleavage score			Bulge	Deep seq primer		Validated
			BE3	ABE7.10	Cas9		FP	RP	ABE7.10
HBG1-TA	chr11:5271278	GTGGGGAAGGGGCCCCCAAGAGG		2.0	42.3		TCCTGGTATCCTCTATGATGGGA	GTGGAGTTTAGCCAGGGACC	No
HBG2-TA	chr11:5276202	GTGGGGAAGGGGCCCCCAAGAGG		1.4	42.4		TGGTGGGAGAAGAAAACTAGC	GTGGAGTTTAGCCAGGGACC	Yes

Mismatched bases are in lower case. Column is left blank when cleavage is not detected. ABE7.10 and gRNA expression vectors were cotransfected into 293T cells for validation.

Supplementary Table 10. Digenome-seq captured sites using VEGFA3 gRNA and BE3

Site name	Position	DNA sequence at cleavage site	DNA cleavage score			Bulge	Deep seq primer		Validated
			BE3	ABE7.10	Cas9		FP	RP	ABE7.10
VEGFA3-TA	chr9:110103695	GGTGAGTGAGTGTGTGCGTGTGG		18.1	214.5		GTGCAGACGGCAGTCACTAGG	CTATTGGAATCCTGGAGTGACCC	Yes
VEGFA3-BE-OT	chr2:230506241	GGTGAGcaAGTGTGTGtGTGTGG	9.3	9.2			CAGATGCAAATGCAAAAGAACAATA	AATGGATCAGAGGACCAACATTGTA	Yes
VEGFA3-BE-OT	chr2:73317050	GGTGAGTcAGTGTGTGaGTGAGG	6.9				GCCAGGAACACAGGAATGCTA	TGAGCGCTCTTCGTCTTTCC	No

Mismatched bases are in lower case. And deleted bases are indicated with dash. Inserted bases are in purple. Column is left blank when cleavage is not detected. ABE7.10 and gRNA expression vectors were cotransfected into 293T cells for validation.

Supplementary Table 11. Digenome-seq captured sites using HEK293-2 gRNA and BE3

Site name	Position	DNA sequence at cleavage site	DNA cleavage score			Bulge	Deep seq primer		Validated
			BE3	ABE7.10	Cas9		FP	RP	ABE7.10
HEK293-2-TA	chr5:87240603	GAACACAAAGCATAGACTGCGGG	27.4	19.4	131.7		ACAATGATAACAAGACCTGGCTG	CCCCATCTGTCAAACTGTGCG	Yes
HEK293-2-BE-OT1	chr15:93557679	GAACACA-tGCATAGACTGCTAG	37.0	35.3	126.9	RNA bulge	CTCTGAGAGTGCCGCCAG	ACTGGCGTTTACTACCTCCT	No
HEK293-2-BE-OT2	chr4:90522173	GAACACAAtGCATAGAtTGCCGG	33.6		82.1		GTTCAAAAACAAACAGAGAAGAC	ATACATATTTAATGCTCCCACAC	No
HEK293-2-BE-OT3	chr1:185056773	cAtCttAgt-CATtacCTG-AGG	31.2			RNA bulge	AACGGAACTCAACCATTAAGCA	CCAACATACAGAAGTCAGGAATGC	No
HEK293-2-BE-OT4	chr2:19844956	aActcCAAAGCATAtACTGCTGG	28.5		65.9		ATCAAAGGAAAAGCAACGTGAGC	ATGTGCAAGATACTTTGGGAAACT	No
HEK293-2-BE-OT5	chr5:7625827	GtACACA-AtaATAGACTGCAGG	25.2	21.7	38.4	RNA bulge	TTGGTGTGAGGGCAGTTTGTAT	TAGGAAAAGAACACTTCCGGGT	No
HEK293-2-BE-OT6	chr18:22360702	GAAt-CAAAGCAcAGACTGCAGG	19.5		80.4	RNA bulge	CACACTGAGTGGCAAGCCT	AAGCCACCCGTTTTAGTGGT	No
HEK293-2-BE-OT7	chr1:67142248	GAAC-CActGCATAGAaTGCAGG	8.0		21.8	RNA bulge	GGTGCCTAAGCACTCATTTTTCT	TGTTTTGCTGTAAGTTGGTTGATT	No
HEK293-2-BE-OT8	chr2:192248354	GAACAC-AtaCATAGACaGCTGG	1.6		20.4	RNA bulge	AAGGAACTGCAGAGTTCTAGAGA	AAGGTCATAAAAGGGTGTTGTG	No

Mismatched bases are in lower case. And deleted bases are indicated with dash. Inserted bases are in purple. Column is left blank when cleavage is not detected. ABE7.10 and gRNA expression vectors were cotransfected into 293T cells for validation.

Supplementary Table 12. Digenome-seq captured sites using RNF2 gRNA and BE3

Site name	Position	DNA sequence at cleavage site	DNA cleavage score			Bulge	Deep seq primer		Validated
			BE3	ABE7.10	Cas9		FP	RP	ABE7.10
RNF2-TA	chr1:185056773	GTCATCTTAGTCATTACCTGAGG	19.3	12.1	160.1		AACGGAACTCAACCATTAAGCA	CCAACATACAGAAGTCAGGAATGC	Yes
RNF2-BE-OT1	chr17:53928588	GTCATCTTAGTCATTAC-TGAGG	23.1	30.1	47.9	RNA bulge	TCTGGCCATTGATGCCAAAAA	ACTCTCTGAAACTGTCTGTGAAA	No
RNF2-BE-OT2	chr2:73160987	GagtcCgagcagAagAagaaGGG	15.2				CCGGAGGACAAAGTACAAACG	CCCACCCTAGTCATTGGAGGT	No
RNF2-BE-OT3	chr4:131662222	agaAgaaTgGTaATTACCaGAGG	11.9				TACCAATTTTCTGTTTGTCCCT	AGCAGCATCAATGAATCTTGAG	No
RNF2-BE-OT4	chr10:75832488	GcCATCTTAGTCATTcC-TGGGG	10.6	11.1	12.1	RNA bulge	GCCAGACCCGGGATTGTTT	GGCAGCAACTCTTTCACGGT	No
RNF2-BE-OT5	chr2:177556598	GatATCTTAGcCATTACCT-AGG	4.9	6.0	22.3	RNA bulge	CACGAGTTCATTGCTAACTCAGG	CAGAGTGGTGTCCCAAGAAGT	No
RNF2-BE-OT6	chr5:92036966	GgtATCTaAGTCATTACCTGTGG	2.2				ATTTTAAGCTAGAATGTGTTTGTTG	AAATCTATTTGGTCTGTAATTTTCA	No
RNF2-BE-OT7	chr3:69832733	GTC--CTTAGTCATTAtCTGTGG	1.5			RNA bulge	Failed to be amplified		
RNF2-BE-OT8	chr1:81660341	GggtTCacAGgtgggACCTGAGG	1.1				ACAATGTATTTTTCTTCCCACCAT	AGTCTTGAAGTTTTCCCTCCAAAA	No
RNF2-BE-OT9	chr6:91669508	aaaATtTctcaaATTtgaTGAGA	0.6				Failied to be amplified		

Mismatched bases are in lower case. And deleted bases are indicated with dash. Inserted bases are in purple. Column is left blank when cleavage is not detected.

ABE7.10 and gRNA expression vectors were cotransfected into 293T cells for validation.

Supplementary Table 13. Digenome-seq captured sites using HBB-28 (T>C) gRNA and BE3

Site name	Position	DNA sequence at cleavage site	DNA cleavage score			Bulge	Deep seq primer		Validated
			BE3	ABE7.10	Cas9		FP	RP	ABE7.10
HBB-TA	chr11:5248323	GACTTCTATGCCCAGCCCTGG	29.4		35.6		AAGAGCCAAGGACAGGTAC	ATGGTGTCTGTTTGAGGTTGC	No
HBB-BE-OT1	chr19:47303590	GACTcCTAatCCCAGCCCTGG	40.1		121.1		TCTGCTGAGTCCACCAGTCT	CTTGGGACACAGTGGAGGTG	No
HBB-BE-OT2	chr2:179291342	GACTTCaATcCCCAGCCCTGG	35.8		146.2		GCTGGTGCTGCTGAGTAACC	GGAAAGAGACCTGTCCCTGA	No
HBB-BE-OT3	chr6:138466817	GACTTCTATaCCCAGgCaCAG	34.7				CACCCAAGTTCCTACAGGTC	TTTAGTGGTTGAGAGAAATGCGA	No
HBB-BE-OT4	chr1:30284224	GACTTCTATaCCCAGCaCTGG	31.0		270.9		AGTAGAGTATCGCTGAGCGT	GCCCTCTCACCTAATAGGGTTC	No
HBB-BE-OT5	chr7:134091170	GACTcC-ATGCCCAGCCtCAG	30.5		115.9	RNA bulge	ATGGCCTCACCAAAGGACTG	TTGTCAGACACACAGAGCCC	No
HBB-BE-OT6	chr17:3560216	GtCTTCTATGCCCAGCaCAGG	30.2		138.9		GCAGAGCCTGGGAAAGGAA	GAGCTGTCTGTGCTCCTCTG	No
HBB-BE-OT7	chr3:144559579	GACTaCTATtCCCAGCCCTGG	29.7	18.6	133.5		TCTGATTTGGTGTCTCCTTTG	AGCTTCCCTCATTTTGGGTT	No
HBB-BE-OT8	chr4:82712791	GcCTTCTAcGCCCAGCCCGGG	22.8				TCACATGTGCGGTTACAGGT	GTTTCAAGTCTGATTGCCTGGT	No
HBB-BE-OT9	chr10:103514927	actccCTATGCCCAGCtCAGG	22.3				TGCGGATGGAGGTAAGTCTG	GGGCTCCTTCTGCCATCATT	No
HBB-BE-OT10	chr22:42797028	GtCTTCcATGCCCAGCCCAGG	21.9		210.4		GGAAAGCAAGGTCCCTGGA	CACTTCTCTGGCAACCCCAT	No
HBB-BE-OT11	chr21:34843979	GACTTC-AaGCCCAGCCCAGG	21.6		48.5	RNA bulge	TGTGATGGCTTGCACCTGTA	GCCCCTAGAATTTTCATTTGA	No
HBB-BE-OT12	chr11:2712612	GgCTTCTATGCCCAGaCCCAG	21.1		136.1		AAAAGGCGATGTCTCACCCC	ATCAGAGACTCAGTGGGGGC	No
HBB-BE-OT13	chr7:143977721	GACTcCTATt-CCAGCCCTGG	20.3		8.4	RNA bulge	ATGGAGCCTGTGAAAGCACT	ACAGTGGGAGATGACACGAG	No
HBB-BE-OT14	chr12:131308600	GACTcCTAacCCaAGCCCCGG	19.6				CACAGATATTTCTGGGTTTG	CCTGGCCTGCATGATTCTTT	No
HBB-BE-OT15	chr1:44748946	atacTCTATaCCCAGCCCTGG	18.3		11.4		ACATGCCTAGCTCTGCCTTG	CAGACATGGCCATCTGGATCA	No
HBB-BE-OT16	chr22:39661111	GgCTTCTATGCCCAGCaCGGG	17.6		168.8		TGGAAAGCTATTCAGGTCCA	GTCACGTCGCAGATTAGTGC	No
HBB-BE-OT17	chr4:100539418	actacCTATGCCCAGCCCTGA	17.1	2.9	19.2		GAGGATGGGTGTAGGTTACA	GATTTAAAACTCCCAGTGGAGTG	No
HBB-BE-OT18	chr3:124696144	GACTaaTATaCCCAGCCtCAG	16.9		63.1		ATGATTATGAGGCTTCCCCA	AGATGGCCAATAAGCACATTG	No
HBB-BE-OT19	chr5:156606363	atCTTCcATG-CCAGCCCTGG	16.7			RNA bulge	AGGTTTGCTGGTAGTGACAG	TGAATGTTGAGGACAATGGAGT	No
HBB-BE-OT20	chr7:138378071	tctcTCTATaCCCAGCCCAAG	15.7				GTGGTTCACGCCTGTCATCT	TCCTGCCTCAGCCCTCTAAGT	No
HBB-BE-OT21	chr9:83390160	atCTTCTATtCCCAGCCCAGG	15.4		37.9		AGCATTTCGTATCTTCTATGC	TGCCTTCTTTCCCAAGGTCA	No
HBB-BE-OT22	chr11:74989539	agCTTCTATcCCCAGCCCAAG	13.1		23.4		TCCAAATCCCCCATGAGAGG	GCCCTTGCACCTAGAAGCCT	No
HBB-BE-OT23	chr1:165064106	GACTTtcATaCCCAGCCCTGG	11.2		79.9		CGTAACTGCCTGAAGGAAGA	CCCAGAGGACCTGTGAAATGT	No
HBB-BE-OT24	chr22:49068356	tAaTTCTATaCCCAGCCaCGG	10.5		9.5		GTGAGATTGAAGAAGTTGCC	GTGATGAGCAAAGGAGTGAGGT	No
HBB-BE-OT25	chr15:70395323	GtCTTCTATc-CCAGCCCTGG	10.1		44.6	RNA bulge	CTGGGTGATTCAACTCCTCC	AGAGTCCAGAGGAGAGGAGC	No
HBB-BE-OT26	chr11:58272825	tcttTCTATGCCCAGCCCAAG	9.4		17.7		ACATGCCCAGATACACATCT	CTCCTCATCCTGCTCCTCCT	No
HBB-BE-OT27	chr2:203568291	GACTTCTAga-CCAGCCCGGG	8.8		40.9	RNA bulge	TGCCCAGCTAGTTTTTGAG	TTCACCCATTAAGAGAGATTAAG	No
HBB-BE-OT28	chr8:10594898	aACTTCaATGCCCAGCtCCGG	4.7		101.7		TGAGACGGAGAATGTTGAG	TCAAACCCTAAAAATTCGAGTTC	No
HBB-BE-OT29	chr1:24385837	GACTTC-AaGCCCAGCCtGGG	3.8		6.8	RNA bulge	CGCTCAGCCTCCTGAGCA	TTCTAGAGATTTCCATCCAA	No
HBB-BE-OT30	chr13:51623981	tctcCTAaaCcCCAGCCCTGG	3.2				CTAGGAGAGCTGTGATGACA	CACAAATCTTGCCCAGTACCTC	No
HBB-BE-OT31	chr1:192903250	attTTCTATGCCCAGCCaAGG	3.0		8.7		ACAAGCACAGGGCAGGATT	AGAGATGGAGTGGAGGTGGG	No

Mismatched bases are in lower case. And deleted bases are indicated with dash. Inserted bases are in purple. Column is left blank when cleavage is not detected.

ABE7.10 and gRNA expression vectors were cotransfected into 293T cells for validation. Note that the length of HBB -28 (T>C) gRNA is 18-nt.

Supplementary Table 14. Digenome-seq captured sites using Dmd gRNA and BE3

Site name	Position	DNA sequence at cleavage site	DNA cleavage score			Bulge	Deep seq primer		ABE7.10
			BE3	ABE7.10	Cas9		FP	RP	Validated
Dmd-TA	chrX:85107580	TAATAGGGGACGAACAGGGAGG	0.4				CCCTCTTGCCCCTTAACTAGG	CTGTACTTGTCTTCCAAGTGTGC	Yes
Dmd-BE-OT1	chr14:55699331	TAAcAGGGGAC-AACAaGGTGG	16.3		87.6	RNA bulge	ACCATTGAGTCCTGGAGATTG	TTGAAGCCATTGCACTAGAAT	No
Dmd-BE-OT2	chr7:56357792	TAAcAGGGGAC-AACAGGGAGG	13.2		50.1	RNA bulge	GTTGTGCCTTCCTCAAACAGAG	TGCAAGAGTCAAAAAGGCCATTAC	No
Dmd-BE-OT3	chr3:18685277	TAAcAGGGGA-GtACAaGGTGG	10.0	18.9	43.6	RNA bulge	AAAGCCCGTCCATCCAACAT	GTCTTATCTAGGCATCCCAATTAG	No
Dmd-BE-OT4	chr1:171058132	gtActGGGcAgtggtgGccACG	2.6				AATCTAGCAATGGGAAGGGTGGA	GCCCACAGTGGTCTTGAATTGTTAT	No
Dmd-BE-OT5	chr18:33660766	acATActacACacACAcctCAC	2.5				CCGTGTCTCTGTTTTCTGCATAT	TGTGTGGATGGTATGTATTGTATGT	No
Dmd-BE-OT6	chr2:103713896	TAAcTAaGGGACGAgACAGGGAGG	2.4			DNA bulge	GGCAAGGAAGGACTCGGATT	TCACCTGGACTTTGGGCTTC	No
Dmd-BE-OT7	chr19:23408787	agAgAGaGGAgGggCAaGcAGC	2.0				Failed to be amplified		
Dmd-BE-OT8	chr6:131394692	TAAaAaGaatgaAAgAGGGAAT	1.9				GTCTACAGAGTGAGTTCCAGGA	TCTTAAAGCAGAATTCAGAGTTAA	No
Dmd-BE-OT9	chr6:58526528	cctgAcaGttgaAgagGccCAG	1.9				Failed to be amplified		
Dmd-BE-OT10	chr17:31275942	TgtgtatGtgtGgtgtGtGTGT	1.7				Failed to be amplified		
Dmd-BE-OT11	chr9:115194975	gAgagaGaGAgagAgAGaGAGA	1.3	1.0			AGAAACCCTGTCTTGAAAAGAG	CCAACACCTGTGCCTGTAAC	No
Dmd-BE-OT12	chr16:80113549	agggAtaGaggGAAaAGGaGAG	1.3				Failed to be amplified		
Dmd-BE-OT13	chr17:45510769	TctgtGtGtAtatctAtctGTC	1.2				TGTGTGTGCATCTGTCTGTCT	TCTTCTGGTCTCTGTGGGTAT	No
Dmd-BE-OT14	chr3:128032593	gAgggGGGGAgGAAgAGGaAGA	1.1				Failed to be amplified		
Dmd-BE-OT15	chr3:137629650	cAcTAccacACacACAccaCCA	1.1				Failed to be amplified		
Dmd-BE-OT16	chr10:105155697	agAgAGGcagaGAcaAGaGAGA	1.0	1.8			AGGAGGGAGAAGGACAAAGGG	GCTCAAAATCTTGCCATGGTT	No
Dmd-BE-OT17	chr6:109872156	TgtatatatAtatAttcttATG	0.9				TCAAAACAAGACTCACCCAAGTT	AGACGACTGAGTTCACAGCCTA	No
Dmd-BE-OT18	chr11:34530433	ccATcGaaagaaAgagaGGGAG	0.5				TGAGAGCAGACACCTTGTAAGTC	GGAGCCCTCACCTTTTGGAA	No

Mismatched bases are in lower case. And deleted bases are indicated with dash. Inserted bases are in purple. Column is left blank when cleavage is not detected.

ABE7.10 and gRNA expression vectors were cotransfected into 293T cells for validation. Note that the length of Dmd gRNA is 19-nt.

Supplementary Table 15. Digenome-seq captured sites using HBG gRNA and Cas9

Site name	Position	DNA sequence at cleavage site	DNA cleavage score			Bulge	Deep seq primer		Validated
			Cas9	ABE7.10	BE3		FP	RP	ABE7.10
HBG1-TA	chr11:5271278	GTGGGGAAGGGGCCCCCAAGAGG	42.3	2.0			TCCTGGTATCCTCTATGATGGGA	GTGGAGTTTAGCCAGGGACC	No
HBG2-TA	chr11:5276202	GTGGGGAAGGGGCCCCCAAGAGG	42.4	1.4			TGGTGGGAGAAGAAAACTAGC	GTGGAGTTTAGCCAGGGACC	Yes
HBG-Cas9-OT1	chr22:43265597	GgGGaGAAGGGaaCCCCAAGGGG	100.6				CATGTGGAAGCCCAGGTCAA	TCCCTGGGGCTTACTAGCTT	No
HBG-Cas9-OT2	chr19:33399599	catGGGAAGGGGCtCCCtAGTGG	81.1				GAAGCTGAGGACACCTTGGA	GACATTCCCCCGGTGTGAG	No
HBG-Cas9-OT3	chr17:27911000	GgGaGGAAGGGatCCCCAAGGGG	76.4				TCCCAGGAAACGAGGTCTCT	AGGCTCCAGCATGAAAGGAC	No
HBG-Cas9-OT4	chr1:76035985	agaaGGAAGGGGCCCaCAAGTGG	63.0				GCAATGACAGGATGTAAGTAACT	GTTTAGCCTTTCTCTTTGTGTA	No
HBG-Cas9-OT5	chr9:104713616	catGGGAAGGGGCCCaCAAGTGG	61.4				GCCTGCGATCTTGGTCTCATA	CCACCAGGTAGTTGTTCGTCA	No
HBG-Cas9-OT6	chr9:36788070	agGGGGAAGtGGCtCCCAAGTGG	54.9				TTCACACTGCCTTTGTCCCC	AATGCCCCTCTCCTTTTCAGG	No
HBG-Cas9-OT7	chrX:12298826	agtGGGAAaGGaCCCCCAAtGAG	54.1	0.7			CCCATGAAGTCCCCACTGTC	CAGGTGGCTAGGCTGAAACA	No
HBG-Cas9-OT8	chr4:42658707	cgGGGGgAaGGGtCCCCtAGGGG	50.3				CTGGAAAGGGTCTGCGAGTACA	TCGCGCGCCGAAGGTAA	No
HBG-Cas9-OT9	chr11:33058597	tgtGGGAAtGGGtCCCCAAGAAG	44.9				TGTTGATTATCTTTTGCTGTGTG	CAAAGTTAGTCTCATTAGCCT	No
HBG-Cas9-OT10	chr3:23491400	taGGGGgAGGGGtCCCCAtGTGG	44.2				TTCACTGGCAGAAACCCCAG	GTACTCACGTGGCAGGGTAG	No
HBG-Cas9-OT11	chr9:21122163	GgtGaGAAGGaGCCCaCAAGTGG	43.9	1.0			TTGTTATAGAGGAACCCCAGCC	TTTGCTTAACATACAGAGTTC	No
HBG-Cas9-OT12	chr10:52240792	cctGGGAAGGaGCCCCCAtGGGG	41.1				TGAGACCCAGATCCATCCCTT	TCCATCCAGACTGCCAACAAT	No
HBG-Cas9-OT13	chr3:34824512	GgtaGGAAGGGGCtCCCAAGAGG	39.4	0.7			TTCCCTTACTGATCCGTGTCC	AAACACTCTGTGAGTCACTTT	No
HBG-Cas9-OT14	chr17:80534192	tcGGGGAAGGacCCCCCAAGTGG	38.4				Failed to be amplified		
HBG-Cas9-OT15	chr8:129326161	GgtGGGAAaGGGgCCCCAtGGGG	32.5				CATTTCCCTCCCTGCTCTCA	CACTGAATAGAGCAACAGCG	No
HBG-Cas9-OT16	chr21:47118131	GaaGGGgAaGGagCCCCAAGAGG	31.6				TCTCCCTTACTGCAACATCCCTA	TAATCAGAGAAGAGCACACA	No
HBG-Cas9-OT17	chr12:118810044	agaGGGgAaGGGtCCCCgAGAGG	30.6				Failed to be amplified		
HBG-Cas9-OT18	chr10:76584623	GgGGGGgAaGGGCCCCCcAGCGG	27.0				CTGGAAAACCGAACTTCGCC	GCGTTCTGGGTGCTTTCTTG	No
HBG-Cas9-OT19	chr1:240818114	GTGGGGAcaGGGCCCCt-AGTGG	24.2			RNA bulge	CAGAGCACGTACAGTCCTGA	TAGTTTTACTGCAGCTCCAAT	No
HBG-Cas9-OT20	chr4:168285109	agtGGGgAaGGGgaCCCAAGCGG	22.0				Failed to be amplified		
HBG-Cas9-OT21	chr18:41189656	GTaGGGAAaGaGCCCCCAcaGGG	18.5						
HBG-Cas9-OT22	chr11:45793336	agaGGGgAaGGGgCCCCtAGAGG	17.6						
HBG-Cas9-OT23	chrX:79404077	GTGGaGAgGGGGCCCCaAAGAGG	16.1						
HBG-Cas9-OT24	chr1:29652860	GcaGGGAAGGaGCCCCCcAtGGG	15.3						
HBG-Cas9-OT25	chr8:42652677	GTGtGGAAGGGGaCCCCAgtGGG	13.0						
HBG-Cas9-OT26	chr17:31958979	aatGGGgAaGGGCCCaaAAGTGG	12.9						
HBG-Cas9-OT27	chr7:128043855	GTGGGGAgGGGtgCCCCAAaGGG	11.6						
HBG-Cas9-OT28	chr2:241478533	GTGtGGAAGGGGaCCCC-AGTGG	10.2			RNA bulge			
HBG-Cas9-OT29	chr12:83382288	aaGGGaAAGGGGCCCttAAGAGG	8.6						
HBG-Cas9-OT30	chr15:54755536	GTGtGGAAGGGGaCCCC-AgtGG	7.8			RNA bulge			
HBG-Cas9-OT31	chr11:71342171	GTGtGGAAGGGGaCCCC-AgtGG	7.7			RNA bulge			
HBG-Cas9-OT32	chr1:36024139	GaGGGGtgGGGtCCCCaAAGGGG	7.5						
HBG-Cas9-OT33	chr10:5452805	GTGtGGAAGGGGaCCCCAgtGGG	7.1						
HBG-Cas9-OT34	chr5:3569470	GTGGaGAAaGGaCCCaCAAGGAG	5.5						
HBG-Cas9-OT35	chr22:19966792	GctaGGAAGGaGCCCCaAAGGGG	5.5						
HBG-Cas9-OT36	chr19:10420980	GTGGGGAcaGGGCCCCt-AGTGG	5.5			RNA bulge			
HBG-Cas9-OT37	chr8:101328581	GTGtGGAAGGGGaCCCC-AGTGG	5.4			RNA bulge			
HBG-Cas9-OT38	chr8:7460562	GTGtGGAAGGGGaCCCC-AGTGG	5.3			RNA bulge			
HBG-Cas9-OT39	chr7:95155534	aTGaGGAAGGGGtCCCC-AGTGG	5.0			RNA bulge			
HBG-Cas9-OT40	chr8:7552583	GTGtGGAAGGGGaCCCC-AGTGG	4.4			RNA bulge			
HBG-Cas9-OT41	chr5:124425202	GTGtGGAAGGGGaCCCC-AGTGG	4.1			RNA bulge			
HBG-Cas9-OT42	chr7:86070727	GTGtGGAAGGGGaCCCC-AGTGG	3.6			RNA bulge			
HBG-Cas9-OT43	chr10:118153708	GTGtGGAAGGGGaCCCC-AGTGG	3.2			RNA bulge			

ABE7.10 and gRNA e Mismatched bases are in lower case. Column is left blank when cleavage is not detected. ABE7.10 and gRNA expression vectors were cotransfected into 293T cells for validation.

Supplementary Table 16. Digenome-seq captured sites using VEGFA3 gRNA and Cas9

Site name	Position	DNA sequence at cleavage site	DNA cleavage score			Bulge	Deep seq primer		Validated
			Cas9	ABE7.10	BE3		FP	RP	ABE7.10
VEGFA3-TA	chr6:43737471	GGTGAGTGAGTGTGTGCGTGTGG	214.5	18.1			GTGCAGACGGCAGTCACTAGG	CTATTGGAATCCTGGAGTGACC	Yes
VEGFA3-Cas9-OT1	chrX:111380725	GGaGAGTGAGTGTGTGaagGAGG	241.0				ATTATGGAGGAAGGGGAAGC	GGAGAAAGAGAGGCAATTGGA	No
VEGFA3-Cas9-OT2	chr17:33323269	tGTGAGTGAGTaTGTaCaTGTGG	203.1				CACTGCCCTTTGGGACTCTG	CCAGAAACAGGTCAGAAGCGA	No
VEGFA3-Cas9-OT3	chr20:50724405	cGTGAGTGAGTGTGTaCcTGGGG	195.5				GTGCTGCTATGATCTTTCTAAAA	CCACTGAGACTGACCTACGTTA	No
VEGFA3-Cas9-OT4	chr7:134234248	aGTGAGTGAGTGaGTGaaTGTGG	189.4				TCTCTATCTCTTCTCCCTCTCTCA	TCTCGAATTCCCTAGCTTTCA	No
VEGFA3-Cas9-OT5	chr5:89440969	aGaGAGTGAGTGTGTGCaTGAGG	175.3	41.5			GTGGGACCTGGTGGGAGT	ACAATCATGGAAGAATGCAAA	No
VEGFA3-Cas9-OT6	chr22:37662824	GcTGAGTGAGTGTaTGCGTGTGG	163.4	7.2			Failed to be amplified		No
VEGFA3-Cas9-OT7	chrX:38624688	taTGAGTGtaTGTGTGCaTaGGG	161.5				AGGTGAGGTGTTCTGAATTATACC	CATAATGCACATGGAGACATTC	No
VEGFA3-Cas9-OT8	chr16:84032646	GGTGAaTGAGTGTGTGCtctGGG	156.5				AGCCCTTTGTTCTTGCTGGT	ACCCCTCTCAAAGACCCTGT	No
VEGFA3-Cas9-OT9	chr11:78871125	aaTGAGTGAGTGaGTGCaTGGAG	153.3				Failed		No
VEGFA3-Cas9-OT10	chr1:212639778	GGgGAaTGAGTGTGTGCaTGGAG	149.4				GAAAGACTCAGGCTGCTTCAACT	GCATTCTGGCTCTCAAGAGAAT	No
VEGFA3-Cas9-OT11	chr6:39028642	GGTGtGTGAGTGTGTGCaTtGGG	148.0				CCCTGAGCTGGGTTGTGTTA	CTCCCTCAGTACCACTGCCT	No
VEGFA3-Cas9-OT12	chr12:102754574	aaTaAGTGAGTGTGTGCaatAGG	147.4				GCCGGAAGGGATTGTCTCAT	GGCAGGAACTCTAGGCAAGG	No
VEGFA3-Cas9-OT13	chr4:89935133	tcTGAGTGAGTGTGgGCaTGGGG	146.0				TTCTCCCCAGGTCTGCATG	TGAGTGGCTGGAACCTACCC	No
VEGFA3-Cas9-OT14	chr1:116485644	aaTGAGTGAGTGTGTGaGTGAAG	145.6				GTGGTGCCACCCCTAACAG	GTCGTGGATATCCTGGCGG	No
VEGFA3-Cas9-OT15	chr6:157078327	GaTGAGTGAGTGaGTGaGTGGGG	145.2	2.2			AGTGTCCAGTGTTGATAAAGTCTA	TTAAAATGATTCACCTGTATAA	No
VEGFA3-Cas9-OT16	chr3:55318919	aGTGAGTGAaTGaGTGCaTaGTG	144.2				TGGCTGCTTTCAGGTAAGGG	AGCAGGGCTTGGCACATAAT	No
VEGFA3-Cas9-OT17	chr7:23792987	taTGAGTGAGTGTGTGgaTGAGG	127.1				TCTCCATATTTGGGTGGTTTT	TCTCAGCAGCTCAGAGCATTA	No
VEGFA3-Cas9-OT18	chr3:10403702	GcatgagtgGgtgtgtgcatTGG	126.1				ATGCCTCCATATGGGCACTG	TCTTTCAGCACCCCTGAAGC	No
VEGFA3-Cas9-OT19	chr11:79178523	aGTGAGTGAGTGaGTGaGTGGGG	121.1	4.8			TCCTTAATGTTTTTGCATTGGAGG	GAACCTCTAGTAGGAGTCGCT	No
VEGFA3-Cas9-OT20	chrX:105612160	aGaGAGTaAGTGTGTaCaTGTGA	120.5				AGTGAATGTGTGAGCATGCCC	CCAAACTCGCTCCCAAATTAG	No
VEGFA3-Cas9-OT21	chr3:38182513	tGTGAGTGAaTGTGTGCcaGGGG	120.3						
VEGFA3-Cas9-OT22	chr5:98946319	GGTGtagtgGTGTGTGCtTGTGG	116.9						
VEGFA3-Cas9-OT23	chr2:199628306	tGTGAGTGAGTGTGTGCagaAGG	114.0						
VEGFA3-Cas9-OT24	chr14:65569159	aGTGAGTGAGTGTGTGtGTGGGG	111.0	15.7			GCTCATTTCCTACGGCCCAG	CTGCAGTGAGGAGGTGGTTC	Yes
VEGFA3-Cas9-OT25	chr8:145090503	tGTGAGTGAaTGTGTGCaTaTGG	109.8						
VEGFA3-Cas9-OT26	chr12:129149692	taTGtGTGAGTGTGTGCaTaTGG	102.8						
VEGFA3-Cas9-OT27	chr7:152671378	aGTGAGTGAGTGaGTGaGTGAGG	101.5	7.2			TGTATTTACTCCATTCACCATCA	CCATGAAGTATGTTCCATCTGA	No
VEGFA3-Cas9-OT28	chr8:128556646	tGTGAGTatGTGTGTGCaTGTGG	99.7						
VEGFA3-Cas9-OT29	chr18:38516378	tGTGAGTatGTGTGTGCaTGTAG	98.8						
VEGFA3-Cas9-OT30	chr2:8573008	acTGAGTGAGTGaGTGaaTGTGG	97.6						
VEGFA3-Cas9-OT31	chr1:229141145	tGTGtGTGAGTGTGTaCaTGAGG	95.5						
VEGFA3-Cas9-OT32	chr19:35213926	taTGAGTGAGTGaGTGaaTGTGG	95.0						
VEGFA3-Cas9-OT33	chr15:29699015	GGaGAGcGAGTGTGTGCaTtTGG	94.9						
VEGFA3-Cas9-OT34	chr7:101077901	tGTGAGTGAGTGTGTtgGTGAGG	94.3						
VEGFA3-Cas9-OT35	chr4:154005628	tGTGAGTGtGTGTGTGCaTGCAG	94.0						
VEGFA3-Cas9-OT36	chr16:49384711	GtgtgtatgagtgtgtgcgtTGG	93.5						
VEGFA3-Cas9-OT37	chr5:90597884	aGTGtagtgGTGTGTGCtTGTGG	92.7						
VEGFA3-Cas9-OT38	chrX:42430834	aGTGAGTGAGTGTGaGCGTGAAG	92.2	1.8			ACATTGCTACACCTTTGGATTCT	ACTGACAAGGTCATTTGATTGG	No
VEGFA3-Cas9-OT39	chr3:1831002	acTGAGTGgGTGTGTGCcTGAGG	90.5						
VEGFA3-Cas9-OT40	chr4:82574191	GGTatGTGAGTGTGTGtaTaTGG	88.6						
VEGFA3-Cas9-OT41	chrX:105614415	aGTGAaTGAGTGTGTGCaTGTGA	88.2						
VEGFA3-Cas9-OT42	chr4:58326608	aGTGAGTGAGTGaGTGaGTGAGG	87.5						
VEGFA3-Cas9-OT43	chr2:171597348	acTGAcTGAGTGTGaGCaTGTGG	81.7						
VEGFA3-Cas9-OT44	chr3:14430297	GGTGAagtgGTGTGTGCcTGTGG	81.5						
VEGFA3-Cas9-OT45	chr7:158305228	tGTGtGTGAGTGTGTGCaTGTGG	81.4						
VEGFA3-Cas9-OT46	chr2:124275984	aGTGAaTGtGTGTGTGCaTGTGG	80.2						
VEGFA3-Cas9-OT47	chr11:90117603	aaTGAGTGAGTaTGTGaaTaCGG	78.9						
VEGFA3-Cas9-OT48	chr12:124763151	tGTGAGTGtGTGTGTaCcTGGGG	78.6						
VEGFA3-Cas9-OT49	chr2:177463426	GGTGAGTGtGTGTGTGCaTGTGG	76.5	7.0			GCGCTTTCCCTTTGCTAGAATC	CTCAGCAATGCTTATATTACTG	Yes
VEGFA3-Cas9-OT50	chr5:178005064	caTGAGTGtGTGTGTGCaTGTGG	75.3						
VEGFA3-Cas9-OT51	chr6:144458291	aGgGAGTGAGTGTGaGaGTGCGG	74.8						
VEGFA3-Cas9-OT52	chrX:56327306	tGTGAGTGtGTGTGTGCaTGTGG	72.7	3.0			ATGAACACCCACATACCCTT	TGACCTCTATTCCACTCACTTT	No
VEGFA3-Cas9-OT53	chr12:5100948	tGTGAaTGAGTGTGTGCaTGTGA	72.6	0.3			Failed to be amplified		
VEGFA3-Cas9-OT54	chr3:193993884	aGTGAaTGAGTGTGTGtGTGTGG	72.3	10.3			CCCTTTGTGACCCAAAAGATTCC	TAAGGCACGAGTCAGGATGGG	No
VEGFA3-Cas9-OT55	chr6:1587476	GGTGtaTGAGaGTGTGCaTGAGG	71.1						
VEGFA3-Cas9-OT56	chr20:2650069	GGTGtaTGAGTGTGTGCGTcGGA	70.9						
VEGFA3-Cas9-OT57	chr8:48997806	GtaGAGTGAGTGTGTGtGTGTGG	68.4						
VEGFA3-Cas9-OT58	chr10:3619371	ccTGAGTGAGTGTGaGaaTGAGG	67.7						
VEGFA3-Cas9-OT59	chr1:88277542	taTGAGTGtGTGTGTGtaTGTGG	65.9						
VEGFA3-Cas9-OT60	chr5:115434676	tGTGgGTGAGTGTGTGCGTGAGG	64.3	1.8			CAATGTGATGATTTGATAGCTG	TCTAATGTATGGCATGGTGACT	No
VEGFA3-Cas9-OT61	chr20:25612568	GGTGtagtgGTGTGTGCcTGTGG	63.9						
VEGFA3-Cas9-OT62	chr4:158621598	aGTGtaTGAGTGTtTGCaTGGGG	63.8						
VEGFA3-Cas9-OT63	chr13:39960714	GGgtgGTGAGTGTGTGacTGTGG	62.6						
VEGFA3-Cas9-OT64	chr2:68869207	tGTGAGTGtaTGTGTGtaTGTGG	62.6						
VEGFA3-Cas9-OT65	chr6:24574540	GGTGtagtgGTGTGTGCcTGTGG	61.6						
VEGFA3-Cas9-OT66	chr19:35051133	tGTGAaTGAGTGcGTGaaTGTGG	61.6						
VEGFA3-Cas9-OT67	chr2:126515435	tGTGAGTGAaTaTGTGtaTGTGG	59.7						
VEGFA3-Cas9-OT68	chr13:90418142	tGTGtGTGAGTGTGTGtaTGGGG	59.0						
VEGFA3-Cas9-OT69	chr19:47787100	GaTGAGTGtGTcTGTGCaTGAGG	58.9						
VEGFA3-Cas9-OT70	chr6:24224744	GGTGAGcGtGTGTGTGCaTGTGG	57.8	1.1			GGGGTACAATGGTGCACAGA	TGCCACCCCAGTTTTGAGTT	No
VEGFA3-Cas9-OT71	chr12:95277430	GGTGtagtgGTGTGTGCcTGTGG	57.6						
VEGFA3-Cas9-OT72	chr2:18696225	aGTGAGaaAGTGTGTGCaTGCGG	56.8						
VEGFA3-Cas9-OT73	chr3:43415188	tcaGAaTGAGTGTGTGCcTGGGG	56.8						
VEGFA3-Cas9-OT74	chr1:203434970	caTaAGTGAGTGTGTGCGaGTGG	56.3						
VEGFA3-Cas9-OT75	chr1:60407665	GGTGAGTcAGTGTGTGaGTGGAG	56.1						
VEGFA3-Cas9-OT76	chr14:62078773	tGTGAGTaAGTGTGTGtGTGTGG	56.0	11.2			GCCACAGGCACTAACTTCTTCA	GATGAAGCTGCCTTTCCTAAGC	Yes
VEGFA3-Cas9-OT77	chr21:26653015	GGTGtGTGtGTGTGTGCaTGTGG	54.0						
VEGFA3-Cas9-OT78	chr17:31551606	aaTGAaTGAaTGTaTGCaTGTGG	54.0						
VEGFA3-Cas9-OT79	chr10:1684972	tGTGAGTGgGTGTGTGaGTGAGG	53.6						
VEGFA3-Cas9-OT80	chr12:84424854	caTGAGTGAaTGTGTaCaTGTAG	52.8						
VEGFA3-Cas9-OT81	chr20:20178284	aGTGtGTGAGTGTGTGCGTGTGG	52.5						
VEGFA3-Cas9-OT82	chr8:107510883	tGTGAGTGtGTGTGTGaGTGTGG	49.8						
VEGFA3-Cas9-OT83	chr14:74353497	aGcGAGTGgGTGTGTGCGTGGGG	49.7						
VEGFA3-Cas9-OT84	chr5:29367379	tGTGAGTGAGTGTGTGtaTGGGG	49.1	20.3			TTTTGTTTCTAAAAATTAAACTAA	TGTTCCATTGTCTGAAATGTAT	No
VEGFA3-Cas9-OT85	chr12:592739	GGTGtagtgGTGTGTGCcTGTGG	48.6						
VEGFA3-Cas9-OT86	chr3:68062404	tGTGAGTGtGTGTGTaCaTaTGG	48.3						
VEGFA3-Cas9-OT87	chr4:7430877	caTGcGTGAGTGTGTGCaTGGGG	47.3						
VEGFA3-Cas9-OT88	chr20:21927847	GaaGAaTGAGTGTGTGCtTGTGG	46.8						
VEGFA3-Cas9-OT89	chrX:29527804	GGTGtGTGtGTGTGTGCGTGTGG	46.7						
VEGFA3-Cas9-OT90	chr5:149501694	GaTGAGTGAGTGTGTGaGTGAGA	46.4						

VEGFA3-Cas9-OT91	chr2:236425371	aGTGgaTGAGTGaGTGCaTGCGG	46.4				
VEGFA3-Cas9-OT92	chr9:23824554	tGTGgGTGAGTGTGTGCGTGAGA	45.4				
VEGFA3-Cas9-OT93	chr7:155055783	aGTGtGTGAGTGTGTGtcTGAGG	44.6				
VEGFA3-Cas9-OT94	chr4:166898498	GGcGAGaGAGTGTGTGCagGAGG	44.2				
VEGFA3-Cas9-OT95	chr4:66528433	aGTGtGTGAGaGTGTGtaTGTGG	43.6				
VEGFA3-Cas9-OT96	chr11:68851139	aGTGgGTGAGTGaGTGCGTGCGG	43.5				
VEGFA3-Cas9-OT97	chr10:109378067	GGTGAGTGAGTGaGTGaGTGAGG	41.7	1.4	AAAGTCTGTGGTAGTGTATAGTA/	ATATAGTATAAGAGATAAAAA/	No
VEGFA3-Cas9-OT98	chr1:58583929	aGTGAGTGAGTGaGTGaaTGAGG	41.2				
VEGFA3-Cas9-OT99	chr7:100892441	GGTGtagtgGTGTGTGCcTGTGG	41.0				
VEGFA3-Cas9-OT10(	chr10:45209678	aGgtAGTGAGTGTGTGCaTGGGT	41.0				
VEGFA3-Cas9-OT10	chr19:11949610	GGTGtagtgGTGTGTGCcTGTGG	40.4				
VEGFA3-Cas9-OT10.	chr1:18401508	GGTGtagtgGTGTGTGCcTGTGG	39.6				
VEGFA3-Cas9-OT10:	chr14:98442534	GGTGAGTGtGTGTGTGaGTGTGG	38.8	2.0	Failed to be amplified		
VEGFA3-Cas9-OT10·	chr10:107867379	aGaGAGTGAGTGTGTGtGTtGGG	38.4	0.3	Failed to be amplified		
VEGFA3-Cas9-OT10!	chr11:115758116	aGaGAGTGtGTGTGTGCtTGGGG	36.4				
VEGFA3-Cas9-OT10(	chr9:89609939	tGgGAGTGAGTGTGTtCaTGGGG	36.1				
VEGFA3-Cas9-OT10'	chr10:4057855	GcTGAGTGgGTGTGTGCaTGCAG	35.2				
VEGFA3-Cas9-OT10(	chr7:123613527	tGTGAGTGtaTGTGTGCaTcAGG	34.9				
VEGFA3-Cas9-OT10!	chr5:56989649	aaTGAGTGtGTGTGTGtaTGGGG	34.3				
VEGFA3-Cas9-OT11(	chr2:74655959	GGTaAGTatGTGTGTGCaTGGGG	33.4				
VEGFA3-Cas9-OT11	chr17:64940809	aaTGAGTGAGTGaGTGaaTGAGG	33.0				
VEGFA3-Cas9-OT11.	chr1:48691305	aGTGtGTGAGaGTGTGCaTGTGG	32.8				
VEGFA3-Cas9-OT11:	chr16:21710833	GGTGtagtgGTGTGTGCcTGAGG	32.8				
VEGFA3-Cas9-OT11·	chr21:26977711	tGTGAGTGgGaaTGTGCaTGGGG	32.6				
VEGFA3-Cas9-OT11!	chr22:39333827	tGTGtGTGAGTGTGTGtaTGTGG	31.9				
VEGFA3-Cas9-OT11(	chr15:65080871	GGTGtagtgGTGTGTGCcTGTGG	31.4				
VEGFA3-Cas9-OT11'	chr20:62468987	aGTGAGTGAGTGaGTGaaTGAGG	31.1				
VEGFA3-Cas9-OT11(	chr10:95051225	ccTGAGcGAGTaTGTGCaTGTGG	31.0				
VEGFA3-Cas9-OT11!	chr2:141610261	atTGAGTaAGTaTGTaCaTGTGG	30.3				
VEGFA3-Cas9-OT12(	chr18:70744216	tGTGAGTGtGTGTGTGCaTGTGC	28.8				
VEGFA3-Cas9-OT12	chr10:7046008	GtgagtgtgtgtgtTGCaTGTGG	28.7				
VEGFA3-Cas9-OT12.	chr4:113550701	GGTGAGgtgGTGTGTGCcTGTGG	27.7				
VEGFA3-Cas9-OT12:	chr13:60101377	GGTaAaTGAGTGTGaGgcatGGG	27.7				
VEGFA3-Cas9-OT12·	chr21:46066307	tGTGtGTGAGTGaGTGaGTGAGG	27.6				
VEGFA3-Cas9-OT12!	chr14:84459815	GGgtgagatGTGTGTGCaTGTGG	27.0				
VEGFA3-Cas9-OT12(	chr13:28298848	aaTGgaTGAGTGTGTGCaTcTGG	26.9				
VEGFA3-Cas9-OT12'	chr2:166156892	tGTGAGTGtGTGTGTGtGTtTGG	26.4				
VEGFA3-Cas9-OT12(	chrX:39952122	tGTGAaTaAGTGTGTGaaTGTGG	26.0				
VEGFA3-Cas9-OT12!	chr18:75912617	GGaGAGTGtGTGTGTGaGTGTGG	25.9				
VEGFA3-Cas9-OT13(	chr9:21430082	aGgGAGTGAGaGTGTGaaTGTGG	25.5				
VEGFA3-Cas9-OT13	chr4:31116235	taTGtGTGAGTGTGTGCaTGGTG	25.4				
VEGFA3-Cas9-OT13.	chr17:79111961	GGTaAGTGtGTGTGTGCaTGTGG	25.2				
VEGFA3-Cas9-OT13:	chr17:58739982	GaaGAGTGAaTGTGTGCcTaTGG	24.9				
VEGFA3-Cas9-OT13·	chr7:157924672	tGTGtGTGAGTGTGTGtGTtGGG	24.0				
VEGFA3-Cas9-OT13!	chr15:94548473	tGaagGTGAGTGaGTGCaTGGGG	23.7				
VEGFA3-Cas9-OT13(	chr11:61485469	GcaGAGTGAGTGTGTGtGTtGGG	23.2				
VEGFA3-Cas9-OT13'	chr5:3334493	caTGAGTGtGTGTGTGCaTGCAG	22.9				
VEGFA3-Cas9-OT13(	chr9:95974277	tGTGAaTGAGTGcGTGaaTGGGG	22.9				
VEGFA3-Cas9-OT13!	chr14:30033865	GGTGAGgtgGTGTGTGCtTGTGG	22.2				
VEGFA3-Cas9-OT14(	chr2:6043502	aaTGAGTGAaTGaaTGCaTGCGG	22.2				
VEGFA3-Cas9-OT14	chr9:135252580	GGTGtGTGAGTGcaTGCaTGTGG	22.2				
VEGFA3-Cas9-OT14.	chr11:95730474	tGTGAGTGtGTGTGTGCaTGCGC	21.8				
VEGFA3-Cas9-OT14:	chr13:87931160	aGTGAGTGtGTGTGTGtaTGTGA	21.2				
VEGFA3-Cas9-OT14·	chr17:43132890	aagtgagGAGTGTGTGCcTGTGG	21.2				
VEGFA3-Cas9-OT14!	chr16:88218507	atTGtGTGAGTGTGTGCaTGTGG	21.0				
VEGFA3-Cas9-OT14(	chr3:172121469	GGgaAGgGAGTGTGTGCaTGGGG	20.9				
VEGFA3-Cas9-OT14'	chr5:100244230	taTGAGTGAGTGaGaaCaTGCGG	20.0				
VEGFA3-Cas9-OT14(	chr8:121823447	tGaGtGTGAGTGTGaGCGTGCGG	19.7				
VEGFA3-Cas9-OT14!	chr19:36171459	GGTGtagtAGTGTGTGCcTGTGG	19.7				
VEGFA3-Cas9-OT15(	chr2:230885194	GGTGtagtgaTGTGTGCcTGTGG	19.3				
VEGFA3-Cas9-OT15	chr16:5844943	tGaGAGTGgGTGTGTGCcTGGGG	19.1				
VEGFA3-Cas9-OT15.	chr9:110103705	GGgaAagacccagcatCcgtGGG	18.5				
VEGFA3-Cas9-OT15:	chr1:22117219	aGTGAtgGAGTGTGTGCcTGTGG	18.2				
VEGFA3-Cas9-OT15·	chr13:74053255	aGTGAGTGAGTGaaTGaaTGGGG	17.3				
VEGFA3-Cas9-OT15!	chr1:190857306	tGTGtGTGAaTGTGTGCaTGTGG	16.8				
VEGFA3-Cas9-OT15(	chr17:54749751	tGTGAGgGAGTGTGTGCaTGCGA	16.5				
VEGFA3-Cas9-OT15'	chr11:127942322	tGTGtGTGAGTGTGTGtaTtTGG	16.4				
VEGFA3-Cas9-OT15(	chr12:97780497	GaTGAGTGtGTGTGTGtGTtGGG	16.1				
VEGFA3-Cas9-OT15!	chr21:44179977	tGTGAGTGgGTGTGTGCaTGTGG	15.2				
VEGFA3-Cas9-OT16(	chr17:74046702	cGTGAGTGAGTGTGTGgtTGGGG	15.1				
VEGFA3-Cas9-OT16	chr3:19532667	tGTGAGTGtGTGTGTGaaTGGAG	15.0				
VEGFA3-Cas9-OT16.	chr4:24692989	tGaGtGTGAGTGTGTGCaTGGGG	14.1				
VEGFA3-Cas9-OT16:	chr7:3009164	GGTGAaTGAGTGaGTGCaTGGGA	13.9				
VEGFA3-Cas9-OT16·	chr9:135253621	GGTGtGTGAGTGcaTGCaTGTGG	13.5				
VEGFA3-Cas9-OT16!	chr17:40309769	GGTGtagtgGTGTGTGCcTGTGG	13.4				
VEGFA3-Cas9-OT16(	chr4:95324499	GGTGttgtAGTGTGTGCcTGTGG	13.0				
VEGFA3-Cas9-OT16'	chr7:134710951	GtgatGTGAGaaTGTGCtTGGGG	12.9				
VEGFA3-Cas9-OT16(	chrX:41726218	GGTGAGTGAGTGaGTGaGTGAGG	12.5	7.0	GCATACTAGACTAGGGGTTCTGC	TTCCCAAACAGTGTGCCATGAT	No
VEGFA3-Cas9-OT16!	chr11:1004348	GGTGtagtgGTGTGTGCcTGTGG	12.4				
VEGFA3-Cas9-OT17(	chr12:68071348	aaTGAGTGAGTGaGTGaGTaAGG	12.2				
VEGFA3-Cas9-OT17	chr19:5494009	GGTGttgtgGTGTGTGCcTGTGG	12.0				
VEGFA3-Cas9-OT17.	chr11:23319383	GGTGAGTtAGTGaGTGaGTGGGG	11.7				
VEGFA3-Cas9-OT17:	chr18:50778055	tGTGAGTGtGTGTGTGCaTGCAA	11.6				
VEGFA3-Cas9-OT17·	chr4:126883064	GGTGtGTGAGTGaGTGaaTGTGG	11.6				
VEGFA3-Cas9-OT17!	chr1:218094693	aGTGAGaGAGTGTGTGCaccTGG	11.4				
VEGFA3-Cas9-OT17(	chr6:71411397	aGaGAGaGAaTGTGTGCtTGGGG	11.4				
VEGFA3-Cas9-OT17'	chr12:104265369	aGTcAGTGAaTGTGaGCaTGTGG	11.4				
VEGFA3-Cas9-OT17(	chr5:11347237	tGaGAGgGAGTGTaTGCaTGGGG	11.3				
VEGFA3-Cas9-OT17!	chr2:765652	taTGAaTGtGTGTGTGCaTGTGG	11.2				
VEGFA3-Cas9-OT18(	chr16:67054971	GGTGgagtgGTGTGTGCcTGTGG	11.1				
VEGFA3-Cas9-OT18	chr12:123152629	GGTGtGgtgGTGTGTGCtTGTGG	10.7				
VEGFA3-Cas9-OT18.	chr18:73030546	tGTGAGTGcaTGTGTGCcTGTGG	10.7				
VEGFA3-Cas9-OT18:	chr6:145990148	taTGAGTGAGTGTGTGaGTaGGT	10.5				
VEGFA3-Cas9-OT18·	chr8:3893422	GGTGAGTctGTGTGTGaGTGAGG	10.4				

VEGFA3-Cas9-OT185	chr17:46453599	GcTGAGTGAaTGTGTGacTGTGG	10.2
VEGFA3-Cas9-OT186	chr17:68434784	aGTGAGTGtGTGTGTGCaTGTGC	10.0
VEGFA3-Cas9-OT187	chr20:35587689	GGTGAGgtgGTGTGTGCcTGTGG	9.7
VEGFA3-Cas9-OT188	chr5:1498646	taTGAGTGtGTGTGTGCaTGTGA	9.1
VEGFA3-Cas9-OT189	chr10:96194539	GGTGttgtgGTGTGTGCcTGTGG	8.5
VEGFA3-Cas9-OT190	chr12:127030042	GtgagtgagtgagtgagtgGTGG	8.1
VEGFA3-Cas9-OT191	chr13:31628698	GGTGAcTGtGTGTGTGCaTGGGG	8.1
VEGFA3-Cas9-OT192	chr7:105362464	aGTGAGTGAGTGTGTGtaTGCGC	8.1
VEGFA3-Cas9-OT193	chr17:56122760	GGTGttgtgGTGTGTGCcTGTGG	7.4
VEGFA3-Cas9-OT194	chr20:25294293	GGTGtagtgGTGTGTGCcTGTGG	7.3
VEGFA3-Cas9-OT195	chr12:129509970	tGTGAaTGtGTGTGTGCGTGTGG	7.3
VEGFA3-Cas9-OT196	chr16:63278898	ccTGAGTGAGTGTGaGtaTGCAG	7.2
VEGFA3-Cas9-OT197	chr12:96644881	acgGAGTGAGTGTGTGCtgtTGG	7.2
VEGFA3-Cas9-OT198	chr1:7207656	taTGAGTGtGTGTGTGCaTGGTG	7.0
VEGFA3-Cas9-OT199	chr1:78331153	GGTGcagtgGTGTGTGCcTGTGG	7.0
VEGFA3-Cas9-OT200	chr5:10193580	GGTGtagtgGTGcGTGCcTGTGG	6.9
VEGFA3-Cas9-OT201	chr16:70633721	GGTGtagtAGTGTGTGCtTaTGG	6.7
VEGFA3-Cas9-OT202	chr11:84931454	ctaGAGTaAGaGTGTGCcTGGGG	6.6
VEGFA3-Cas9-OT203	chr22:24612688	caTGAGTGgGTGTGTGCGgtGGG	6.5
VEGFA3-Cas9-OT204	chr9:139474807	aGTGAGTGAGTGaGTGaaTGAGG	6.4
VEGFA3-Cas9-OT205	chr18:13888449	GtTGAGTGAGTGaaTGCaTcTGG	6.1
VEGFA3-Cas9-OT206	chr7:70811180	GtgagaTGAGTGTGTaCaTGTGA	6.1
VEGFA3-Cas9-OT207	chr4:187327499	caTGAGTGtGTGTGTGaGTGTGG	5.7
VEGFA3-Cas9-OT208	chr1:63699577	aGaGAGTGAGTGTGTGaagGAGG	5.6
VEGFA3-Cas9-OT209	chr6:51088637	tGgGAGTGtGTGTGTGCaTGTGG	5.6
VEGFA3-Cas9-OT210	chr1:12258495	acgtAGTGAGTGTGTGCGctGGG	5.5
VEGFA3-Cas9-OT211	chr9:75406588	tGTGAcTGAGTGTGTGtaTGTGC	5.5
VEGFA3-Cas9-OT212	chr7:40593326	tGTGAGTGtGTGTGTGCaTGTGC	5.3
VEGFA3-Cas9-OT213	chrX:155174882	aGaGAGTGAGTGTGTGaagGAGG	5.2
VEGFA3-Cas9-OT214	chr13:38740462	tGTaAGTGAGTaaGTGCcTGTGG	5.1
VEGFA3-Cas9-OT215	chr13:57506798	aaTtAGTaAGTGTGTGCGTGGAG	4.9
VEGFA3-Cas9-OT216	chr18:73353353	tGTGtGTGtGTGTGTGCtTGAGG	4.7
VEGFA3-Cas9-OT217	chr20:18577482	aaTGAGTGAGccTaTGCaTGCGG	4.7
VEGFA3-Cas9-OT218	chr4:92494745	GcTGAGTGAaTGTGTGaaTtGGG	4.5
VEGFA3-Cas9-OT219	chr11:69410585	tGTGAGTatGTGTGTGCaTGTGA	4.1
VEGFA3-Cas9-OT220	chr8:8422365	GGTGtGTGAGTGTGTGaGatGGG	3.9
VEGFA3-Cas9-OT221	chr12:104466025	GGTGtagtgGTaTGTGCcTGTGG	3.9
VEGFA3-Cas9-OT222	chrY:59277888	aGaGAGTGAGTGTGTGaagGAGG	3.7
VEGFA3-Cas9-OT223	chr5:155118304	tGTGAGaGAGTGTGTGCaTGTGA	3.6
VEGFA3-Cas9-OT224	chr8:24755958	GaTGgGTGAGTGTGTGtaTtTGG	3.3
VEGFA3-Cas9-OT225	chr2:6028042	ctTttGTGAGTGTGTGCtTGAGG	3.2
VEGFA3-Cas9-OT226	chr3:63018437	aGTGAGTGAGTGgGTGCaTGAAG	3.1
VEGFA3-Cas9-OT227	chr1:181721049	taTGAGTGtGTacGTGCaTGTGG	3.0
VEGFA3-Cas9-OT228	chr17:77170739	GtgtgagtgGTtgtgtgcatTGG	2.8
VEGFA3-Cas9-OT229	chr21:37365836	tGTGAGTGtGTGTGTGaGTtGGG	2.6
VEGFA3-Cas9-OT230	chr4:125665237	taTatGTGAGTGTGaGCaTGTGG	2.5
VEGFA3-Cas9-OT231	chr8:55727414	GGgGAGaGAGTGTGTGCaTGTTG	2.5

ABE7.10 and gRNA expression vectors were cotransfected into 293T cells for validation.

Supplementary Table 17. Digenome-seq captured sites using HEK293-2 gRNA and Cas9

Site name	Position	DNA sequence at cleavage site	DNA cleavage score			Bulge	Deep seq primer		Validated
			Cas9	ABE7.10	BE3		FP	RP	ABE7.10
HEK293-2-TA	chr5:87240614	GAACACAAAGCATAGACTGCGGG	131.7	19.4	27.4		ACAATGATAACAAGACCTGGCTG	CCCCATCTGTCAAACTGTGCG	Yes
HEK293-2-Cas9-OT1	chr19:40272373	atACACAAAGCATAG--TGCTGG	135.3			RNA bulge	CCTTCGCCTTCTGTCATGAGT	TTGGTGCATGTGTTAGTCCATT	No
HEK293-2-Cas9-OT2	chr2:201358910	agACACAAAGC--AGACTGCTGG	130.6			RNA bulge	CACCAAACACAATAGGTCACATT	TCACCCCAAAAGGAACCC	No
HEK293-2-Cas9-OT3	chr15:93557679	GAACACA-tGCATAGACTGCTAG	126.9	35.3	37.0	RNA bulge	CTCTGAGAGTGCCGCCAG	ACTGGCGTTTACTACCTCCT	No
HEK293-2-Cas9-OT4	chrX:39421866	GtACACAAAGCATAG--TGCCGG	125.0			RNA bulge	ATTTTGCATTGCTATAAAGGAA	TCTCACTCTATGACACACTGGC	No
HEK293-2-Cas9-OT5	chr16:74652461	tAACACAAAGtATAGACTG-GGA	116.6			RNA bulge	Failed to be amplified		No
HEK293-2-Cas9-OT6	chr12:823921	GtACACAAAGCATAG--TGCCGG	107.0			RNA bulge	Failed to be amplified		No
HEK293-2-Cas9-OT7	chr13:55564919	acACACAAAGCAT-GACTGCAGG	96.0			RNA bulge	AGAAAAGTGCCTTAGGATGCCA	ATGTACTTCCTTTACTCAGACACT	No
HEK293-2-Cas9-OT8	chr1:112863195	GAAgACAAAGCATAGACa--TGG	94.1			RNA bulge	TCCCTACCTTCCAGTTACTTACCA	TCTCCAGTCACAGTACCCCTA	No
HEK293-2-Cas9-OT9	chr4:90522184	GAACACAAtGCATAGAtTGCCGG	82.1		33.6		TAGTCCAGATAATCAGAGTGTGGAC	TCTTGTGAAACAGAAATGTCAGTTA	No
HEK293-2-Cas9-OT10	chr18:22360702	GAAt-CAAAGCAcAGACTGCAGG	80.4		19.5	RNA bulge	CACACTGAGTGGCAAGCCT	AAGCCACCCGTTTTAGTGGT	No
HEK293-2-Cas9-OT11	chr2:178773997	GtACACAAAaCA-AGACaG-CAG	72.2			RNA bulge	ACTCTGCCATTTTAAGCCAATTACA	GAGTTGGGTGTTCATTTTGCCA	No
HEK293-2-Cas9-OT12	chr8:72061268	GtACc-AAAGCAcAGACTtCCGG	68.8			RNA bulge	AGGATGGCCCTGAACAAGGT	TGCCTCAACTGCTTCCTTCT	No
HEK293-2-Cas9-OT13	chr18:10441546	aAtCACAAAGCATAGA--GCTGG	68.3			RNA bulge	CAGTGGTGTCTGTGAGGCAA	ATTGCACAAGCTAGGAGGGC	No
HEK293-2-Cas9-OT14	chr7:83764327	ctAtA-tAAGCATAGACTGtTGG	68.1			RNA bulge	GCAAGGTAACAAAGCAAAACA	AGCAAAACTCCATCTCAAAAA	No
HEK293-2-Cas9-OT15	chr2:19844956	aActcCAAAGCATAtACTGCTGG	65.9		28.5		TTTGACTTCACTGGAGAGAAAGAG	CTCCTAAAAGCCTCCATTCACC	No
HEK293-2-Cas9-OT16	chr11:89184732	aAACACtAAGCATAG--TGCTGG	63.9			RNA bulge	CTATGCCTGAGGTTTCTTGTGTG	GACATGTCACCTGCCAAGATGAT	No
HEK293-2-Cas9-OT17	chr3:86028116	cAACACAAAGCAaAGACTtgGAG	58.7				TTTTGCTACTGCATGTCAAACT	GAATATCAGGTACAAAACTGTGTCA	No
HEK293-2-Cas9-OT18	chr2:174899900	GcACc-AAAGCAcAGACTGCTGG	50.3			RNA bulge	ACTGGCTCCATTCATCCTGG	TGAGCCCGTATTGCTTTGTTG	No
HEK293-2-Cas9-OT19	chr3:19738731	tAACACgAAGCATAG--TGCTGG	48.3			RNA bulge	TGATTGAAATCTCCCTGAGG	GCTATTCTTGCATTGCCATA	No
HEK293-2-Cas9-OT20	chr8:52596627	GAACACAtAGCATAGA-TatTGG	47.9	3.2		RNA bulge	TTCCCTCCCACCCACTTACA	GCATGTGCTAGCAGCCATAA	No
HEK293-2-Cas9-OT21	chr1:167742859	aAACACAgAGCAcAGACTGCTGA	47.5						
HEK293-2-Cas9-OT22	chr9:73294821	tgAacCAcAaagcAtACTG-CAG	46.2			RNA bulge			
HEK293-2-Cas9-OT23	chr1:175310137	GgACACAAAGCtTAGACT-CCAG	41.6			RNA bulge			
HEK293-2-Cas9-OT24	chr10:43159340	aAcacaAAgaCATAGACcaCTGG	39.4						
HEK293-2-Cas9-OT25	chr5:7625837	GtACACA-AtaATAGACTGCAGG	38.4	21.7	25.2	RNA bulge			
HEK293-2-Cas9-OT26	chr3:168165118	GAACA--cAGCAaAGACTGCTGG	38.0			RNA bulge			
HEK293-2-Cas9-OT27	chr1:77190607	tcACACAAAcCATAGACTG-AGG	37.4			RNA bulge			
HEK293-2-Cas9-OT28	chr1:199217097	tAcCA-tAAGCATAGACTGtTGG	36.9			RNA bulge			
HEK293-2-Cas9-OT29	chr4:102190892	aAACACAAAG--TAtACTGCTGG	36.7			RNA bulge			
HEK293-2-Cas9-OT30	chr4:53536210	GAAtACtAAGCATAGACTcCAGG	35.3						
HEK293-2-Cas9-OT31	chr20:97641	GAAttCAAAGCATAGAtTGCAGG	34.6						
HEK293-2-Cas9-OT32	chr2:101900018	tAACt-AAAGCATAcACTGCAGG	31.4			RNA bulge			
HEK293-2-Cas9-OT33	chr1:40461425	acACAC-AAGCAcAGACTGCAGG	30.6			RNA bulge			
HEK293-2-Cas9-OT34	chr19:35505486	GAACAC-AAGCAcAGACTGaAGG	30.2	9.2		RNA bulge			
HEK293-2-Cas9-OT35	chr14:49426023	aAACACAAAG--TAGAaTGCTGG	30.0			RNA bulge			
HEK293-2-Cas9-OT36	chr3:28560566	GAACACAAAGtATAGAaTGCTAG	29.1						
HEK293-2-Cas9-OT37	chr6:108699823	GAACtCAAAGCATAGgCcGgGTG	29.1						
HEK293-2-Cas9-OT38	chr1:66454109	GtAtA-AcAGCATAcACTGCTGG	28.4			RNA bulge			
HEK293-2-Cas9-OT39	chr6:88664159	GAtttCAAAGCATAGA-TGCTGG	27.1			RNA bulge			
HEK293-2-Cas9-OT40	chr18:31607486	atACACAAAGCAT--AcTGCTGG	25.8			RNA bulge			
HEK293-2-Cas9-OT41	chr6:122263033	GAACAC---tCATAaACTGCTGG	25.3			RNA bulge			
HEK293-2-Cas9-OT42	chr6:97960147	GtgCACAAAGCATAG--TGCTGG	25.0			RNA bulge			
HEK293-2-Cas9-OT43	chr4:109507768	tAACACAgAGCATAGA--GCAGG	24.6			RNA bulge			
HEK293-2-Cas9-OT44	chr7:112272096	ttACc-AtAGCATAGgCTGCTGG	24.5			RNA bulge			
HEK293-2-Cas9-OT45	chr16:64198278	GAAtACAtAaCATAGACT--GGG	24.2			RNA bulge			
HEK293-2-Cas9-OT46	chr1:36097072	GtAaACAAAGCATAGACTG-AGG	23.4			RNA bulge			
HEK293-2-Cas9-OT47	chr3:28595792	GtACA-AAAGCATAGACcaaGGG	23.3			RNA bulge			
HEK293-2-Cas9-OT48	chr13:61611392	cAACA-AAAGCATAGACT-CTAG	21.9			RNA bulge			
HEK293-2-Cas9-OT49	chr15:42563125	ttACttAttGCATAGACTG-TGG	21.8			RNA bulge			
HEK293-2-Cas9-OT50	chr1:67142257	GAAC-CActGCATAGAaTGCAGG	21.8		8.0	RNA bulge			
HEK293-2-Cas9-OT51	chr3:142184567	aAAaACAAAGCATAGACT--GGG	21.8			RNA bulge			
HEK293-2-Cas9-OT52	chrX:132724932	GAACA-tAAtCAcAGACTGCTGG	21.5			RNA bulge			
HEK293-2-Cas9-OT53	chr8:78120475	GAAgAC-AAGCATAGACaGtGGG	21.4			RNA bulge			
HEK293-2-Cas9-OT54	chr1:166352460	ttAtt-AAAGCATAGAtTGCTGG	20.5			RNA bulge			
HEK293-2-Cas9-OT55	chr2:192248364	GAACAC-AtaCATAGACaGCTGG	20.4		1.6	RNA bulge			
HEK293-2-Cas9-OT56	chr5:124378475	agACACAAAGC-TAaACTGCTGG	20.2			RNA bulge			
HEK293-2-Cas9-OT57	chr9:13429069	--cAACACAAAaCATAGACcAGG	20.1			RNA bulge			
HEK293-2-Cas9-OT58	chr11:80540770	GAtCt-tAAGCATAGACTG-TGG	19.9			RNA bulge			
HEK293-2-Cas9-OT59	chr20:895768	tttCA-AcAGCATAGACTG-TAG	19.6			RNA bulge			
HEK293-2-Cas9-OT60	chr6:164073518	cttgctAAAGCAcAGACTGCTGG	19.2						
HEK293-2-Cas9-OT61	chr14:74911965	GtACACAAAGCAT--AaTGCTGG	19.2			RNA bulge			
HEK293-2-Cas9-OT62	chr8:74980814	GtAgAgtAAGCATAcACTGCTGG	18.9						
HEK293-2-Cas9-OT63	chr6:114764429	acAacacAAaCATAGcCTGCTGG	18.8						
HEK293-2-Cas9-OT64	chr1:14872658	cctacCccAGCATAGACTGCAGC	18.7						
HEK293-2-Cas9-OT65	chr5:95665558	ttgCACAAAGCAcAGACTGCTGA	18.7						
HEK293-2-Cas9-OT66	chr4:170242083	tAACAaAAtGCATAGACTGCTAG	18.6						
HEK293-2-Cas9-OT67	chrX:144587673	cttgttAAAGCAcAGACTGCTGG	18.4						
HEK293-2-Cas9-OT68	chr13:54660957	GAACACA---tATAGACTGCTGG	18.3			RNA bulge			
HEK293-2-Cas9-OT69	chr3:30056215	acACAg-AAaCATAG-CTGCTGG	17.8			RNA bulge			
HEK293-2-Cas9-OT70	chr10:65717412	GAACAC-tctCATAcACTGCTGG	17.4	0.6		RNA bulge			
HEK293-2-Cas9-OT71	chr19:28824656	GAACtCAAAGCATAGAaTaaTGG	16.7						
HEK293-2-Cas9-OT72	chr18:45715538	GggCACAAAGCAT-GACTGCTGG	16.1			RNA bulge			
HEK293-2-Cas9-OT73	chr11:19349649	ttgtt-AAAaCATAGACTGCTGG	15.5			RNA bulge			
HEK293-2-Cas9-OT74	chr20:23101380	atACACAgAGCAaAGACTGCAGG	14.9						
HEK293-2-Cas9-OT75	chr18:5596266	ctACAg-AAGCATAGACTG-CAG	14.8			RNA bulge			
HEK293-2-Cas9-OT76	chr1:37171403	GgACAC-AAGCATAGACatCTGG	14.4			RNA bulge			
HEK293-2-Cas9-OT77	chr2:75813045	aAggAacActCATAGACTGCTGG	14.3						
HEK293-2-Cas9-OT78	chr20:12760770	GAACAatAAGCATAGACT--GGG	14.3			RNA bulge			
HEK293-2-Cas9-OT79	chr2:42080737	acAtt-AAAGCAcAGACTGCTGG	14.2			RNA bulge			
HEK293-2-Cas9-OT80	chr19:13573879	GcttA-AAAGCATAGACTG-TGG	13.9			RNA bulge			
HEK293-2-Cas9-OT81	chr21:25643685	acACA-tAtGCATAGACTGCAAG	13.0			RNA bulge			
HEK293-2-Cas9-OT82	chr20:12633883	GttgtgAAAGCATAGAtTGCTGG	12.9						
HEK293-2-Cas9-OT83	chr3:5604367	ttgtt-AAAGCATAGAtTGCTGG	12.5			RNA bulge			
HEK293-2-Cas9-OT84	chr11:106849553	atgatgAAAGCATAGACTGCGTG	12.4						
HEK293-2-Cas9-OT85	chr5:142142325	aAACAC-tAaCATAGACTG-CAG	12.4			RNA bulge			
HEK293-2-Cas9-OT86	chr22:28895718	attaAgAtAGCATAGACTGCAGG	12.3						
HEK293-2-Cas9-OT87	chr11:97148029	GgAgAgAAAGCATAcACTGCTGG	12.0						
HEK293-2-Cas9-OT88	chr2:209216355	GtAtACAAAGCATAG--TGCTGG	12.0			RNA bulge			
HEK293-2-Cas9-OT89	chr1:88546904	acACACAAAgCATAGACT--GGG	11.6			RNA bulge			
HEK293-2-Cas9-OT90	chr10:85754684	cttgttAAAGCATAGAtTGCTGG	11.1						
HEK293-2-Cas9-OT91	chr5:59782910	cttCt-AtAGCATAcACTG-CAG	10.9			RNA bulge			
HEK293-2-Cas9-OT92	chr2:34558180	GAACcCAtAGCA-AGACTGCCGG	10.6			RNA bulge			
HEK293-2-Cas9-OT93	chr1:176037521	ttACACAtAGCAcAGACTaCAGG	10.4						
HEK293-2-Cas9-OT94	chr22:29278678	accCtCtAtGCAcAGACTGCTGG	10.3						
HEK293-2-Cas9-OT95	chr10:5534342	ctACAC-AAGCATAGACaG-CAG	10.2			RNA bulge			
HEK293-2-Cas9-OT96	chr2:205868325	aAACACAAAGC--AGACTGtGGG	10.1			RNA bulge			
HEK293-2-Cas9-OT97	chr4:102542567	tAtCA-tAcGCATAGAtTG-CAG	10.1			RNA bulge			
HEK293-2-Cas9-OT98	chr20:38108787	cAACACAtAGCATAG--TGCTGG	9.8			RNA bulge			

HEK293-2-Cas9-OT99	chr20:15639039	ttAttgAAAGCATAGAtTGCTGG	9.4	
HEK293-2-Cas9-OT100	chr3:48676157	GAcCcCAAAGC-TAGACTGCAGG	9.3	RNA bulge
HEK293-2-Cas9-OT101	chr3:123841181	GtgCACAAAGC-cAGACTGCAGG	9.1	RNA bulge
HEK293-2-Cas9-OT102	chr8:118064205	GtACACAAAGCATAG-C-GCTGG	8.5	RNA bulge
HEK293-2-Cas9-OT103	chr12:90645191	CAAC-CAcAaaATAGACTGCAGG	8.5	RNA bulge
HEK293-2-Cas9-OT104	chr2:213740434	ttgtACAAAGaATAGACTGtAGG	8.4	
HEK293-2-Cas9-OT105	chr17:4325456	GAACACAgAGCATAGACTGgCCTG	8.4	DNA bulge
HEK293-2-Cas9-OT106	chr5:144288349	tAACAaAAtaCATAGACTGCTAG	8.3	
HEK293-2-Cas9-OT107	chr6:55590331	GgtCACActatATAGACTG-CAG	8.2	RNA bulge
HEK293-2-Cas9-OT108	chr11:81383991	tAACACAAAGaATAaA-TGCCGG	8.1	RNA bulge
HEK293-2-Cas9-OT109	chr21:16057038	agACt-ttAGCATAGACTGCAAG	7.9	RNA bulge
HEK293-2-Cas9-OT110	chr13:33076022	acctAtgttGCATAGACTGtTGG	7.8	
HEK293-2-Cas9-OT111	chrX:65128542	cctCttAAAatATAGACTGCTGG	7.8	
HEK293-2-Cas9-OT112	chr16:25314373	GcACA-AAAGCATAaACTGCTGG	7.8	RNA bulge
HEK293-2-Cas9-OT113	chr18:34532416	GAACACttAGCATAG--TGCAGG	7.5	RNA bulge
HEK293-2-Cas9-OT114	chr15:79871119	aAACACAAACCATAGACT--GGG	7.5	RNA bulge
HEK293-2-Cas9-OT115	chr5:62646115	GAAacaAAAGCATAGACTGCCAA	7.5	
HEK293-2-Cas9-OT116	chr4:178107855	GgtgAacAAG-ATAGACTGCTGG	7.5	RNA bulge
HEK293-2-Cas9-OT117	chr3:54738106	cccCt-tcAGCATAGACTG-TGG	7.3	RNA bulge
HEK293-2-Cas9-OT118	chr13:113428467	cAAtACAAAGgATAGACTGCAGG	7.2	
HEK293-2-Cas9-OT119	chr14:44066085	atAtt-AAAa-ATAGACTGCTGG	7.0	RNA bulge
HEK293-2-Cas9-OT120	chr2:134051192	GgAtACttAttATAGACTGCTGG	7.0	
HEK293-2-Cas9-OT121	chr11:128508577	GAAttCAAAGCATAGAtTGCAGG	7.0	
HEK293-2-Cas9-OT122	chr4:91844239	GgAaACAAAGCATAGACattTGG	6.9	
HEK293-2-Cas9-OT123	chr18:56307003	GAACA--AAaCATAGACTGCAGG	6.9	RNA bulge
HEK293-2-Cas9-OT124	chr13:36067151	GgACAC-AgGCATAGACTGCTGA	6.8	RNA bulge
HEK293-2-Cas9-OT125	chr8:138631456	tctCtCccAGaATAGACTGCAGG	6.7	
HEK293-2-Cas9-OT126	chr1:108869935	GtAtAg-cAGCATAGACTGCAGG	6.6	RNA bulge
HEK293-2-Cas9-OT127	chr10:120755805	cAAaACAcAaaATAGAaTGCTGG	6.6	
HEK293-2-Cas9-OT128	chr14:85439447	GAACACAAAGCAT-tA---CTGG	6.6	RNA bulge
HEK293-2-Cas9-OT129	chr1:155830617	ctcCt-ttAGCATAtACTG-CAG	6.5	RNA bulge
HEK293-2-Cas9-OT130	chr12:40042938	GtACA-tAtt-ATAGACTGCAGG	6.4	RNA bulge
HEK293-2-Cas9-OT131	chr5:145405226	ctcCt-AccGCATAGACTGCTGG	6.4	RNA bulge
HEK293-2-Cas9-OT132	chrX:146016825	tAACACAAAGaATAaA-TG-CAG	6.3	RNA bulge
HEK293-2-Cas9-OT133	chr17:79546107	GtgCACAcAGCATAGACTGCATG	6.3	
HEK293-2-Cas9-OT134	chr18:68431104	tgtgtaAgAGCATAGACTGCTGG	6.2	
HEK293-2-Cas9-OT135	chr7:39877159	agACA-ttttCATAGACTGtCGG	6.1	RNA bulge
HEK293-2-Cas9-OT136	chr12:82740871	agACAgAtAGCATAcACTGCTGG	6.0	
HEK293-2-Cas9-OT137	chr9:290168	aAACAtAAAGaATAGACTGCAAG	5.8	
HEK293-2-Cas9-OT138	chr4:31157435	tgAttgAgtGCATAGACTGCTGG	5.8	
HEK293-2-Cas9-OT139	chr15:65377019	GAgCgatAAGCAcAGACTGCTGG	5.7	
HEK293-2-Cas9-OT140	chr6:42289364	GAACAC-ttGCATAtACTGCGAG	5.6	RNA bulge
HEK293-2-Cas9-OT141	chr18:72164409	tcAtcC-AAGCATAGACTGCAGG	5.6	RNA bulge
HEK293-2-Cas9-OT142	chr20:39714193	aAcacaAAAtCATgtACTGCTGG	5.5	
HEK293-2-Cas9-OT143	chr1:100075872	atAtt-AcAGCATAGACTGCAGG	5.4	RNA bulge
HEK293-2-Cas9-OT144	chr18:9417359	ctACAC-AAGCAcAGACTGtAGG	5.4	RNA bulge
HEK293-2-Cas9-OT145	chr1:19102356	cttgttAAAaCATAGACTGCTGG	5.3	
HEK293-2-Cas9-OT146	chr5:28446816	agAtAtAcAGCATAaACTGCTGG	5.3	
HEK293-2-Cas9-OT147	chr8:114512258	tAACAaAAtaaATAGACTGCTGG	5.3	
HEK293-2-Cas9-OT148	chr2:51329721	acACA-AcAaCATAGACT-CAGG	5.2	RNA bulge
HEK293-2-Cas9-OT149	chr11:105148271	GAAat-AAtatATAGACTG-CAG	5.2	RNA bulge
HEK293-2-Cas9-OT150	chr2:19474363	ctAttgcAAGCAcAGACTGCTGG	5.2	
HEK293-2-Cas9-OT151	chr15:91019649	atttt-tAta-ATAGACTGCTGG	5.1	RNA bulge
HEK293-2-Cas9-OT152	chr20:1860626	GAACA-AAAGCATAGA-TGCTGG	5.1	RNA bulge
HEK293-2-Cas9-OT153	chr9:8138956	ttAtA--ttGCATAaACTGCAGG	5.1	RNA bulge
HEK293-2-Cas9-OT154	chr21:45936946	tgACAg-AAGCAcAGACTG-CAG	4.9	RNA bulge
HEK293-2-Cas9-OT155	chr11:11213510	tAAaAgAAtaaATAGACTGCTGG	4.9	
HEK293-2-Cas9-OT156	chrX:35775663	GgggAacActCATAGACTGtTGG	4.9	
HEK293-2-Cas9-OT157	chr9:22700141	GAAatacAAagATAGACTcCTGG	4.9	
HEK293-2-Cas9-OT158	chr10:37873650	ttggAacActCgTAGACTGCTGG	4.9	
HEK293-2-Cas9-OT159	chr6:88067305	ctcttC-ctGCATAGACTGCAGG	4.8	RNA bulge
HEK293-2-Cas9-OT160	chr2:213040902	GAAacCAAAGCATAGAaTGCAAG	4.7	
HEK293-2-Cas9-OT161	chr18:54079243	tttCtaAcAGCATAtACTGCAGG	4.6	
HEK293-2-Cas9-OT162	chr9:79880625	tAACAaAAtaCATAGACTGCTAG	4.6	
HEK293-2-Cas9-OT163	chr1:98889291	aAACAC-AAaCATtGACTGCAGG	4.6	RNA bulge
HEK293-2-Cas9-OT164	chr5:124317028	TAACACaAAAGCATAGAC--CTGG	4.6	DNA and RNA bulge
HEK293-2-Cas9-OT165	chr4:121385010	tAACAaAAtaCATAGACTGCTAG	4.5	
HEK293-2-Cas9-OT166	chr10:72922828	agACcCtAAGCATAGACTGCAGA	4.5	
HEK293-2-Cas9-OT167	chr16:25570721	tAACAaAAtaCATAGACTGCTAG	4.3	
HEK293-2-Cas9-OT168	chr14:58397437	tgtgtatAttaATAGACTGCAGG	4.3	
HEK293-2-Cas9-OT169	chr9:76649052	GAAaACAAAG--TAGACaGCAGG	4.2	RNA bulge
HEK293-2-Cas9-OT170	chr14:69227727	GAACctActGCATAGACTGtTGG	4.2	
HEK293-2-Cas9-OT171	chr13:112155814	ctACtttAtGCATAaACTGCTGG	4.1	
HEK293-2-Cas9-OT172	chr15:41796090	agACACAcAGCAcAGACTGCAGG	4.1	
HEK293-2-Cas9-OT173	chr20:11606512	GtggA--cAGCATAGACTGCTAG	4.1	RNA bulge
HEK293-2-Cas9-OT174	chr13:38571440	tgACA-AtAGCATAGACTtCTGG	4.0	RNA bulge
HEK293-2-Cas9-OT175	chr2:118604315	tAACtCAtcatATAGACTGCTGG	4.0	
HEK293-2-Cas9-OT176	chr1:5394109	aAACACAAAGC--AGAaTGCAGG	4.0	RNA bulge
HEK293-2-Cas9-OT177	chr21:46021061	GctCACAcAGC-TAGACTGCTGG	3.8	RNA bulge
HEK293-2-Cas9-OT178	chr14:84346173	tAtagaAAAcaAagcAtaGaCGG	3.7	
HEK293-2-Cas9-OT179	chr8:33733323	GAACACAA---ATAcACTGCTGG	3.7	RNA bulge
HEK293-2-Cas9-OT180	chr20:23312078	GtgCAtAttaCATAaACTGCTGG	3.6	
HEK293-2-Cas9-OT181	chr2:181717248	GcAtt-tAAGC-TAGACTGCTGG	3.6	RNA bulge
HEK293-2-Cas9-OT182	chr3:65508027	aAgCAC-AAGCATAGgCTGCTGG	3.5	RNA bulge
HEK293-2-Cas9-OT183	chr3:36554246	ctgCA--ttGCATAGACTGCAAG	3.5	RNA bulge
HEK293-2-Cas9-OT184	chr4:125573888	GttCt-AAAGC-TAGACTGCTGG	3.5	RNA bulge
HEK293-2-Cas9-OT185	chr13:60992296	cttgttAgAGCATAaACTGCTGG	3.4	
HEK293-2-Cas9-OT186	chr13:94827431	aAtgACtAAtaATAGACTGCTGG	3.4	
HEK293-2-Cas9-OT187	chr6:139353018	aAACA--AAaCATAGACTGCTGG	3.4	RNA bulge
HEK293-2-Cas9-OT188	chr7:114884342	tAACA-AAAGCATAaACaG-CAG	3.3	RNA bulge
HEK293-2-Cas9-OT189	chr1:61647797	tgtatgttAaaAatagactgCAG	3.3	
HEK293-2-Cas9-OT190	chr2:117118678	GAtCA-AcAaaATAGACTGCTGG	3.3	RNA bulge
HEK293-2-Cas9-OT191	chr10:109038712	cAtCt--cAGtATAGACTGCAGG	3.3	RNA bulge
HEK293-2-Cas9-OT192	chr19:30799687	ccgCACAAAaCAcAGACTGCTGG	3.3	
HEK293-2-Cas9-OT193	chr12:69143345	aAACACAAAG---AGACaGCTGG	3.2	RNA bulge
HEK293-2-Cas9-OT194	chr13:91254877	GAACACAAA-CATAGtACTGAAGG	3.2	DNA and RNA bulge
HEK293-2-Cas9-OT195	chr5:119036071	tAAaAacAAagATAaACTGCTGG	3.1	
HEK293-2-Cas9-OT196	chr4:90808942	GAACACAAAG---AGcCTGCAGG	3.1	RNA bulge
HEK293-2-Cas9-OT197	chr11:87312326	ttACt--AtaCATAGACTGCTAG	3.1	RNA bulge
HEK293-2-Cas9-OT198	chr15:58545883	tgACA-AAAGCATAG-CTGCGGG	3.1	RNA bulge
HEK293-2-Cas9-OT199	chr6:98111071	acAggtAcAGCATAaACTGCTGG	3.1	

HEK293-2-Cas9-OT200	chr3:27042097	acttgttAAaCATAGACTGCTGG	3.1		
HEK293-2-Cas9-OT201	chr9:74099754	tAgCA-AAAaCATAGACTGCTGG	3.1		RNA bulge
HEK293-2-Cas9-OT202	chr10:96064388	cAgtt-AAAGaATAGACTG-CAG	3.1		RNA bulge
HEK293-2-Cas9-OT203	chr10:62393164	tttCt-AAta-ATAGACTGCTGG	3.1		RNA bulge
HEK293-2-Cas9-OT204	chr14:80958064	tcAtgttctttATAGACTGCTGG	3.0		
HEK293-2-Cas9-OT205	chr11:25852752	aAAatacAAagATAGACTGaTGG	3.0		
HEK293-2-Cas9-OT206	chr5:32790747	tAcCcCAcAGC-TAGACTGCAGG	3.0		RNA bulge
HEK293-2-Cas9-OT207	chr3:141737136	GAgCtCAAtaaATAGACTGCTGG	3.0		
HEK293-2-Cas9-OT208	chr4:107573041	GAAatCAAAGC--AGACTGCAGG	3.0		RNA bulge
HEK293-2-Cas9-OT209	chr17:32698331	tgggtg-gAaCAcAGACTG-CAG	2.9		RNA bulge
HEK293-2-Cas9-OT210	chr2:38932748	GtACc-AAAGCAcAGAaTGCAGG	2.9		RNA bulge
HEK293-2-Cas9-OT211	chr7:1973216	ctgCc--tAGCAcAGACTGCCGG	2.9		RNA bulge
HEK293-2-Cas9-OT212	chr7:87575226	atAtcCAgta-ATAGACTGCTGG	2.9		RNA bulge
HEK293-2-Cas9-OT213	chrX:37275566	actCA-tcAGCATAGACTG-TAG	2.9		RNA bulge
HEK293-2-Cas9-OT214	chr9:111564240	cgAtttcAAGCATAGACTGCTGG	2.8		
HEK293-2-Cas9-OT215	chr1:68534135	cttCtCAtta-ATAGACTGCAGG	2.8		RNA bulge
HEK293-2-Cas9-OT216	chr2:103947995	GgtCtgAcAGCATAGACTGtTGG	2.8		
HEK293-2-Cas9-OT217	chr1:217076540	ccAtc--tAGCATAGACTGCCAG	2.8		RNA bulge
HEK293-2-Cas9-OT218	chr21:28089521	GtAttC-AAGCATAGAtTGCTGG	2.8		RNA bulge
HEK293-2-Cas9-OT219	chr5:165353994	ctACAgAtAGtAatagactgCAG	2.8		
HEK293-2-Cas9-OT220	chr22:49739737	GcACA-Atta-ATAGACTGCAGG	2.8		RNA bulge
HEK293-2-Cas9-OT221	chr12:31309408	GtAtc-ttta-ATAGACTGCTGG	2.7		RNA bulge
HEK293-2-Cas9-OT222	chr3:163017581	tAACAaAAtaCATAGACTGCTAG	2.7		
HEK293-2-Cas9-OT223	chr5:146704045	attaAtttAatATAGACTGCAGG	2.7		
HEK293-2-Cas9-OT224	chr4:165467614	cAcCAaAActgATAGACTGCTAG	2.7		
HEK293-2-Cas9-OT225	chr3:61729263	aAtgt-AcAGCATAtACTGCTGG	2.7		RNA bulge
HEK293-2-Cas9-OT226	chr3:3452266	cAACAaAAtaCATAGACTGCTAG	2.7		
HEK293-2-Cas9-OT227	chr13:55441797	acttA-AAtGCATAGACTG-CAG	2.7		RNA bulge
HEK293-2-Cas9-OT228	chr7:131890171	ccctACttctaATAGACTGCAGG	2.7		
HEK293-2-Cas9-OT229	chr13:39277237	ctcaAacAcaaAgAGACTaCCGG	2.7		
HEK293-2-Cas9-OT230	chr1:179090880	GgACA--cAcCATAGACTGtAGG	2.7		RNA bulge
HEK293-2-Cas9-OT231	chr2:201632342	tAACA--gAaCATAGACTGCAGG	2.6		RNA bulge
HEK293-2-Cas9-OT232	chr12:7677401	aAAttaAtAaaATAGACTGCTAG	2.6		
HEK293-2-Cas9-OT233	chr11:19087236	tAACAaAAtaCATAGACTGCTAG	2.5		
HEK293-2-Cas9-OT234	chr8:90774485	cAAtAatAttaATAGACTGCTGG	2.5		
HEK293-2-Cas9-OT235	chr7:123941667	aAAttaAtAaaATAGACTGCTGG	2.5		
HEK293-2-Cas9-OT236	chr14:105483895	acAggCAtgagccActgTGCCCG	2.5		

Mismatched bases are in lower case. And deleted bases are indicated with dash. Inserted bases are in purple. Column is left blank when cleavage is not detected. ABE7.10 and gRNA expression vectors were cotransfected into 293T cells for validation.

Supplementary Table 18. Digenome-seq captured sites using RNF2 gRNA and Cas9

Site name	Position	DNA sequence at cleavage site	DNA cleavage score			Bulge	Deep seq primer		Validated
			Cas9	ABE7.10	BE3		FP	RP	ABE7.10
RNF2-TA	chr1:185056773	GTCATCTTAGTCATTACCTGAGG	#####	12.1	19.3		AACGGAACTCAACCATTAAGCA	CCAACATACAGAAGTCAGGAATGC	Yes
RNF2-Cas9-OT1	chr17:53928598	GTCATCTTAGTCATTA-CTGAGG	47.9	30.1	23.1	RNA bulge	TCTGGCCATTGATGCCAAAAA	ACTCTCTGAAACTGTCTGTGAAA	No
RNF2-Cas9-OT2	chr2:73160999	GagtcCgagcagAagAagaaGGG	44.7	15.2			CTCCGAGACGCAGGTGAAG	TTGCCCACCCTAGTCATTGG	No
RNF2-Cas9-OT3	chr2:177556598	GatATCTTAGcCATTACCTaGGA	22.3	6.0	4.9		CACGAGTTCATTGCTAACTCAGG	CAGAGTGGTGTCCCAAGAAGT	No
RNF2-Cas9-OT4	chr10:75832488	GcCATCTTAGTCATT-CCTGGGG	12.1	11.1	10.6	RNA bulge	GCCAGACCCGGGATTGTTT	GGCAGCAACTCTTTCACGGT	No
RNF2-Cas9-OT5	chr1:88462918	tTtActaTAGtCATTACCT-AGG	6.7			RNA bulge	GAGAGACACAACTTCAAGGCA	TCAAGCTAATTGGAACACCAG	No
RNF2-Cas9-OT6	chr6:41052122	aatATCTTAGTCATTACtgGAGC	4.1				Failed to be amplified		
RNF2-Cas9-OT7	chr3:44741014	tcCcagcactTtgggAagccAAG	3.4				Failed to be amplified		

Mismatched bases are in lower case. And deleted bases are indicated with dash. Inserted bases are in purple. Column is left blank when cleavage is not detected.

ABE7.10 and gRNA expression vectors were cotransfected into 293T cells for validation.

Supplementary Table 19. Digenome-seq captured sites using HBB-28 (T>C) gRNA and Cas9

Site name	Position	DNA sequence at cleavage site	DNA cleavage score			Bulge	Deep seq primer		Validated
			Cas9	ABE7.10	BE3		FP	RP	ABE7.10
HBB-TA	chr11:5248339	GACTTCTATGCCCAGCCCTGG	35.6		29.4		AAGAGCCAAGGACAGGTACG	ATGGTGTCTGTTTGAGGTTGC	No
HBB-Cas9-OT1	chr1:30284224	GACTTCTATaCCCAGCaCTGG	270.9		31.0		AGTAGAGTATCGCTGAGCGTG	GCCCTCTCACCTAATAGGGTTC	No
HBB-Cas9-OT2	chr22:42797028	GtCTTCcATGCCCAGCCCAGG	210.4		21.9		AAGGTCCCTGGAGCTCAGTT	GGCACTCACTTCTCTGGCAA	No
HBB-Cas9-OT3	chr5:132693664	GAgTTCTATGCCCAGCCCAAG	170.4				GCCCACTCCTGTTCTAGCTG	CTGGGTCCACTGCACACTTT	No
HBB-Cas9-OT4	chr10:79516184	GACTaCTATcCCCAGCCCTGG	169.1				ACTACCATATGACCCAGCAATC	TTGCTGTGGAATGTACCTCCTA	No
HBB-Cas9-OT5	chr22:39661111	GgCTTCTATGCCCAGCaCGGG	168.8		17.6		TGGTGATAAATGGAAAGCTATTC	GACCCTGGACTTGGGAATG	No
HBB-Cas9-OT6	chr2:179291342	GACTaCTATtCCCAGCCCTGG	146.2		35.8		GCATCTCCTACAGCTGGTGCT	TGGAAAGAGACCTGTCCCTGA	No
HBB-Cas9-OT7	chrX:133907097	aACcTCTATaCCCAGCCCAGG	141.3				TGTGGTCTCAATACCCGTTGTC	GATTCATGAGTCAGGCCAGGTT	No
HBB-Cas9-OT8	chr17:3560216	GtCTTCTATGCCCAGCaCAGG	138.9		30.2		GCAGAGCCTGGGAAAGGAAG	GAGCTGTCTGTGCTCCTCTG	No
HBB-Cas9-OT9	chr11:2712612	GgCTTCTATGCCCAGaCCCAG	136.1		21.1		GCACTTACAGTGACCACCCA	GCAAAGCCAAGCAAGCTGAA	No
HBB-Cas9-OT10	chr3:144559579	GACTaCTATtCCCAGCCCTGG	133.5	18.6	29.7		TGATGCTGCTGAAGAGCCACT	CATTTTGGGTTCCCTGACAGA	No
HBB-Cas9-OT11	chr19:47303599	GACTcCTAatCCCAGCCCTGG	121.1		40.1		TCTGCTGAGTCCACCAGTCT	CTTGGGACACAGTGGAGGTG	No
HBB-Cas9-OT12	chr4:129689565	aACTTCaATGCCCAG-CCTGG	119.6			RNA bulge	CCATCACGGTCAAGTTGGACA	TGCAGTATTGAGGGAGTTCTGC	No
HBB-Cas9-OT13	chr7:134091178	GACT-CcATGCCCAGCCtCAG	115.9		30.5	RNA bulge	ATGGCCTCACCAAAGGACTG	TTGTCAGACACACAGAGCCC	No
HBB-Cas9-OT14	chr10:130248802	GgCTTCcATGCCCAGCtCTGG	108.8				GGCTATGTGTGTGGCTCTCA	TTGCAGAGCTTCACTCCCTC	No
HBB-Cas9-OT15	chr8:10594898	aACTTCaATGCCCAGCtCCGG	101.7		4.7		TGAGACGGAGAATGTTGAGGCA	TCAAACCCTAAAAATTCGAGTTCCA	No
HBB-Cas9-OT16	chr1:76493533	GACTaCcATcCCCAGCCtTGG	96.4				ATCCCCAAGAAATATCCCTGAG	TACAGTGTGGTGCTGCTGAACA	No
HBB-Cas9-OT17	chrX:22104408	GACTaCcATaCCCAGCCCTGG	95.0				CCACTCTGATTTGGTGTCTCCT	CTGACCATCTTGGGTTCTTTGA	No
HBB-Cas9-OT18	chr1:56777557	GACcTCTATaCCCAcCCCAGG	94.9				TCTCGACAGCTCCTATGTGCC	ATGGAGGAAAACAGACATGACAAC	No
HBB-Cas9-OT19	chr4:120925289	GACTaCTATcCCCAGCCCTAG	92.5				AGGGTGCCTGAGGAAAGAAATA	CCTATAATGTGGTGCTGCTGAA	No
HBB-Cas9-OT20	chr4:82712800	GcCTTCTAcGCCCAGCCCGGG	89.4				TGGAGCGCTCTAACTAGCC	TGGAATGTTGGTCCCAGAGTT	No
HBB-Cas9-OT21	chr14:105654691	GACTTCTATaCCCAG-CCCAG	86.7			RNA bulge			
HBB-Cas9-OT22	chr2:195959361	GACTaCTATcCCCAGCCCTGG	86.4						
HBB-Cas9-OT23	chr1:165064114	GACTTtcATaCCCAGCCCTGG	79.9		11.2				
HBB-Cas9-OT24	chr12:65186652	aACTaCTATGCCCAGCCtAGG	71.6						
HBB-Cas9-OT25	chr12:5154955	GACTcCTcTGtCCAGCCCCGG	71.2						
HBB-Cas9-OT26	chr3:158785944	GACTcCcATGCtCAGCCCAGG	64.7						
HBB-Cas9-OT27	chr3:124696156	GACTaaTATaCCCAGCCtCAG	63.1		16.9				
HBB-Cas9-OT28	chr4:3720348	GAaTTCTATaCCCAGCCCTGC	54.9						
HBB-Cas9-OT29	chr1:175121605	GAC-TCTATcCCCAGaCCCGG	52.7			RNA bulge			
HBB-Cas9-OT30	chr12:123606390	aAaaggTtgaCatgGgCtGGG	50.9						
HBB-Cas9-OT31	chr21:34843987	GACTTC-AaGCCCAGCCCAGG	48.5		21.6	RNA bulge			
HBB-Cas9-OT32	chr2:30345726	GACTTCTATcCCCActCCTGG	48.2						
HBB-Cas9-OT33	chr15:90868341	tACcaCTATGCCCAGCCCCAG	46.8						
HBB-Cas9-OT34	chr10:72872815	GACTaCcATtCCCAGCCCTGG	45.1						
HBB-Cas9-OT35	chr15:70395331	GtCTTCTA-tCCCAGCCCTGG	44.6		10.1	RNA bulge			
HBB-Cas9-OT36	chr5:78234006	GACTaCcATcCCCAGCCCTGG	44.3						
HBB-Cas9-OT37	chr2:203568291	GACTTCTAga-CCAGCCCGGG	40.9		8.8	RNA bulge			
HBB-Cas9-OT38	chr1:208584950	aACcaCTATGCCCAGCCtTGG	39.5						
HBB-Cas9-OT39	chr2:157183335	atCTTCTcTGCCCAGCCCCGG	39.1						

HBB-Cas9-OT40	chr21:45039685	aACTaCTATtCCCAGCCCTGG	38.9			
HBB-Cas9-OT41	chr9:83390160	atCTTCTATtCCCAGCCCAGG	37.9		15.4	
HBB-Cas9-OT42	chrX:154212101	GACTaCcATcCCCAGCCCTGG	35.0			
HBB-Cas9-OT43	chr3:130895079	GACTcCaATGCCCAGCCCAAG	34.7			
HBB-Cas9-OT44	chr11:82125689	GACTaCcATcCCCAGCCCTGG	31.5			
HBB-Cas9-OT45	chr16:82938257	GACTaCTATGCCCA-CCCAGG	30.4			RNA bulge
HBB-Cas9-OT46	chr17:60111566	aACcaCTATGCCCAGCCtGGG	29.6			
HBB-Cas9-OT47	chr22:18969296	aACcaCTATGCCtAGCCCTGG	29.4			
HBB-Cas9-OT48	chr17:38151053	GgaTTCTATGCCCAGCaCAGG	28.6			
HBB-Cas9-OT49	chr4:145912101	GACTaCcATcCCCAGCCCTGG	27.8			
HBB-Cas9-OT50	chr1:225370907	tACTTCTAgaCCCAaCCCAGG	26.6			
HBB-Cas9-OT51	chr4:2827009	GAaTTCTATGCCaAGCCCGGG	24.4			
HBB-Cas9-OT52	chr16:17128602	GACTTCcAaaCCCAGtCCAGG	24.3			
HBB-Cas9-OT53	chr12:26205574	cAgTTCTATGCCCAGCCCCAG	23.8			
HBB-Cas9-OT54	chr11:74989539	agCTTCTATcCCCAGCCCAAG	23.4		13.1	
HBB-Cas9-OT55	chr1:58506083	GACTTCacaGCCCAaCCCTGG	23.3			
HBB-Cas9-OT56	chr6:107919277	GACTTtTAaGCCCAGCCCTGG	23.2			
HBB-Cas9-OT57	chr19:47102652	GACTaCcATcCCCAGCCCTGG	22.0			
HBB-Cas9-OT58	chr1:152888052	GACTaCcATcCCCAGCCCTGG	21.1			
HBB-Cas9-OT59	chr15:75244059	tgaTTCTATaCCCAGCCCCAG	20.4			
HBB-Cas9-OT60	chr1:78018529	GACTTCaA-GCCCAGCCtGGG	20.2			RNA bulge
HBB-Cas9-OT61	chr4:21691413	tACTTCTATGCCCA-CCCTGG	20.1			RNA bulge
HBB-Cas9-OT62	chr14:22056518	GACTaCcATcCCCAGCCCTGG	19.6			
HBB-Cas9-OT63	chr4:100539418	actacCTATGCCCAGCCCTGA	19.2	2.9	17.1	
HBB-Cas9-OT64	chr10:72512029	tggTTCTATGCCtAGCCCGGG	19.2			
HBB-Cas9-OT65	chr11:58272825	tctTTCTATGCCCAGCCCAAG	17.7		9.4	
HBB-Cas9-OT66	chr6:36580227	agCTTCTATGCCCcGCCCAGG	16.4			
HBB-Cas9-OT67	chr2:143616082	GAC-TCTATaCCCAGCCCTGA	15.8			RNA bulge
HBB-Cas9-OT68	chr19:9776223	GACTaCcATcCCCAGCCCTGG	15.6			
HBB-Cas9-OT69	chr18:31954336	GACTTCTAcaCCCAaCCCTGA	14.9			
HBB-Cas9-OT70	chr2:134331140	aggTTCTAgGCCCAGCCCAGG	14.5			
HBB-Cas9-OT71	chr11:133795303	aACTTCTcTGCCCAcCCCAGG	13.9			
HBB-Cas9-OT72	chr12:13563344	GACTaCcATtCCCAGCCCTGG	13.6			
HBB-Cas9-OT73	chr7:144066102	GACTcCTATtCC-AGCCCTGG	13.5			RNA bulge
HBB-Cas9-OT74	chr14:93582909	GACTTCTcTcCCCAGgCCCAG	11.5			
HBB-Cas9-OT75	chr1:44748955	atacTCTATaCCCAGCCCTGG	11.4		18.3	
HBB-Cas9-OT76	chrX:79586397	GACTTCagaGCCCAGCCtAGG	11.4			
HBB-Cas9-OT77	chr19:17023802	GAaTTCTATaCCCAGCCCAGC	11.2			
HBB-Cas9-OT78	chr5:33837152	GACcaCcATGCCCAGCCCTGG	10.8			
HBB-Cas9-OT79	chr3:49835285	GACTTCTAgcCCaAGCCCTGG	10.6			
HBB-Cas9-OT80	chr11:45396530	GcCTTCTgaGCCCAGCCCAGG	10.5			
HBB-Cas9-OT81	chr11:21270586	GgCcTCTATaCCCAGCCCTGC	10.4			
HBB-Cas9-OT82	chr1:19123251	agacaCTAcGCCCAGCCCCAG	9.6			

HBB-Cas9-OT83	chr22:49068356	tAaTTCTATaCCCAGCCaCGG	9.5	10.5	
HBB-Cas9-OT84	chr19:39804348	GACTcCagTGCCCAGCCCTGG	9.5		
HBB-Cas9-OT85	chr15:56792996	GACTaCcATtCCCAGCCCTGG	9.3		
HBB-Cas9-OT86	chr5:37097856	GAaTTCTATaCCCAGCCaAGG	9.1		
HBB-Cas9-OT87	chr1:192903259	attTTCTATGCCCAGCCaAGG	8.7	3.0	
HBB-Cas9-OT88	chr7:143977721	GACTcCTATt-CCAGCCCTGG	8.4	20.3	RNA bulge
HBB-Cas9-OT89	chr4:77103108	tgacTCTAgGCCCAGCCCTGG	8.3		
HBB-Cas9-OT90	chr4:8403615	GAaTTCTATGCCCcaCCCTGG	8.1		
HBB-Cas9-OT91	chr4:103026956	GACTatcATcCCCAGCCCTGG	7.7		
HBB-Cas9-OT92	chr15:88790099	GACcTCTcTGCtCAGCCCTGG	7.6		
HBB-Cas9-OT93	chr17:1807877	ctCTTCaATGCCCAGCCCTGA	7.6		
HBB-Cas9-OT94	chrX:102073393	GAC-cCTAgGCCCAGCCCAGG	6.9		RNA bulge
HBB-Cas9-OT95	chr22:46343514	GACTTCTcTGCCCAGtCCCAG	6.9		
HBB-Cas9-OT96	chr1:24385837	GACTTC-AaGCCCAGCCtGGG	6.8	3.8	RNA bulge
HBB-Cas9-OT97	chr4:155528335	GgCTTCTAgGCCCAtCCCGGG	6.7		
HBB-Cas9-OT98	chr9:33404739	GACTcCTcTgCCCAGCCCAGG	6.5		
HBB-Cas9-OT99	chrX:68511701	GgCTaCcATGCCCAGCCCGAG	6.3		
HBB-Cas9-OT100	chr12:1907706	GgCTTCTgTGCCCAGCCCTGG	6.0		
HBB-Cas9-OT101	chr1:44948901	aACTaCcATGCCCAGCCCAAG	5.9		
HBB-Cas9-OT102	chr12:100696103	GAC-TCTAaGCCCAGCCCAGG	5.8		RNA bulge
HBB-Cas9-OT103	chr13:37053223	GACTcCcATcCCCAGCCCTGG	5.6		
HBB-Cas9-OT104	chr18:7354815	GACaTCTAcaCCCAGCCtTGG	5.6		
HBB-Cas9-OT105	chr12:117232123	aACcaCTATGCCCAGCCCTGG	5.5		
HBB-Cas9-OT106	chr10:48743745	GACTaCcATcCCCAGCCCTGG	4.9		
HBB-Cas9-OT107	chr8:101456446	ataTTCTATaCCCAGCCCCTG	4.6		
HBB-Cas9-OT108	chr3:163275959	attTTCTATGCCCAGCCCCAG	4.6		
HBB-Cas9-OT109	chr20:36897837	agaTTCTATGCCCAcCCCTGG	4.5		
HBB-Cas9-OT110	chr7:64504755	aACcaCTATGCCCAGCCCTAG	4.3		
HBB-Cas9-OT111	chr3:49652324	GACcTCatTcCCCAGCCCTGG	4.3		
HBB-Cas9-OT112	chr13:112173519	ccCaTCTATGCCCAGCCCCAG	4.2		
HBB-Cas9-OT113	chr7:26831800	tgCcTCcAcaCCCAGCCCCTG	4.2		
HBB-Cas9-OT114	chr2:239535022	GAtTTCcATaCCCAGCCCAGG	4.1		
HBB-Cas9-OT115	chr5:175065327	cAtccCTATGCCCAGCCCTAG	3.6		
HBB-Cas9-OT116	chr16:54266487	GACTcCTATGCtCAGaCCAGG	3.4		
HBB-Cas9-OT117	chr13:43217548	agCTcCTATcCCCAGCCCAGG	3.4		
HBB-Cas9-OT118	chr3:182925210	cACTcCTATG-CCAGCCCTGG	3.3		RNA bulge
HBB-Cas9-OT119	chr1:77711576	aatTTCTATGCCCAGCCCAGC	3.3		
HBB-Cas9-OT120	chr1:40103178	GcCccCTATGCCCAGCCCAGG	3.1		
HBB-Cas9-OT121	chr18:50412582	tgCcTCTAaGCCCAGCCCAGG	3.0		
HBB-Cas9-OT122	chr19:17227382	cggcagcATtCCCAGCCCTGG	2.8		
HBB-Cas9-OT123	chr8:144308447	GACTTCTcTcCCCAGCCCAAG	2.7		
HBB-Cas9-OT124	chr11:66392563	tcaTataATGCCCAGCCCTCG	2.7		
HBB-Cas9-OT125	chr11:86385886	aACcTCTATGCCCAGCCaAAG	2.6		

HBB-Cas9-OT126	chr15:75041328	GACccCTAcaCCtAGCCCAGG	2.6
HBB-Cas9-OT127	chr2:57268277	GACTaCcATcCCCAGCCCTGG	2.5

Mismatched bases are in lower case. And deleted bases are indicated with dash. Inserted bases are in purple. Column is left blank when cleavage is not detected.

ABE7.10 and gRNA expression vectors were cotransfected into 293T cells for validation. Note that the length of HBB -28 (T>C) gRNA is 18-nt.

Supplementary Table 20. Digenome-seq captured sites using Dmd gRNA and Cas9

Site name	Position	DNA sequence at cleavage site	DNA cleavage score			Bulge	Deep seq primer		Validated
			Cas9	ABE7.10	BE3		FP	RP	ABE7.10
Dmd-TA	chrX:85107590	TAATAGGGGACGAACAGGGAGG	149.7	10.4			CCCTCTTGCCCCTTAACTAGG	CTGTACTTGTCTTCCAAGTGTGC	Yes
Dmd-Cas9-OT1	chr7:37695776	TtcaAGGGGACaAACAGGtTGG	143.4				AACAGAGCCCAGAGAGAGGG	GAGGACATGATTTGGGGAAGC	No
Dmd-Cas9-OT2	chr1:84184811	cAATAGaGGA-GAACAtGGGGG	126.6			RNA bulge	CAAGGTTTCACAGAATTCACACC	GAGGGGAGTGTGACCCTTAGA	No
Dmd-Cas9-OT3	chr4:64491462	gAATAGGGGACattCAGGGAGG	103.8				TGGATGCTCACTTACTTTTCAA	ATCAACTTGGCCAGAATTATTT	No
Dmd-Cas9-OT4	chr2:86793703	TAATAGGGcA-GAACAGaGGGG	100.1	0.9		RNA bulge	Failed to be amplified		
Dmd-Cas9-OT5	chr14:55699331	TAAcAGGGGAC-AACAaGGTGG	87.6		16.3	RNA bulge	CAACTTGTGAGACTCAGAACCG	GCCATTGCACTAGAATGAAAAA	No
Dmd-Cas9-OT6	chr13:89177866	gtATAGGGGAaGAgCAGGGAGG	87.2				ACACAGAAGGAATGGCTTTACC	CACAGCCCTGACCAGATGC	No
Dmd-Cas9-OT7	chr17:48683821	TAATAGGGcA-GAACAGaGGGG	87.0	1.5		RNA bulge	CAGCCATGGAAAGTACCAGAGTA	TAACCTTAGAGATGGGGGAGGC	No
Dmd-Cas9-OT8	chr11:68922531	TAAcAGGGaAC-AACAGGaGGG	81.5			RNA bulge	GCACCAGCCAACCAATGAAATA	CCCCTGTGCAATTCTTAGGTGA	No
Dmd-Cas9-OT9	chr19:54492037	TAAcAtGGGA-GAACAtGGAGG	75.2			RNA bulge	GCCAAAAGGATTGAAATACTT	TTCTCTGTGATAGCTCCAGCT	No
Dmd-Cas9-OT10	chr5:14491157(	TAtTAGaGGAC-AACAGGtAGG	71.6			RNA bulge	AGCGGATGACTGATCAACAC	GGCTTTCCCTCATGCAGTAGAT	No
Dmd-Cas9-OT11	chr2:78221140	ggATAGGGGAtgGAACAGGaAGG	71.1			DNA bulge	CACCCAACATTCGGCCACTA	AGCACCATGTGGAGTCAGTG	No
Dmd-Cas9-OT12	chr7:101075355	TAAcAGGGGA-GAACAGGaAGG	68.0			RNA bulge	CAACCAGAAGTGGACCTCACAAT	CTCTGCTCACCTGGGAATGTCAA	No
Dmd-Cas9-OT13	chrX:6320001	ctATAGGGGA-GAAaAGGGGGG	65.8			RNA bulge	ACCCTTTGAAAACCATGCTTGA	ACGATTGGAGCGAAAATGGTG	No
Dmd-Cas9-OT14	chr8:25575159	atATcctcccCttcCctGtTGG	65.5				TGTCTTGTCTATCTTCCCCAACC	GCCAAGTTGACCCCAACCAA	No
Dmd-Cas9-OT15	chr4:35623385	atATAGGGGAatAACAGGGTGG	62.4	1.1			GAGGATGGCCTTGTGCATC	CCTTTCCAAGTCTCCCCTTT	No
Dmd-Cas9-OT16	chr2:38630648	TAATA-GaGAaGAACAGGaAGG	62.0	20.6		RNA bulge	TGTGTGTTATCACGTGCAGTCTA	TGCTCAGCATAGTGCCACCT	No
Dmd-Cas9-OT17	chr18:10802193	TAAT-GGGGAaGAACAtGGTGG	60.9			RNA bulge	GGACTGCAGAAATAGCCCGT	TTTCTTGTCGGAGCACACCA	No
Dmd-Cas9-OT18	chr3:65955131	gAATcGGGGACtAACAGGtTGG	60.5				TTTAATAACTTGGACAGCAAAGA	GTCATGGCAACAGTGAGTGAAG	No
Dmd-Cas9-OT19	chr5:135081985	agtaAGGGGACtAACAGGGTGG	59.3				AAGTCCTGCCTGTGGATAACCT	CTGGGCGCCGACTATGCTA	No
Dmd-Cas9-OT20	chr5:32601862	gAATAGGGGA-GAAtAGGGAGG	58.7			RNA bulge	AATCTTAATGCAGTGAAAGCAGG	AGGAATTCTAGAAAGGGAGGGTG	No
Dmd-Cas9-OT21	chr5:24176955	TAATAtaaGA-GAACAaGGGGG	57.9			RNA bulge			
Dmd-Cas9-OT22	chr10:6731947	TtATAGGGGA-GAAaAGGGAGG	57.9			RNA bulge			
Dmd-Cas9-OT23	chr2:121399128	aAATAGGtGA-GAACAGGGAGG	55.8			RNA bulge			
Dmd-Cas9-OT24	chr2:4915336	TAATAGGtaACtAACAGGaAGG	55.4						
Dmd-Cas9-OT25	chr11:66412291	TAAcAGGGGAaGA-CAGAGAGG	54.8			RNA bulge			
Dmd-Cas9-OT26	chr17:94115973	gAATAGGGGAaGAAgAGGGGAG	54.4	2.2					
Dmd-Cas9-OT27	chr13:117094998	aAATAGGGaAgGAACAtGGAGG	54.1						
Dmd-Cas9-OT28	chr6:145370717	cAATA-GGGACaAACAGtGAGG	53.7			RNA bulge			
Dmd-Cas9-OT29	chr8:12999244	TAAagGGtGA-GAACAaGGTGG	52.8			RNA bulge			
Dmd-Cas9-OT30	chr11:40873393	TgATAGGGGAaGAAtAGGtGGG	52.3						
Dmd-Cas9-OT31	chr7:129549035	gtAatGGGGAaGAACAGGaTAG	52.2	2.0					
Dmd-Cas9-OT32	chr8:45770037	gAATAGGGtAaGAACAGGAGAG	51.9						
Dmd-Cas9-OT33	chr17:58961517	TAATAGGGG-CaAAtAGGGAGG	51.7			RNA bulge			
Dmd-Cas9-OT34	chr7:56357792	TAAcAGGGGAC-AACAGGGAGG	50.1		13.2	RNA bulge			
Dmd-Cas9-OT35	chr8:16351852	agATAGGGGAgGAACAGGaTGG	50.0						
Dmd-Cas9-OT36	chr3:93357544	TgtaAGGGGAaGAACAGGGTGG	49.9						
Dmd-Cas9-OT37	chr5:64871389	agATAGaGGACGAACAGGaTGG	47.6						

Dmd-Cas9-OT38	chr4:155671259	TtATAGGGaA-GAAaAGGGAGG	47.4			RNA bulge
Dmd-Cas9-OT39	chr4:97555378	gAATAGaGGAaGAAgAaGGAGG	46.0			
Dmd-Cas9-OT40	chr2:108235444	TAAgTAGGGGACaAAgAGGaGGG	45.9			DNA bulge
Dmd-Cas9-OT41	chr6:66482450	TAATAGaGGA-GAACAtGGGGG	45.7			RNA bulge
Dmd-Cas9-OT42	chr15:59998600	ctAagaGGGgaGAAgAGGGTGG	45.0			
Dmd-Cas9-OT43	chr10:38810754	aAATAGGaGA-GAACAtGGAGG	44.5			RNA bulge
Dmd-Cas9-OT44	chr4:105765613	gAATAaGGaACaAACAGGGGAG	44.3			
Dmd-Cas9-OT45	chr4:38324048	TAAaAGGGGA-aAACAaGGTGG	44.1			RNA bulge
Dmd-Cas9-OT46	chr2:127716262	cAATAGGGGgtGAACAGGtGGG	44.1			
Dmd-Cas9-OT47	chr10:29541213	TgAgAGGGGACaAACAGGaAGG	43.8			
Dmd-Cas9-OT48	chr3:18685277	TAAcAGGGGA-GtACAaGGTGG	43.6	18.9	10.0	RNA bulge
Dmd-Cas9-OT49	chr6:36090945	aAcaAGGGGACtAACAGGaAGG	43.2			
Dmd-Cas9-OT50	chr14:66385065	gAgTAGGGGAtGAACAGGGTAG	42.9			
Dmd-Cas9-OT51	chr17:80308914	gAAcAGGaGA-GAACAaGGAGG	42.6			RNA bulge
Dmd-Cas9-OT52	chr19:5029550	TAActGGGGACcAA-AGGGAGG	42.3			RNA bulge
Dmd-Cas9-OT53	chrX:155173850	atATAGGaGACtAACAGGaGGG	42.2			
Dmd-Cas9-OT54	chr4:12491733	tcATAGGaGAtG-ACAGGGGGG	42.0			RNA bulge
Dmd-Cas9-OT55	chr7:126692024	cctTAGGGGACaAcCAGGGTGG	41.8			
Dmd-Cas9-OT56	chr10:11721576	gggTAGGGGACG-gCAGGGAGG	41.4			RNA bulge
Dmd-Cas9-OT57	chr3:100629276	gAATAaGGGACtAgCAaGGAGG	41.1			
Dmd-Cas9-OT58	chr2:153052189	gAATAGGGtAgGAACAGGGAGG	40.8			
Dmd-Cas9-OT59	chr4:63795864	TtATAGGGGAtGAA-AGGtAGG	39.8	0.6		RNA bulge
Dmd-Cas9-OT60	chr9:64489572	aAATAGGGaACtAACAGaGGGG	39.7			
Dmd-Cas9-OT61	chr5:104284246	TgATAGaGGAC-AACAaGGAGG	39.6			RNA bulge
Dmd-Cas9-OT62	chr12:21680219	TAATAGaGGACcAcaA-GGGGG	38.7			RNA bulge
Dmd-Cas9-OT63	chr1:114713080	TcATAGGaGAtGAACAGGGTGG	38.7	1.7		
Dmd-Cas9-OT64	chr2:60965150	TgtTAGGGGA-cAACAGGaGGG	38.5			RNA bulge
Dmd-Cas9-OT65	chr16:26134066	atATAGGGcAaGAACAtGGAGG	38.4			
Dmd-Cas9-OT66	chr17:21309012	aAATA-GGGAaGAACtGGGAGG	38.3			RNA bulge
Dmd-Cas9-OT67	chr7:75655445	cAATAGGGaACaAACAGGcAGG	38.2			
Dmd-Cas9-OT68	chr1:192424561	ggtaAGGGGgCaAACAGGGAGG	38.0			
Dmd-Cas9-OT69	chr17:83291647	gAATgGGGGAaGAAgAGGGAGG	37.7			
Dmd-Cas9-OT70	chr1:181427138	agAaAGGGGACatACAGGGTGG	37.6			
Dmd-Cas9-OT71	chr2:37889907	TtATAGGGGAaaAAaAGGGTGG	37.6			
Dmd-Cas9-OT72	chr9:28811472	gtAaAGGGGAgGAACAtGGGGG	37.5	6.8		
Dmd-Cas9-OT73	chr2:92780665	gAgcAGGGGACaAACAGGaAGG	37.4			
Dmd-Cas9-OT74	chr6:22562194	gAATAGGGaAgGAACAGGaAGG	37.1			
Dmd-Cas9-OT75	chr17:45265910	TAtTAtaGGGAaGAACAcGGTGG	36.8			DNA bulge
Dmd-Cas9-OT76	chr12:77628866	TAATAGGGaACGAACgGGGGAA	36.2			
Dmd-Cas9-OT77	chr11:94452774	TAATAGGGagCtAACAGGaGAGG	35.9			DNA bulge
Dmd-Cas9-OT78	chr1:63226880	atATAaGGGAaGAACAaGtGGG	35.9			

Dmd-Cas9-OT79	chr2:103713910	TAAcTAaGGGACGAgACAGGGAGG	35.8		DNA bulge
Dmd-Cas9-OT80	chr15:56278377	TAATAaGGGA-GAACAGtGAGG	35.7	9.7	RNA bulge
Dmd-Cas9-OT81	chr5:127581091	TAccAGGGGAtGAACAGGGAAG	35.0		
Dmd-Cas9-OT82	chr9:21825768	gtAgAGGGGAC-AgCAGGGTGG	34.5		RNA bulge
Dmd-Cas9-OT83	chr15:10306363	gAATAaGGGA-GAAgAGGGTGG	34.3		RNA bulge
Dmd-Cas9-OT84	chr6:107447225	TgATAGGGGAaGAAaAGGGGGA	33.5		
Dmd-Cas9-OT85	chr4:149043784	aAATAGGGGAaaAA-AGGGGGG	33.2		RNA bulge
Dmd-Cas9-OT86	chr11:79836728	aggaAGGGGACtAACAGGtGGG	33.1		
Dmd-Cas9-OT87	chr8:109357064	ggATAGGGGAaGAACAGGcTGG	32.6	14.9	
Dmd-Cas9-OT88	chr14:12612499	TcATAGGGGA-GAACAGaGTGG	32.0		RNA bulge
Dmd-Cas9-OT89	chr13:45293667	gtgTAGGGGAgGAACAGaGGGG	31.9		
Dmd-Cas9-OT90	chr2:121124960	gggaAGGGGA-GAACAGGaAGG	31.5	0.3	RNA bulge
Dmd-Cas9-OT91	chr5:125020528	gAAaAGGGGAC-AgCAGGGGGG	31.4		RNA bulge
Dmd-Cas9-OT92	chr13:44603487	gggTAGGGGAgaAACAGGGTGG	30.0		
Dmd-Cas9-OT93	chr6:116006329	TgAcAGGGGACaGAACA-GGAGG	30.0		DNA and RNA bulge
Dmd-Cas9-OT94	chr1:134494066	TAATgtGGGACtAACAGGGGAG	29.6		
Dmd-Cas9-OT95	chr9:48036214	agAcAGGGGACtAACAGGcTGG	29.4		
Dmd-Cas9-OT96	chr11:11210080	ggAaAGGGGAcGAACAGGaAGG	29.4		
Dmd-Cas9-OT97	chr2:85548299	gtgTAGGGGAatAACAGGGCGG	29.2		
Dmd-Cas9-OT98	chr3:108258831	TAATAaaGGAtGAACAaGGGGG	29.2		
Dmd-Cas9-OT99	chr5:65779771	TgATAGGGGATGAAAT-GGGGG	28.6		RNA bulge
Dmd-Cas9-OT100	chr9:64093969	TtgcAGGGGACcAACAGGGTGG	28.1		
Dmd-Cas9-OT101	chr1:181922269	gtAatGGGGAaGAACAGGGTGG	27.6		
Dmd-Cas9-OT102	chrX:11959147	gggTAGGGGAC-AACAGGaAGG	27.4	0.3	RNA bulge
Dmd-Cas9-OT103	chr14:10953040	gtgTAGGGGAatAACAGGGTGG	27.1		
Dmd-Cas9-OT104	chr17:28739388	agAgAGGGGACaAACAGGGTGA	27.1		
Dmd-Cas9-OT105	chr7:144589742	gtgatGGGGAtGAACAGGGTGA	27.1	2.1	
Dmd-Cas9-OT106	chr9:110523475	cAATAGGaaACtAACAGGaAGG	26.6		
Dmd-Cas9-OT107	chr6:115137176	TAAT-GGGGAatAACAGGaAGG	26.3		RNA bulge
Dmd-Cas9-OT108	chr7:57387348	gcAcAGGGGACaAgCAGGGTGG	26.2		
Dmd-Cas9-OT109	chr6:38028633	TgAcAGGGGA-cAACAGGaAGG	26.1		RNA bulge
Dmd-Cas9-OT110	chr1:28001408	gtATAGGGaAaGAAgAGGGAGG	26.1	0.3	
Dmd-Cas9-OT111	chr1:72514314	gtAatatGGgatAACAtGGTGG	25.8	0.6	
Dmd-Cas9-OT112	chr16:66490565	aAATAGGGtAaGAACAaGGAGG	25.6		
Dmd-Cas9-OT113	chr12:10095589	cAtagGGGaAgGAACAGGGAAG	25.5		
Dmd-Cas9-OT114	chr12:36390660	gggTAaGGGACaAACAtGGTGG	24.7		
Dmd-Cas9-OT115	chr7:31009779	ctAatGGGagacAACAGGGTGG	24.7		
Dmd-Cas9-OT116	chr18:69242940	TAATAaGGGAaGAACAtGaGGG	24.7	1.0	
Dmd-Cas9-OT117	chr17:61427204	gAAaAGGGGAaaAACAGGGTGG	24.6		
Dmd-Cas9-OT118	chr7:6117152	TAATAaGGaA-GAACAGGaGAG	24.4		RNA bulge
Dmd-Cas9-OT119	chr18:66714998	gAATAGGGGAgaAAtAgGGGAGG	24.4		DNA bulge

Dmd-Cas9-OT120	chr12:57373467	TAATtGtGGAtGAACAGGGTGG	24.3		
Dmd-Cas9-OT121	chr13:6172287(	gAATAGGGGAgGtACAGGaGGG	24.3		
Dmd-Cas9-OT122	chr1:16199661{	TAAcAGaaGACGAACAGatGGA	24.2		
Dmd-Cas9-OT123	chr15:3099615(	TAATAGGaGAaGAAacaGGAGG	24.2		
Dmd-Cas9-OT124	chr3:14829804:	gAATAGGaGAaGAAaAGGGAGG	24.1		
Dmd-Cas9-OT125	chr10:7834335:	gggTAGGGGAaGAA-AGGGAGG	23.8		RNA bulge
Dmd-Cas9-OT126	chr13:6215046(	gAATAGGGGAgGtACAGGaGGG	23.6		
Dmd-Cas9-OT127	chr6:11065617:	aAATAGaGGAtGAgCAGGGTGG	23.5	0.5	
Dmd-Cas9-OT128	chr9:10752269(	gcATAGGGGAgaAACAGGaGGG	23.5		
Dmd-Cas9-OT129	chr1:14853030(	tgATAGGGGAC-AtCAaGGAGG	23.5		RNA bulge
Dmd-Cas9-OT130	chr6:10291921(	gAATAGaGGA-GAAaAaGGAGG	23.0		RNA bulge
Dmd-Cas9-OT131	chr5:25564921	gtAataGGGACagACAGGGAAG	22.9	1.7	
Dmd-Cas9-OT132	chr5:39429768	TAAcAGGGcACcAACAGGtTGG	22.1		
Dmd-Cas9-OT133	chr7:56755024	TAAT-GGGGACacACAGGGAGG	22.1		RNA bulge
Dmd-Cas9-OT134	chr16:5569188(	ggAgAGGGGA-GAACAGGGAGG	21.8		RNA bulge
Dmd-Cas9-OT135	chrX:16741692'	acAaAGGGGACtAACAGGaGGG	21.5		
Dmd-Cas9-OT136	chrX:99411066	gAgTAGGGaAC-AACAGGGAGG	21.5		RNA bulge
Dmd-Cas9-OT137	chr2:12324984(	gtATAGGGGACtttCAGGGTGG	21.4		
Dmd-Cas9-OT138	chr2:15885271	ccAcAGGGGAC-AACAGGaAGG	21.2		RNA bulge
Dmd-Cas9-OT139	chr3:13055441(	TAtTtGGGGAtGAACAGGtTGG	20.8		
Dmd-Cas9-OT140	chr11:1090374(	TtAgAGGGGAtGAACAGGcAGG	20.7		
Dmd-Cas9-OT141	chr11:3179408	TAAgAGGGagaGAACAaGGTGG	20.6		
Dmd-Cas9-OT142	chr11:9804412(	TtgcAGGGGAtGAA-AGGGGGG	20.6		RNA bulge
Dmd-Cas9-OT143	chr4:28727809	TAA-AGGaGAaGAAaAGGGGGG	20.5		RNA bulge
Dmd-Cas9-OT144	chr11:1183329(	gAATAGGGaAaGAACAGGcCGG	20.5		
Dmd-Cas9-OT145	chr3:38193811	TAATtGGtGA-GAACAaGGAGG	20.4		RNA bulge
Dmd-Cas9-OT146	chr3:10636564	gtgTAGGGGAatAACAGGGTGG	20.1		
Dmd-Cas9-OT147	chr19:4028071(	TtAaAGGaGA-GAACAaGGTGG	19.8		RNA bulge
Dmd-Cas9-OT148	chr11:2808120'	aAATAGaGGAC-AACAGGaGGG	19.7		RNA bulge
Dmd-Cas9-OT149	chr3:30797888	TAAaAGGGaACaAACAGGGGAG	19.6		
Dmd-Cas9-OT150	chr3:12247016(	ggggtaGGGAtGAACAGaGTGG	19.5		
Dmd-Cas9-OT151	chr5:30265308	gAAgAGGGGAaGAAaAGGGTGG	19.4		
Dmd-Cas9-OT152	chr3:20012989	aAAcAGGGGACaAAaAGGaAGG	19.4		
Dmd-Cas9-OT153	chr7:78400691	gttgAGGGGAaGAACAGGGTAG	19.4		
Dmd-Cas9-OT154	chr12:8298638(	aAATAGaGaACaAACAGGGAGG	19.3		
Dmd-Cas9-OT155	chr7:16489804	cAcTAGGGaAaGAACAGGGTGG	19.3		
Dmd-Cas9-OT156	chr16:8532596	TAAcAGaGGc-GAACAaGGGAG	19.1		RNA bulge
Dmd-Cas9-OT157	chr4:99776535	TAA-AGGGGA-GAAgAGGGGGG	19.1		RNA bulge
Dmd-Cas9-OT158	chr18:4795904'	gAATAGGaGAtaAACAGGaTGG	19.0	0.4	
Dmd-Cas9-OT159	chr14:4680900:	aAATAGGaGACaAAaAGGGAGG	19.0		
Dmd-Cas9-OT160	chr4:67132211	ccAcAGGGGACGAAtA-GGGGG	18.9		RNA bulge

Dmd-Cas9-OT161	chr6:35680582	TAAaAGGaGACaAAcAGGGGGG	18.8		
Dmd-Cas9-OT162	chr14:95962494	TAAT-tGGGAaGAACAGGaAGG	18.6		RNA bulge
Dmd-Cas9-OT163	chr1:100466403	TAtTAGGGGAaGAA-AGGaAGG	18.6		RNA bulge
Dmd-Cas9-OT164	chr9:86013502	TAAcAGGGag-GAACAtGGGGG	18.5		RNA bulge
Dmd-Cas9-OT165	chr12:11377355	gtgTAGGGGAaGAAgAGGGAGG	18.3		
Dmd-Cas9-OT166	chr3:8798085	TtATAGGaaACGAACAG-aAGG	18.3		RNA bulge
Dmd-Cas9-OT167	chr8:124242810	TtAcAaGGGAtGAA-AGGGTGG	18.1		RNA bulge
Dmd-Cas9-OT168	chr9:79927373	TAATgGaGGACGAAaAGGGAAG	18.1		
Dmd-Cas9-OT169	chrX:73669560	gtAagGGGaACaAAtAGGGTGG	17.9		
Dmd-Cas9-OT170	chrX:136634599	agATAGGGGACaAAaAaGGGGG	17.7		
Dmd-Cas9-OT171	chr9:123954243	TAAaAGGGaAaGAACA-GGAGG	17.7		RNA bulge
Dmd-Cas9-OT172	chr1:79804106	atATAGGGGAaGAAagGGGGGG	17.6		
Dmd-Cas9-OT173	chr14:14704651	ggATAGGGGACaAACAG-aTGG	17.6		RNA bulge
Dmd-Cas9-OT174	chr9:64291207	TggaAGGGGAtGAACAGGaAGG	17.6		
Dmd-Cas9-OT175	chrX:151931815	TAAcAGaGGA-GAACAaGaAGG	17.5		RNA bulge
Dmd-Cas9-OT176	chr11:68993484	atgTAGGGGA-GAAaAGGGCGG	17.4		RNA bulge
Dmd-Cas9-OT177	chr17:87718099	actTAGGGaACtAACAGGaTGG	17.0		
Dmd-Cas9-OT178	chr8:31303923	cAcTAGaGGAaGAACAGGtGGG	17.0		
Dmd-Cas9-OT179	chr18:24500095	ggggAGGGGAaGAAaAGGGAGG	16.8		
Dmd-Cas9-OT180	chr12:56294646	agATAGGGGAgGAACAaGGaTGG	16.8		DNA bulge
Dmd-Cas9-OT181	chr3:46709645	cAATA-GGGAaaAACAaGGGGG	16.8		RNA bulge
Dmd-Cas9-OT182	chr4:156206277	TAcTAaGGaACGAACtGGaGGA	16.3		
Dmd-Cas9-OT183	chr18:24862228	TtAaAGGaGACaAACA-GGTGG	16.2		RNA bulge
Dmd-Cas9-OT184	chr2:160204541	aAgcAGGGGACGAgCAGGGTGA	16.1		
Dmd-Cas9-OT185	chr4:84280645	atAcAGGGGAaGAACA-GGTGG	16.0		RNA bulge
Dmd-Cas9-OT186	chrX:161996089	gAATAGGGaAtGAAtAGGaAGG	15.9		
Dmd-Cas9-OT187	chr2:26571994	gcATAGGGGACagACAGGaAGG	15.9		
Dmd-Cas9-OT188	chr4:138865160	aAcaAGGGGACGgACAGGGGAG	15.7		
Dmd-Cas9-OT189	chr10:31255298	TAgTAGaGGGgacAACAGGaGGG	15.6		DNA bulge
Dmd-Cas9-OT190	chr9:36441999	atATAGaGGACGAACAGGaTGA	15.6		
Dmd-Cas9-OT191	chr8:54236521	TAATAGGGaAC-AACAtaGTGG	15.5		RNA bulge
Dmd-Cas9-OT192	chr11:7046487	TAATAGGGaAaGAAaAaGGGGG	15.3		
Dmd-Cas9-OT193	chr9:63897192	TAAgAGGGGACaAACA-tGGGG	15.0	1.0	RNA bulge
Dmd-Cas9-OT194	chr2:136051317	gAATAaGGGAatAACAGGGAGA	14.9		
Dmd-Cas9-OT195	chr15:60220547	gAATAGaGGAaGAAaAGGaGGG	14.9		
Dmd-Cas9-OT196	chr6:46309925	agATAGGGGACtAACAGGaTAG	14.8		
Dmd-Cas9-OT197	chr13:29685734	ccATAGGGtAaGAACAGGaAGG	14.8		
Dmd-Cas9-OT198	chr11:36798888	acAaAttaGAgGAA-AGGaAGA	14.7		RNA bulge
Dmd-Cas9-OT199	chr10:11261885	gcATAaGGGAC-AACAGGaAGG	14.7		RNA bulge
Dmd-Cas9-OT200	chr6:128435987	gAccAGGGGACaAgCAGGGCGG	14.6		
Dmd-Cas9-OT201	chr9:11299380	cAATAGGaGAaGAAgAGGGAGG	14.5		

Dmd-Cas9-OT202	chr14:12261202	gggaAGGGGACGAA-AGGGAGG	14.5	RNA bulge
Dmd-Cas9-OT203	chr14:52734955	TggTAGGGGAtG-ACAGGGAGG	14.3	RNA bulge
Dmd-Cas9-OT204	chrX:92044509	gAATAGGGGACattCAGGGAGG	14.2	
Dmd-Cas9-OT205	chr13:10064291	gAAaAGGGGACaAAtAaGGGGG	14.0	
Dmd-Cas9-OT206	chr2:19934834	cAAgAGGGGAaGAAaAGGGAGG	13.6	
Dmd-Cas9-OT207	chr6:21513454	TgAT-GGGGAaGAACtGGGAGG	13.5	RNA bulge
Dmd-Cas9-OT208	chr7:6117153	TAATAaGGaA-GAACAGGaGAG	13.3	RNA bulge
Dmd-Cas9-OT209	chr6:10460537	gAcTAGGGGACG-ACAGGaTGG	13.3	RNA bulge
Dmd-Cas9-OT210	chr13:11507506	gAATAGGGaA-GAACA-GGAGG	13.2	RNA bulge
Dmd-Cas9-OT211	chr13:62529668	gAATAGGGGAgGtACAGGaGGG	13.1	
Dmd-Cas9-OT212	chr7:18828151	cAATAGtGGACaAACAGGcAGG	12.9	
Dmd-Cas9-OT213	chr3:78350788	atATAGGGGAtGAA-AGGaAGG	12.9	RNA bulge
Dmd-Cas9-OT214	chr8:110757527	gggTtGGGGA-GAACA-GGtGG	12.9	RNA bulge
Dmd-Cas9-OT215	chr5:140220452	gtAagaGGGA-cAACAGGaGGG	12.9	RNA bulge
Dmd-Cas9-OT216	chr19:42137135	TAAgAaGGGAtGAgCAGGGAGG	12.7	
Dmd-Cas9-OT217	chr18:73285055	actTAGGGGA-GAAaAGGGTGG	12.7	RNA bulge
Dmd-Cas9-OT218	chr1:82997329	ccAaAGGGGACcAACAGGaTGG	12.4	
Dmd-Cas9-OT219	chr14:11225617	TAATAGGG-A-GAACAGGaGGG	12.3	RNA bulge
Dmd-Cas9-OT220	chr1:43753664	gctTAGGGGACGgACAaGGGGG	12.3	
Dmd-Cas9-OT221	chr13:89849608	gAAgAGGGGAgGAACAGGGAAG	12.2	
Dmd-Cas9-OT222	chr11:11168858	gcATAGGGGAaGAACAtGGCAG	11.9	
Dmd-Cas9-OT223	chr16:55518529	agAgAGGGGACcAACAGGtGGG	11.8	
Dmd-Cas9-OT224	chr6:57623865	gtgTAGGGGAC-tACAGGGCGG	11.8	RNA bulge
Dmd-Cas9-OT225	chr1:28461237	gAAaAGGGGAaGAACAaGaGAG	11.7	
Dmd-Cas9-OT226	chr1:85773000	TAAT-GGGGAgGAACtGGGTGG	11.6	RNA bulge
Dmd-Cas9-OT227	chr11:4926952	cAtTAGGGGAtGAACAGGaAAG	11.5	
Dmd-Cas9-OT228	chr3:95077709	TgATAGGGGAtGAAatGG-GGG	11.5	RNA bulge
Dmd-Cas9-OT229	chr7:101638467	gtAagGGGaACaAACAaGGGGG	11.3	
Dmd-Cas9-OT230	chr3:132326685	TggaAGGGGACG-ACAGGGTGG	11.3	RNA bulge
Dmd-Cas9-OT231	chr11:17052090	gcggAGGGGACaAgCAGGGAGG	11.3	
Dmd-Cas9-OT232	chr18:11603789	gAATAGGGGAgGAgCAatGGGG	11.2	
Dmd-Cas9-OT233	chr14:70253322	TAAaAGGGGA-GAACAGGGCGG	11.1	RNA bulge
Dmd-Cas9-OT234	chr13:62034710	gAATAGGGGAgGtACAGGaGGG	11.0	
Dmd-Cas9-OT235	chr17:81312772	TAtagaGGaAgGAACAGGaGGG	10.7	
Dmd-Cas9-OT236	chr13:11252653	TAATAGaGaAaGAA-AGGaAGG	10.6	RNA bulge
Dmd-Cas9-OT237	chr1:36383536	ggATAGGGGtaGAAaAGGGTGG	10.4	
Dmd-Cas9-OT238	chr11:87974759	cggTAGGGGA-GAAgAGGGAGG	10.3	RNA bulge
Dmd-Cas9-OT239	chr5:79139319	aAATAGGGGAaCaAACAGGaGGG	10.2	DNA bulge
Dmd-Cas9-OT240	chr2:10009094	gAgcAGGGGACcAACAGGGAAG	9.9	
Dmd-Cas9-OT241	chrX:13682027	TgAcAGGGGA-GAACAtGGAGG	9.9	RNA bulge
Dmd-Cas9-OT242	chr15:12500252	ggAT-GGGGACtAgCAGGGTGG	9.8	RNA bulge

Dmd-Cas9-OT243	chr6:114489159	agtacaGGGgaGAAgAGGGTGG	9.8		
Dmd-Cas9-OT244	chr17:74439740	agtTAGGGGAC-tACAGGGAGG	9.7		RNA bulge
Dmd-Cas9-OT245	chr19:16360259	ggAaAGGGcACGAgCAGGGAGG	9.7		
Dmd-Cas9-OT246	chr9:75812809	gAAatGGGGAaGAACtGGGGGG	9.6		
Dmd-Cas9-OT247	chr8:87468535	ctgTAGGGGAC-AAaAGGGGGG	9.6		RNA bulge
Dmd-Cas9-OT248	chr1:101210562	TccTAGGGGA-GAACgGGGAGG	9.6		RNA bulge
Dmd-Cas9-OT249	chr19:34735644	TAgaAGaGGAaGAACAGgaGGAGG	9.5		DNA bulge
Dmd-Cas9-OT250	chr4:69130284	gggTAGGGGA-GAAaAGGGGGG	9.5	0.2	RNA bulge
Dmd-Cas9-OT251	chr2:99375794	gAAaAGGGGAgGAACAaGGGAG	9.2		
Dmd-Cas9-OT252	chr14:75069974	ggggAGGGGACaAAaAGGGAGG	9.1		
Dmd-Cas9-OT253	chr3:40172361	gAAcA-GGGAaGAACAGaGTGG	9.1		RNA bulge
Dmd-Cas9-OT254	chr1:118966629	TAATAtGGGgCtAACAaGGTGG	9.0		
Dmd-Cas9-OT255	chr19:45552241	gAATgGaGGACaAACAGGtGGG	8.9		
Dmd-Cas9-OT256	chr14:78759970	ggATgGGGGAC-AACAGGGGGG	8.9		RNA bulge
Dmd-Cas9-OT257	chr6:101519955	gAtaAGGGaAgGAACAGGaAGG	8.8		
Dmd-Cas9-OT258	chr18:75592952	ggtTAGGGGACG--CAGGGAGG	8.8		RNA bulge
Dmd-Cas9-OT259	chr5:139932177	gccatGGGGACGAgCAGGGGGG	8.8		
Dmd-Cas9-OT260	chr11:10502652	gAATAGGGtAC-AAtAGGGGGG	8.5		RNA bulge
Dmd-Cas9-OT261	chr10:87254210	aAAcAGGGGAaGAA-AGGGAGG	8.5		RNA bulge
Dmd-Cas9-OT262	chr13:20644288	agAatGGGGgaGAACAGGaTGG	8.5		
Dmd-Cas9-OT263	chr14:65525159	gAAgcaGGGgacAAacaGGTGG	8.0		
Dmd-Cas9-OT264	chr1:120239864	cctTAGGGaAtGAACAGGGGAG	8.0		
Dmd-Cas9-OT265	chr4:110271828	aAAatGGGGAaGAACAGGtGGG	7.9		
Dmd-Cas9-OT266	chr17:64727434	TAAcAGaGGACGAACtaGtCGG	7.8		
Dmd-Cas9-OT267	chr15:59808971	gAtgAGGGGACcAgCAGGGAGG	7.6		
Dmd-Cas9-OT268	chr2:141173272	agggAGGGGAaGAgCAGGGTGG	7.5		
Dmd-Cas9-OT269	chr6:73680523	agAaAGGGGACaAAgAGGGAGG	7.5		
Dmd-Cas9-OT270	chr11:95542063	TAaTAGGaGA--AACAtGGAGG	7.4		RNA bulge
Dmd-Cas9-OT271	chr12:11089879	TAAgAGGaGACGAA-AGGaGGG	7.3		RNA bulge
Dmd-Cas9-OT272	chr5:142921390	cAAaAGGGGACGAACAGGcAGT	7.1		
Dmd-Cas9-OT273	chr6:111792747	gggaAGGGGA-GAAaAGGGGGG	7.1		RNA bulge
Dmd-Cas9-OT274	chr8:73402036	TAATAaGGaACtAACAaGGCAG	7.0		
Dmd-Cas9-OT275	chr18:13366123	gAATAaGtGA-GAACAGGaGGG	6.9		RNA bulge
Dmd-Cas9-OT276	chr16:10454694	TggTAGGGGAC-AACAtGGGGG	6.7		RNA bulge
Dmd-Cas9-OT277	chr4:98881222	acAaAGGGGACGAgaAGGGTGG	6.6		
Dmd-Cas9-OT278	chr18:65545097	ggtaAGGGGACaGAACAcGGAGG	6.5		DNA bulge
Dmd-Cas9-OT279	chr3:18078623	TAtTAaGGGAtGAACcAGGGTGG	6.5		DNA bulge
Dmd-Cas9-OT280	chr18:72173984	aAcTAGGaGACaAcagtGGTGG	6.5		
Dmd-Cas9-OT281	chr13:34100678	TAATctGGGA-GAACAGGaCGG	6.3		RNA bulge
Dmd-Cas9-OT282	chr15:86793131	ggggAGGGGA-aAACAGGGAGG	6.2		RNA bulge
Dmd-Cas9-OT283	chr3:140810439	gtATAGGGGACaAACgGGGAGA	6.1		

Dmd-Cas9-OT284	chrX:122095697	TAATAGGGacCaGAACAaGGGGG	6.0	DNA bulge
Dmd-Cas9-OT285	chr3:52661962	gAggAGGGGA-cAACAGGtGGG	5.6	RNA bulge
Dmd-Cas9-OT286	chr4:73601584	cAgTAaGGGAaGAAaAGGGTGG	5.6	
Dmd-Cas9-OT287	chr4:95596567	agATAGGGGACccACAgGGGTGG	5.5	DNA bulge
Dmd-Cas9-OT288	chr1:77981995	gAAaAGGaGAgGAACAGGGTGG	5.4	
Dmd-Cas9-OT289	chr1:147278999	gAATAaGGGtaGAACAtGGTGG	5.2	
Dmd-Cas9-OT290	chr4:142739830	atAatGGGGAaaAACAGGGCAG	5.1	
Dmd-Cas9-OT291	chr4:5800813	cAAcAaGaGAC-AACAGGGTGG	5.1	RNA bulge
Dmd-Cas9-OT292	chr15:247591255	TAATAGGGGAatgACAGGGCGG	4.8	
Dmd-Cas9-OT293	chr9:115181234	ggtgAGGGGA-GAACAGaGAGG	4.8	RNA bulge
Dmd-Cas9-OT294	chr4:88531939	gAATAGGGaACaAAaAtGGGGG	4.8	
Dmd-Cas9-OT295	chr9:109945754	aAgTAGGGaACaAACAGGaGGA	4.7	
Dmd-Cas9-OT296	chr3:57450883	aAATAGGaGACtAACAaGtGGG	4.6	
Dmd-Cas9-OT297	chr10:63841318	ggAgAGGGGAaGAAaAGGGAGG	4.6	
Dmd-Cas9-OT298	chr1:137898294	gtcaAGGGGAtGAAaAGGGGGG	4.5	
Dmd-Cas9-OT299	chr4:96679504	TAATAGa-GAtGAACtaGGAGG	4.2	RNA bulge
Dmd-Cas9-OT300	chr7:109271383	acAgAGGGGACtAACA-GGAGG	4.1	RNA bulge
Dmd-Cas9-OT301	chr18:77277328	gtATAGGGGA-GtACAGGGGAG	4.0	RNA bulge
Dmd-Cas9-OT302	chr13:48350102	TAATAatGGACaAA-AGGGCGG	4.0	RNA bulge
Dmd-Cas9-OT303	chr11:84358933	TAtaAaGGGAC-AAgAGGGAGG	4.0	RNA bulge
Dmd-Cas9-OT304	chr11:53073624	gAATAaGGGA-GAAgAaGGAGG	4.0	RNA bulge
Dmd-Cas9-OT305	chr5:108620489	TAATAGaGGgaGAAaAGGGAAG	3.8	
Dmd-Cas9-OT306	chr6:32722383	ccAcAGGGGA-GAACAGGtTGG	3.8	RNA bulge
Dmd-Cas9-OT307	chrX:16544107	gggTAGGGGAatGAACAGGGAG	3.5	
Dmd-Cas9-OT308	chr1:30623771	aAtaAGGGGA-GAAgAGGGAGG	3.4	RNA bulge
Dmd-Cas9-OT309	chr5:80632386	TAAgAGGaGAaGAAgAGGGTGG	3.2	
Dmd-Cas9-OT310	chr11:70027978	ggAaAGGGaACaAACAGGtGGG	3.2	
Dmd-Cas9-OT311	chr14:22367150	gggggGGGGACaAACAGGtGGG	3.1	
Dmd-Cas9-OT312	chr4:107382748	gtAaAaGGagCGAACAtaGTGG	3.0	
Dmd-Cas9-OT313	chr18:81608847	TAgTAGaGGACGAAcCAtGGAGG	3.0	DNA bulge
Dmd-Cas9-OT314	chr1:89879492	gcActaGGGgacAAgAGGGTGG	2.9	
Dmd-Cas9-OT315	chr19:26842651	gcAataGaGgaGAACAaGGGAG	2.9	
Dmd-Cas9-OT316	chr10:60043421	gggTgGGGGA-GAACAGGaGGG	2.9	RNA bulge
Dmd-Cas9-OT317	chr5:112221197	agATAGGGGAC-AACAGGGTGA	2.7	RNA bulge
Dmd-Cas9-OT318	chr1:177419743	gAtaAGGGGACaGAACAGGtAGG	2.7	DNA bulge
Dmd-Cas9-OT319	chr6:74570345	TAATAaGaGACaAACAaGtAGG	2.6	
Dmd-Cas9-OT320	chr6:55119610	atcTtaGGGAttcAtAGGGCCA	2.5	

Mismatched bases are in lower case. And deleted bases are indicated with dash. Inserted bases are in purple. Column is left blank when cleavage is not detected.
ABE7.10 and gRNA expression vectors were cotransfected into 293T cells for validation. Note that the length of Dmd gRNA is 19-nt.

Supplementary Table 21. EndoV-seq captured ABE7.10 off-target sites in untreated human and mouse genomic DNA

Species	Position	DNA sequence at cleavage site	DNA cleavage score	Region
human	None			
	chr4:33315027	ACACACACATCTGTAACTCCAGCAGTGGGAAAGAACAATTTGGCAAAGAG	8.4	SINE/B4
	chr10:17427791	ACTGTGTGTATGGTGTGTATGTGTGGTATGTGTGTGGTGTGTGTGGGG	7.5	Simple_repeat
	chr3:53674862	TGTGTATGTGGTATGTGTGTGAAATGTGTGTGTGTGATATATGAGTGTGG	3.8	Simple_repeat
	chr15:39423231	TGTTTGGCCCCTCCTTGCTCTCTCACTTCTCTCTCTCCCATAACCCGGGG	3.7	LTR/ERVK
	chr4:56234203	ACTTCTGAGGCTGTCATCTGACCTCTGTGTGTAGACACACAGAGGTCAGA	3.4	SINE/B4
	chr4:147438146	CTTTGTCAAAAATCAAGTGTCCATAAGTGTGTGGTTTTATTTCTGGGTCT	3.1	LINE/L1
	chr16:18440450	CTCACAACCATCAGTACAGCTACAGTGTACTGATGGTTTTGAGTGATCAT	2.8	SINE/B2
	chr9:57394714	ACACATACATACACACACACACAAACACACACACTTATTACACATACCCA	2.6	Simple_repeat
	chr8:125155468	GACAAATTCACATATACACATATAAACACACACACACACTGTACATCATA	2.4	Simple_repeat
	chr3:53674861	GTGTGTATGTGGTATGTGTGTGAAATGTGTGTGTGTGATATATGAGTGTG	2.3	Simple_repeat
	chr11:18138188	CTCAGTCGCATCATTTTCCTTCCTTCCTTCCTCCCTCGAGCTCAACAATT	2.0	not in repeat region
	chr3:137629440	GGGTGTGTGTGTGTGTATGTATTATTGTGTGTGTGGTGTGTGGTGTGTGT	1.9	Simple_repeat
	chr14:70782089	TCCTCCTCCCTCCTCCCTCCTCCTCCTTCCTCTTCCTCTTCTTTTGGTTT	1.6	Simple_repeat
	chr10:111533993	ATGATGATGATGATGAACGAGAAGAAGAGGAAGAAGGAGAAGAAACACAT	1.5	Simple_repeat
	chr19:42265596	CTCATAGATATACACACATACACCTACACACACAGACACACACTTATACA	1.3	not in repeat region
	chr13:30287349	TTTCCTTTCCTTTCCTTTCCTTTCCTTTCCTTTCTTTCTTTCTTTCTTTC	1.3	Simple_repeat
	chr2:28366470	TGCTTCCTGCCTAGCCATCCCCCATACCTGGTAAGGAAAGAATGGAGAGA	1.1	not in repeat region
	chr17:56534077	GTGGATAGAAGGGTATATTTGTATGTGTGTGTATATATGTAATATATGGG	1.1	Simple_repeat
	chr17:31275942	GTGGTATGGTGTGTATGTGTGGTGTGTGTGTGTGGTATGGGGTATGTGTG	1.1	Simple_repeat
	chr3:3028262	AATGTGACAACACATTGAAGCCCTGTGACACCACAGTGCAGCACTGTGAC	1.0	not in repeat region
	chr2:93155713	AGGTGAGGAAAGGTGAGGAGAGGGGAGGGGAGGAGAGGGGAGGGAGGTGG	0.9	not in repeat region
	chr9:58348528	GTAGTGTGTGTGTACGTGGTGTGTATGTGGTATGTGTGTGTAGTATGTGG	0.9	Simple_repeat
	chrX:87073696	TCTTTTCTCTTCTCTTCTCTTCTCTTCTCTTCTCTTTATTTTTTTTTTTT	0.9	Simple_repeat
	chr4:139060596	AGAACAAATCAAAACAAAAGGAAGAGAGGGAGGGAGGGAGGGAGGGAGAG	0.8	Simple_repeat
	chr2:132315277	AGTATGTATGTATGTATGTATGTATGTATGTAAGCATGTATGTATCCACC	0.7	Simple_repeat
	chr8:11449501	ATGTGGGAGGGGTGTCTGTGTGTGTTGTATATGTGTGGTATGTGTGTGTG	0.7	Simple_repeat
	chr14:40513749	TGAAAACCTAGATGTGGGAAAGGAAAGGAGAGGAGAGGAGAGGAGAGGAG	0.7	Simple_repeat
	chr17:45206464	CTATATATTTCTCTCACACACACACACACACACACACACACTCTCACA	0.7	Simple_repeat
Mouse	chr11:36628560	TCCCAAACTGTATCACTTGGGTATGTTCTTCCTTCCTTCCTTCCTTCCTT	0.6	not in repeat region
	chr6:117720862	GGGCCTGGGCCTTTACCTAACTTTGAGAGGCCTGTCGCTTCTGTCTCCAA	0.6	not in repeat region
	chr17:3023654	TGGTTTTCTACTGTGGTGTCACAAAGCTCCACTGTGGTGTCTCATGCTCC	0.5	not in repeat region
	chr18:85574689	TTTTTTTCTTTTTTTCTCCCCCCCCCTTTTTTTTGAATTTGAAAAGGCCA	0.5	not in repeat region
	chr4:117764598	TGGTGCCAGGCAGTGGTGGCACACGCCTTTAATCCCAGCACTTGGAAGGC	0.5	SINE/Alu
	chr18:23191805	ACATATTACATATACATTCATACACCACACACACACACTTATATCACAAC	0.4	SINE/B2
	chr9:52940787	TACACTTAGCATGGCCACATGGGAGGGAGGGAGGGAGGGAGGGAGGGAGG	0.3	Simple_repeat
	chr1:171058132	ATCTGACTGTACTGGGCAGTGGTGGCCACGCCCGGCCGAAACCCTGTCTT	0.3	not in repeat region
	chr4:35392482	TGGTGGTATTAGTGGAGGAGAAGGAGGAGGAGGAGGGGGAGAGGAGGAGG	0.3	LTR/ERVL-MaLR
	chr17:46188462	GGCTACACAGAGAAACCCTGTCTCGAAAAAAAATAAAAAAAAAAAAAAAT	0.3	SINE/Alu
	chr4:94619839	AAGAGAAGAGGAGAGGAGAGGAGGGGAAGAGAAGAGAAGGGAAGGGAGGG	0.3	Low_complexity
	chr4:98242010	TTTTAATTCTGCCATGGCTTGCTCTCTCTCTCTCTCTCTCCTACTTCCCC	0.3	Simple_repeat
	chr13:16299501	ATTCCTTCCTTCCTTCCTTCCTTCCTTCCTTTCCTTTCCTTTCCTTTCCT	0.2	Simple_repeat
	chr8:85705548	CTCCCCTCCTCTCCTCTCCTCCCCTTTTCCCTCCCCTTCTTTTGTGGTTC	0.2	Simple_repeat
	chr6:3141516	TGCATCCTGAGTCAAAATGGGATGTGCCCTGGGGCTAAGTGGGCCTGGCC	0.2	not in repeat region
	chr8:69901137	GGCTATATAGTAAGTCCCTATCTGCAAAAAAAAAAAAAAAAAAAAAAAAA	0.2	SINE/Alu
	chr9:115194975	AGAGAGAGAGAGAGAGAGAGAGAGAGAGAGAGAGGGAAAGAGAGTGAGAGAG	0.2	Simple_repeat
	chr10:53888441	TTTTTTTTGTTTTTTTTTTTTTTTTTTTTTTACCGCGCAGAAAGGACTGGT	0.2	Simple_repeat
	chr9:121258561	GTGGCTTCCACACAAGATGTTGACTGGGGGGGGGGGGGGGGGGGTTGATG	0.2	Low_complexity
	chr9:4313097	TTTTTTTTTTTTTTTTTTTTTTTTTTTTTTTTTGGTGCATAAGAGAGAAAAT	0.2	Simple_repeat
	chr13:90215902	TCTTCTCTTCTCTTCTTCCCTCCTCTCCCCTTCTCTCCTCTCCTCCCCTC	0.2	Simple_repeat
	chr7:15654201	ACTAAAATCCTAAGTTCCAGGGAGTCCTGGAACTTAGGATTTTAGTCTGC	0.2	LINE/L1
	chr16:80113549	GGAGAGCAGAGGGATAGAGGGAAAAGGAGAGGGAAGAGAAGAGGAGAAGA	0.2	Simple_repeat
	chr6:28967086	ATCCCCCCCTCCCCCCCCCCCCCCCCCCCCCGTATGGCTGGGCCGTGC	0.2	Simple_repeat
	chr11:89617894	GCTTTCAACAAGTGAGTTGGGAAGAAGGAGGAGGAGAAGAAGAAGAAGAA	0.2	Simple_repeat
	chr6:138608612	AATGCAGGGAGAAAGAGAGAAGAGAGAGAGAGAGAAGTTGAGAGTGAGGG	0.1	LTR/ERVK
	chr10:103830568	AGAAAGTAATTTAGATTAACCAAAGCTCTCTCTCTCTCTCTCTCTCTCTC	0.1	Simple_repeat
	chr1:188026269	AAGGGGAGAGGGAGAGGAGAAGGAAGGAGGGAGACAGGGAGACAGGGAGA	0.1	Low_complexity
	chr11:31345806	TGAAATCTAACCTCAAACCCTAACCCTAACCCTAATTCCTAACCCTAACC	0.1	not in repeat region

Supplementary Table 22. EndoV-seq captured ABE7.10 off-target sites in ABE7.10 treated human genomic DNA

Species	Position	DNA sequence at cleavage site	DNA cleavage score	Region
human	chr1:81660341	GTTCACAGGTGGGACCTGAGGAGCATTTGGGGATTTTACCACGCGATTTT	3.6	not in repeat region
	chr1:111802591	GAACTTAAAGTATAATAATAATGATAAAAAAGGGCCTTCATCCTATTCAT	2.6	LINE/L1
	chr4:9603239	ACCTCCTCTAAAGTAAACCAGTAGTAAGTTCAAAACAGCCACCCTGCAGA	1.3	LTR/ERV1
	chr5:141379653	GCTGGGACTACAGGCATACACCACCATGCTTGGCTAATTTTTAAAAATTT	1.1	SINE/Alu
	chrUn_gl000224:1018(	GATATGAATCCACGAATCCAGTGAGTACCCTAAAATAAACAATCATCCTG	0.6	LTR/ERV1
	chr19:42150226	TATAGAGCATGTACTTGGTTATTTTAAGATATAAGATTAGGTCTGACGTG	0.5	not in repeat region
	chr14:94830104	CCAATGAAGAGCGGAAAGTTGGTGCCATACACTAATGTATGGCTTATGCT	0.3	not in repeat region

Supplementary Table 23. Digenome-seq captured BE3 off-target sites in untreated human and mouse genomic DNA

Species	Position	DNA sequence at cleavage site	DNA cleavage score	Region
Human	None			
	chr2:181917323	GTCAACTGGCAAAAAGGGTTTTCTTTATATATATATATATATTCAAAAAA	80.3	Simple_repeat
	chr4:33315027	ACACACACATCTGTAACTCCAGCAGTGGGAAAGAACAATTTGGCAAAGAG	8.4	SINE/B4
	chr10:17427791	ACTGTGTGTATGGTGTGTATGTGTGGTATGTGTGTGTGGTGTGTGTGGGG	7.5	Simple_repeat
	chr3:53674862	TGTGTATGTGGTATGTGTGTGAAATGTGTGTGTGTGATATATGAGTGTGG	3.8	Simple_repeat
	chr15:39423231	TGTTTGGCCCCTCCTTGCTCTCTCACTTCTCTCTCTCCCATAACCCGGGG	3.7	LTR/ERVK
	chr4:56234203	ACTTCTGAGGCTGTCATCTGACCTCTGTGTGTAGACACACAGAGGTCAGA	3.4	SINE/B4
	chr13:100725600	GGCTCCAGATGTGGGCCGTGTGGGCTTTCCATGGAAAGCCTCTGGTACTC	2.7	LTR/ERVK
	chr12:13079447	GTATAGTGTATGTATAGTGTGTATGTGTGTGTATGACGTATATGTATGTG	2.7	Simple_repeat
	chr9:57394714	ACACATACATACACACACACACAAACACACACACTTATTACACATACCCA	2.6	Simple_repeat
	chr10:19308177	TGCATATTATTGGAGTGTGTGTGTGTGTGTGTGTGTTTGTGGTATGAAGG	2.4	Simple_repeat
	chr11:18138188	CTCAGTCGCATCATTTTCCTTCCTTCCTTCCTCCCTCGAGCTCAACAATT	2.0	not in repeat region
	chr3:137629440	GGGTGTGTGTGTGTGTATGTATTATTGTGTGTGTGGTGTGTGGTGTGTGT	1.9	Simple_repeat
	chr4:26783862	TGTGTATGCTCCTGCTTGGTGGCTCAGTCTCAACTCACTCAGATCCCAGG	1.8	LINE/L1
Mouse	chr5:116716155	CTGGGCCAGGCACAAGGGGAAGGTGTGAATTCTACTCTGACATGGTGTGA	1.7	not in repeat region
	chr14:70782089	TCCTCCTCCCTCCTCCCTCCTCCTCCTTCCTCTTCCTCTTCTTTTGGTTT	1.6	Simple_repeat
	chr10:111533993	ATGATGATGATGATGAACGAGAAGAAGAGGAAGAAGGAGAAGAAACACAT	1.5	Simple_repeat
	chr4:154691742	TTGATGAATGAATGGGTGGACAGATGGATGGATGATGAATGAATGGATGG	1.5	Simple_repeat
	chr16:6490830	TTATATATAGAGATCATAGATATACTATATATATGTGTGTATATATATGT	1.4	Simple_repeat
	chr19:42265596	CTCATAGATATACACACATACACCTACACACACAGACACACACTTATACA	1.3	not in repeat region
	chr13:30287349	TTTCCTTTCCTTTCCTTTCCTTTCCTTTCCTTTCTTTCTTTCTTTCTTTC	1.3	Simple_repeat
	chr2:28366470	TGCTTCCTGCCTAGCCATCCCCCATACCTGGTAAGGAAAGAATGGAGAGA	1.1	not in repeat region
	chrX:87073696	TCTTTTCTCTTCTCTTCTCTTCTCTTCTCTTCTCTTTATTTTTTTTTTTT	0.9	Simple_repeat
	chr3:83431197	CCCCTCCCCCTCCCCCTCCTCCTCCTCCTCCTCATCCTCTTCTTCTCCTT	0.7	Simple_repeat
	chr8:11449501	ATGTGGGAGGGGTGTCTGTGTGTGTTGTATATGTGTGGTATGTGTGTGTG	0.7	Simple_repeat
	chr17:45206464	CTATATATTTCTCTCACACACACACACACACACACACACACACTCTCACA	0.7	Simple_repeat
	chr11:36628560	TCCCAAACTGTATCACTTGGGTATGTTCTTCCTTCCTTCCTTCCTTCCTT	0.6	not in repeat region
	chr18:63173390	ACAAACACCATACACACAGATATACCACATACACTCAGACAACACAACAC	0.4	Simple_repeat

Supplementary Table 24. Digenome-seq captured BE3 off-target sites in BE3 treated human genomic DNA

Species	Position	DNA sequence at cleavage site	DNA cleavage score
Human	None		

Supplementary Table 25. Digenome-seq captured Cas9 off-target sites in untreated human and mouse genomic DNA

Species	Position	DNA sequence at cleavage site	DNA cleavage score	Region
human	None			
	chr16:3344960	AGAACCACAAAACAAAAGCAGGGTCACAGTGTACTTATATAGGTGAAAAA	13.9	not in repeat region
	chrX:103299014	ACAGAGCATCAAGTGCTCTTAAACAGTTTGACTTTTCAGCACAGGGGAGA	11.1	LINE/L1
Mouse	chr2:174046774	GGCGGGTAGTTTTGAAAGTGACAGATGAATCCACAAGGGTTTCTGTTTTA	4.0	not in repeat region
	chr11:119813144	CCCAGGTATGCCTGAAAGATAAAATTACACGTGTGTGTGTCTTTGTTTCT	3.9	not in repeat region
	chr5:93651193	AGTAGATGAAATTGTTTAAACCCCATAAGAAAAACAATATCGTCCAGCCA	2.6	LINE/L1

Supplementary Table 26. Digenome-seq captured Cas9 off-target sites in Cas9 treated human genomic DNA

Species	Position	DNA sequence at cleavage site	DNA cleavage score	Region
Human	chr21:27374159	TTAGCCAATCAAAGACAAGGATCCATCACATCTAGGCATGGCTTTGAGCT	14.7	not in repeat region
	chr2:125051757	AGGCTGGTAATTATGCTACTATCCCACATAGCATATTTATTCAAGAAATA	3.3	not in repeat region
	chr3:44742286	CCTCCCAAAGTGCTGGGATTACAGGCGTGAGCCACCGCACCCGGCCACTC	2.6	SINE/Alu

Supplementary Table 27. Summary of total off-target sites for ABE7.10, BE3, and Cas9

gRNA No.	ABE		BE		Cas9
	Score>0.1 (#)	Score>2.5 (#)	Score>0.1 (#)	Score>2.5 (#)	Score>2.5 (#)
HEK293-2	6	4	8	7	236
EMX1	34	13			
HBG	18	4	0	0	43
HBB	9	3	31	31	127
FANCF	8	2			
RNF2	5	3	9	5	7
VEGFA3	33	19	2	2	231
Dmd	80	16	18	5	320
Mean	24.1	8.0	11.3	8.3	160.7

Supplementary Table 28. Overlapping captured off-target sites between ABE7.10, BE3, and Cas9

HBG

Position	DNA sequence at cleavage site	DNA cleavage score		
		ABE7.10	BE3	Cas9
HBG1-TA	GTGGGGAAGGGGCCCCCAAGAGG	2.0		42.3
HBG2-TA	GTGGGGAAGGGGCCCCCAAGAGG	1.4		42.4
chr9:21122154	GgtGaGAAGGaGCCCaCAAGTGG	1.0		43.9
chr3:34824512	GgtaGGAAGGGGCtCCCAAGAGG	0.7		39.4
chrX:12298826	GTGGGaAAGGacCCCCaAtGAGG	0.7		54.1

VEGFA3

Position	DNA sequence at cleavage site	DNA cleavage score		
		ABE7.10	BE3	Cas9
VEGFA3-TA	GGTGAGTGAGTGTGTGCGTGTGG	18.1		214.5
chr5:89440969	aGaGAGTGAGTGTGTGCaTGAGG	41.5		175.3
chr5:29367379	tGTGAGTGAGTGTGTGtaTGGGG	20.3		49.1
chr14:65569159	aGTGAGTGAGTGTGTGtGTGGGG	15.7		111.0
chr14:62078773	tGTGAGTaAGTGTGTGtGTGTGG	11.2		56.0
chr3:193993884	aGTGAaTGAGTGTGTGtGTGTGG	10.3		72.3
chr2:230506241	GGTGAGcaAGTGTGTGtGTGTGG	9.2	9.3	
chr22:37662824	GcTGAGTGAGTGTaTGCGTGTGG	7.2		163.4
chr7:152671378	aGTGAGTGAGTGaGTGaGTGAGG	7.2		101.5
chr2:177463426	GGTGAGTGtGTGTGTGCaTGTGG	7.0		76.5
chrX:41726218	GGTGAGTGAGTGaGTGaGTGAGG	7.0		12.5
chr11:79178512	aGTGAGTGAGTGaGTGaGTGGGG	4.8		121.1
chrX:56327306	tGTGAGTGtGTGTGTGCaTGTGG	3.0		72.7
chr6:157078327	GaTGAGTGAGTGaGTGaGTGGGG	2.2		145.2
chr14:98442523	GGTGAGTGtGTGTGTGaGTGTGG	2.0		38.8
chrX:42430834	aGTGAGTGAGTGTGaGCGTGAAG	1.8		92.2
chr5:115434669	tGTGgGTGAGTGTGTGCGTGAGG	1.8		64.3
chr10:109378067	GGTGAGTGAGTGaGTGaGTGAGG	1.4		41.7
chr6:24224733	GGTGAGcGtGTGTGTGCaTGTGG	1.1		57.8
chr10:107867368	aGaGAGTGAGTGTGTGtGTtGGG	0.3		38.4
chr12:5100948	tGTGAaTGAGTGTGTGCaTGTGA	0.3		72.6

HEK293-2

Position	DNA sequence at cleavage site	DNA cleavage score		
		ABE7.10	BE3	Cas9
HEK293-2-TA	GAACACAAAGCATAGACTGCGGG	19.4	27.4	131.7
chr15:93557679	GAACACA-tGCATAGACTGCTAG	35.3	37.0	126.9
chr5:7625827	GtACACAAt-aATAGACTGCAGG	21.7	25.2	38.4
chr19:35505476	GAACAC-AAGCAcAGACTGaAGG	9.2		30.2
chr8:52596627	GAACACAtAGCATAGA-TatTGG	3.2		47.9
chr10:65717412	GAACAC-tctCATAcACTGCTGG	0.6		17.4
chr4:90522173	GAACACAAtGCATAGAtTGCCGG		33.6	82.1
chr2:19844956	aActcCAAAGCATAtACTGCTGG		28.5	65.9

chr18:22360702	GAAt-CAAAGCAcAGACTGCAGG		19.5	80.4
chr1:67142248	GAAC-CActGCATAGAaTGCAGG		8.0	21.8
chr2:192248354	GAACAC-AtaCATAGACaGCTGG		1.6	20.4

HBB

Position	DNA sequence at cleavage site	DNA cleavage score		
		ABE7.10	BE3	Cas9
HBB-TA	GACTTCTATGCCCAGCCCTGG		29.4	35.6
chr3:144559579	GACTaCTATtCCCAGCCCTGG	18.6	29.7	133.5
chrX:22104408	GACTaCcATaCCCAGCCCTGG	7.8		95.0
chr4:100539418	GACTacCTATGCCCAGCCCTGA	2.9	17.1	19.2
chr10:48743736	GACTaCcATcCCCAGCCCTGG	1.8		4.9
chr4:3720348	GAaTTCTATaCCCAGCCCTGC	1.4		54.9
chr10:72872815	GACTaCcATtCCCAGCCCTGG	1.1		45.1
chr14:22056509	GACTaCcATcCCCAGCCCTGG	0.1		19.6
chr19:47303590	GACTcCTAatCCCAGCCCTGG		40.1	121.1
chr2:179291342	GACTTCaATcCCCAGCCCTGG		35.8	146.2
chr1:30284224	GACTTCTATaCCCAGCaCTGG		31.0	270.9
chr7:134091170	GACTcC-ATGCCCAGCCtCAG		30.5	115.9
chr17:3560216	GtCTTCTATGCCCAGCaCAGG		30.2	138.9
chr22:42797028	GtCTTCcATGCCCAGCCCAGG		21.9	210.4
chr21:34843979	GACTTC-AaGCCCAGCCCAGG		21.6	48.5
chr11:2712612	GgCTTCTATGCCCAGaCCCAG		21.1	136.1
chr7:143977721	GACTcCTATt-CCAGCCCTGG		20.3	8.4
chr1:44748946	tACT-CTATaCCCAGCCCTGG		18.3	11.4
chr22:39661111	GgCTTCTATGCCCAGCaCGGG		17.6	168.8
chr3:124696144	GACTaaTATaCCCAGCCtCAG		16.9	63.1
chr9:83390160	atCTTCTATtCCCAGCCCAGG		15.4	37.9
chr11:74989539	agCTTCTATcCCCAGCCCAAG		13.1	23.4
chr1:165064106	acttTC-ATaCCCAGCCCTGG		11.2	79.9
chr22:49068356	tAaTTCTATaCCCAGCCaCGG		10.5	9.5
chr15:70395323	GtCTTCTATc-CCAGCCCTGG		10.1	44.6
chr11:58272825	tctTTCTATGCCCAGCCCAAG		9.4	17.7
chr2:203568291	GACTTCTAga-CCAGCCCGGG		8.8	40.9
chr8:10594898	aACTTCaATGCCCAGCtCCGG		4.7	101.7
chr1:24385837	GACTTC-AaGCCCAGCCtGGG		3.8	6.8
chr1:192903250	attTTCTATGCCCAGCCaAGG		3.0	8.7

RNF2

Position	DNA sequence at cleavage site	DNA cleavage score		
		ABE7.10	BE3	Cas9
RNF2-TA	GTCATCTTAGTCATTACCTGAGG	12.1	19.3	160.1
chr17:53928587	GTCATCTTAGTCATTAC-TGAGG	30.1	23.1	47.9
chr10:75832488	GcCATCTTAGTCATTcC-TGGGG	11.1	10.6	12.1
chr2:177556598	GatATCTTAGcCATTACCT-AGG	6.0	4.9	22.3
chr2:73160987	GagtcCgagcagAagAagaaGGG		15.2	44.7

Dmd				
**Position**	**DNA sequence at cleavage site**	**DNA cleavage score**		
		**ABE7.10**	**BE3**	**Cas9**
chr3:18685277	TAAcAGGGGA-GtACAaGGTGG	18.9	10.0	43.6
Dmd2-TA	TAATAGGGGACGAACAGGGAGG	10.4		149.7
chr2:38630648	TAATA-GaGAaGAACAGGaAGG	20.6		62.0
chr8:109357064	ggATAGGGGAaGAACAGGcTGG	14.9		32.6
chr15:56278377	TAATAaGGGA-GAACAGtGAGG	9.7		35.7
chr9:28811460	gtAaAGGGGAgGAACAtGGGGG	6.8		37.5
chr17:94115973	gAATAGGGGAaGAAgAGGGGAG	2.2		54.4
chr7:144589742	gtgatGGGGAtGAACAGGGTGA	2.1		27.1
chr7:129549035	gtAatGGGGAaGAACAGGaTAG	2.0		52.2
chr10:105155697	agAgAGGcagaGAcaAGaGAGA	1.8	1.0	
chr5:25564921	gtAataGGGACagACAGGGAAG	1.7		22.9
chr1:114713080	TcATAGGaGAtGAACAGGGTGG	1.7		38.7
chr17:48683821	TAATAGGGcA-GAACAGaGGGG	1.5		87.0
chr4:35623375	atATAGGGGAatAACAGGGTGG	1.1		62.4
chr9:115194975	gAgagaGaGAgagAgAGaGAGA	1.0	1.3	
chr18:69242933	TAATAaGGGAaGAACAtGaGGG	1.0		24.7
chr9:63897192	TAAgAGGGGACaAACA-tGGGG	1.0		15.0
chr2:86793703	TAATAGGGcA-GAACAGaGGGG	0.9		100.1
chr4:63795864	TtATAGGGGAtGAA-AGGtAGG	0.6		39.8
chr1:72514314	gtAatatGGgatAACAtGGTGG	0.6		25.8
chr6:110656172	aAATAGaGGAtGAgCAGGGTGG	0.5		23.5
chr18:47959047	gAATAGGaGAtaAACAGGaTGG	0.4		19.0
chr1:28001408	gtATAGGGaAaGAAgAGGGAGG	0.3		26.1
chr2:121124960	gggaAGGGGA-GAACAGGaAGG	0.3		31.5
chrX:11959147	gggTAGGGGAC-AACAGGaAGG	0.3		27.4
chr4:69130277	gggTAGGGGA-GAAaAGGGGGG	0.2		9.5
chr14:55699331	TAAcAGGGGAC-AACAaGGTGG		16.3	87.6
chr7:56357792	TAAcAGGGGAC-AACAGGGAGG		13.2	50.1

Mismatched bases are in lower case. And deleted bases are indicated with dash. Inserted bases are in purple.
Column is left blank when cleavage is not detected.

Supplementary Table 29. Multiplex EndoV-seq captured site

Position	Score
chr8:110129825	26.6
chr17:53928587	20.6
chr15:93557679	17.2
chr5:87240603	14.5
chr14:31216722	11.0
chr5:7625825	9.2
chr4:90522173	7.6
chr4:87256692	7.4
chr13:27629410	7.2
chr8:96173266	5.8
chr1:231750732	5.7
chr3:144559579	5.1
chr9:139522891	4.8
chr9:110184627	4.5
chr13:66904918	4.2
chr8:144781302	4.1
chr13:70136726	3.9
chr15:22366612	3.6
chr10:75832488	3.4
chr4:56815188	3.3
chrX:122479548	3.2
chr18:35137238	3.0
chr4:131662222	3.0
chrX:22104408	2.5
chr3:13705838	2.5
chr20:60010551	2.3
chr11:5271287	2.2
chr8:20854500	2.2
chr9:21122154	2.1
chr6:6263020	1.9
chr22:18919586	1.7
chr19:38616186	1.6
chr20:57917013	1.5
chr1:226885567	1.5
chr5:3606817	1.5
chr8:145730111	1.5
chr20:60895671	1.5
chr6:160517881	1.4
chr18:37194558	1.4
chr12:90804707	1.4
chr19:33382081	1.4
chr6:41374174	1.4
chr5:62692047	1.3
chr6:9118799	1.3
chr1:55846672	1.1
chr17:75429280	1.1
chr12:113935449	1.1
chr6:83388605	1.0
chr13:88900992	1.0
chrX:114764149	1.0
chrX:70597642	0.9
chr9:140428961	0.9
chr7:139244401	0.9
chr3:37781974	0.8
chr1:237781739	0.8
chr15:30501327	0.8
chr3:10418956	0.8
chr11:126074113	0.7
chr2:149886210	0.7
chr13:70136730	0.7
chr3:9039864	0.7
chr11:5276211	0.7
chr2:96814278	0.7
chr11:22647338	0.6
chr1:184236233	0.6
chr2:71789100	0.5
chr10:13692625	0.5
chr11:75983816	0.5
chr10:77790753	0.5
chr1:34163192	0.5
chr13:96928092	0.5
chr15:71686928	0.4
chr1:171018460	0.4
chr12:54977725	0.4
chr8:109199399	0.4
chr6:138326058	0.4
chr10:101587738	0.4
chr14:58383307	0.4
chr19:33976063	0.4
chr1:112236380	0.3
chr13:41969958	0.3
chr11:47554037	0.3
chr6:13588035	0.3
chr3:5031602	0.3
chr11:126765342	0.3
chr9:74103955	0.3
chr10:4775921	0.3
chr16:25653462	0.3
chr5:92036966	0.2
chr7:89035157	0.2
chr2:177556598	0.2
chr18:31737676	0.2
chr1:159133440	0.2
chr12:61781947	0.2
chrX:24128966	0.1
chr12:96752307	0.1
chr10:27700481	0.1
chr6:133495837	0.1
chr1:154277495	0.1
chr1:29675233	0.1
chr13:62767212	0.1
chr16:81442194	0.1
chr21:23586417	0.1

Six gRNAs was used in this multiplex EndoV-seq assay, including *HEK293-2, EMX1, FANCF, HBB-28 (T>C), RNF2* and *HBG*. And 4 on-target sites were found, which were highlighted in green.

Supplementary Table 30. Edit distance between 6 gRNA target sites

Target 1	Target 2	Edit distance
HBB	HEK2	12.0
HBB	HBG	16.0
HBB	FANCF	9.0
HBB	RNF2	13.0
HBB	EMX1	15.0
HEK2	HBG	16.0
HEK2	FANCF	13.0
HEK2	RNF2	11.0
HEK2	EMX1	11.0
HBG	FANCF	17.0
HBG	RNF2	14.0
HBG	EMX1	14.0
FANCF	RNF2	11.0
FANCF	EMX1	12.0
RNF2	EMX1	13.0
Mean edit distance		13.1

The distance between two identical sequence is 0.

Edit distance was measured by Levenshtein algorithm from Python, which shows the similarity between two strings.

The brief principle of that is aggregating the minimum edit steps (insertion=1, deletion=1 and substitution=1) required to transform one string into the other.

Supplementary Table 31. Multiplex EndoV-seq captured sites using the 6 gRNAs and ABE7.10

EMX1				
**Position**	**DNA sequence at cleavage site**	**DNA cleavage score**	**Bulge**	**Validated**
chr8:110129825	GAGTaCCaAGCAGA-GAAtAAAGG	26.6	DNA and RNA bulge	
chr14:31216722	GAGTaCcAGagAGAAGAAGAgAGG	11.0	DNA bulge	
chr4:87256692	GAGTaaGAGaAGAAGAAGAAGGG	7.4		
chr9:139522891	GtGgCCacAGCgGccGgAGgtGGC	7.1	DNA bulge	
chr8:96173266	GAGaaCCAagAGAAGAAGAAAGG	5.8		
chr1:231750732	GAGTCaGAGCAaAAGAAGtAGTG	5.7		
chr13:70136726	GgcaCtGgGCtGAAGgtagAGGA	3.9		
chr15:22366612	GAGTa-GAGCAGAgGAAGAAGGG	3.6	RNA bulge	
chrX:122479548	GgcaCtGgGCtGgAGAtGgAGGG	3.2		
chr18:35137238	GAGTatcAGCAGAAGAAaAAGGA	3.0		
chr4:131662222	GAaTCCaAG-AGAAGAAGAATGG	3.0	RNA bulge	
chr20:60010551	tgcaCtGcGgccggaggaggTGG	2.3		
chr8:20854500	GAGgCactGggGctGgAGAcGGG	2.2		
chr6:6263020	GAGT-aGAGtAGAAGAAGAgGGG	1.9	RNA bulge	
chr12:90804707	GcaTgCGgctgGgAGgtGgAGGT	1.4		
chr6:41374174	GcGggCacGCgGctGgAGgAGGG	1.4		
chr5:62692047	cAaaaaGAGCAaAAGAAGAACGG	1.3		
chr6:9118799	acGTCtGAGCAGAAGAAGAATGG	1.3		
chrX:114764149	GAGaCCagaCtGAgcAAGAgAGGG	1.0	DNA bulge	
chr9:140428961	GgGTtgGAGgAGggGgAGgAGTC	0.9		
chr7:139244401	cgactgGAGgAGggGgAtgAGGG	0.9		
chr3:37781974	GAagagGAGCAaAAGAAGAAGGG	0.8		
chr1:237781739	aAGataGAG-AGAAGAAGAgAGG	0.8	RNA bulge	
chr1:184236233	aAtaCaGAGCAGAAGAAGAATGG	0.6		
chr2:71789100	GcaggaGAGCAGAAGAAGAAAGG	0.5		
chr11:75983816	GAGTCC-AttAGAAGAAGAcAGG	0.5	RNA bulge	
chr1:34163192	GAGcaCGtGCAaggGtgaAAGTG	0.5		
chr13:96928092	GAGaCaGAG-AGAAGAAGAATGG	0.5	RNA bulge	
chr15:71686928	cAGgagGAGgAGgAGtgaAAAGG	0.4		
chr8:109199399	GAGTCaGAGCAGAAGAAagAGGA	0.4		
chr6:138326058	GgGatgGcaCtGcAGctGgAGGG	0.4		
chr10:101587738	cAtTCCagGCAGgtGtgGAgGGG	0.4		
chr14:58383307	GAaTaCcAGC-GgAGAAGAAGGG	0.4	RNA bulge	
chr1:112236380	actTatGAG-AGAAGAAGAAGAG	0.3	RNA bulge	
chr6:13588035	agcaCaGcGactgAaggtgAGGG	0.3		
chr3:5031602	GAaTCCaAGCAGgAGAAGAAGGA	0.3		
chr16:25653462	aAcaCtGAGCAGAAGAAGAATCG	0.3		
chr7:89035157	GAGTCtGAG-TGAAGAAGAATGA	0.2	RNA bulge	
chr18:31737676	GtcagaGAGCAcAAGAAGAtGGG	0.2		
chr1:159133440	tgGgagGAGagagAagAagAAAG	0.2		
chr12:61781947	tAtcagGAGCAGAAGAgaAAGGT	0.2		
chr6:133495837	GAGaagGAGaAGAAGAAGAAGGA	0.1		
chr1:154277495	GAGagaGAGAgAGAAGAAGAgGGG	0.1	DNA bulge	
chr1:29675233	GAGagaGAG-AGAAGAAGgAAGG	0.1	RNA bulge	
chr21:23586417	agcaggGAGaAGAAGAAGAAGGG	0.1		
chrX:24128966	cAaTaaagctgtttttAaAAAAG	0.1		

FANCF				
**Position**	**DNA sequence at cleavage site**	**DNA cleavage score**	**Bulge**	**Validated**
chr9:110184627	GGggcCCagaCTG-AGCACgTGA	4.5	RNA bulge	
chr4:56815188	GGcAatgCggCTGgAGgcggAGG	3.3		
chr6:160517881	GGcActgCTgCTGggGgtggTGG	1.4		
chr12:113935449	atgccCataTCTcCAGCcgCAGG	1.1		
chr13:88900992	aaAcaCaCTgCaGCtGgAggTGG	1.0		
chr11:22647338	GGAATCCCTTCTGCAGCACCTGG	0.6		
chr11:47554037	GGAATCCCTTCTaCAGCAtCCTGG	0.3	DNA bulge	
chr9:74103955	aaAggCaCTgCaGCAGgggaTGG	0.3		

HBB-28 (T>C)				
**Position**	**DNA sequence at cleavage site**	**DNA cleavage score**	**Bulge**	**Validated**
chr3:144559579	GACTaCTATtCCCAGCCCTGG	5.1		

chrX:22104408	GACTaCcATaCCCAGCCCTGG	2.5		
chr19:38616186	tACccCcAccCCCAcCCCCAG	1.6		
chr13:70136730	ttCcTCTAccttCAGCCCAGT	0.7		
chr10:27700481	cACcaCcAccCCCAaCCCAGT	0.1		
chr16:81442194	CACTTC-AgcCtCAGtgCCTGG	0.1	DNA and RNA bulge	

HBG

Position	DNA sequence at cleavage site	DNA cleavage score	Bulge	Validated
chr3:13705838	GgtGGGAtGGGGtCCCCAAGTGG	2.5		Yes (HBG-OT1)
chr1:226885567	GgtGatAAGGGGtCCCCAAGAGG	2.3		
chr11:5271287	GTGGGGAAGGGGCCCCCAAGAGG	2.2		
chr9:21122154	GgtGaGAAGGaGCCCaCAAGTGG	2.1		
chr22:18919586	GTGGtaAAGGaaCCCCaAAGGGG	1.7		
chr18:37194558	GTGGaGgcGGGGggtgCtAtAGA	1.4		
chr6:83388605	tgGaGGtgGaGGatgaCAcGAAG	1.0		
chr3:10418956	agGctccgcaGctggaggtGGGG	0.8		
chr11:5276211	GTGGGGAAGGGGCCCCCAAGAGG	0.7		
chr2:96814278	tgGaGGtgGGGGCtggaAcGAGG	0.7		
chr19:33976063	tgaGGGgAGGGaCCCtCAAGAGG	0.4		

HEK293-2

Position	DNA sequence at cleavage site	DNA cleavage score	Bulge	Validated
chr15:93557679	GAACACA-tGCATAGACTGCTAG	17.2	RNA bulge	
chr5:87240603	GAACACAAAGCATAGACTGCGGG	14.5		
chr5:7625825	GtACACAAta-ATAGACTGCAGG	9.2	RNA bulge	
chr4:90522173	GAACACAAtGCATAGAtTGCCGG	7.6		
chr13:27629410	GgcactgggGttggaggTGgGGG	7.2		
chr13:66904918	GAACACAtAGCAT--ACTGCAGG	4.2	RNA bulge	
chr8:144781302	agACACtgcagcTgGAggtgGGG	4.1		
chr5:3606817	acACAacggcaggAGgtgGCGGG	1.5		
chr8:145730111	GgcacatggGCtggGggTGgGGG	1.5		
chr20:60895671	GgcacagcAGCtggaggTGCTGG	1.5		
chr19:33382081	GggCtCtgcGgcTgGAggGgGTG	1.4		
chr1:55846672	aAAaAgAAtGgcctagaaGgGAC	1.1		
chr17:75429280	acACcacggctggAGAtgGtGGG	1.1		
chrX:70597642	GAgacactgctcTgGAggtgGTG	0.9		
chr15:30501327	GggCACtggGCtggatgTGgTGG	0.8		
chr2:149886210	GgACACtggGCtggaggTtgCGG	0.7		
chr3:9039864	GAgCcaccAcCcccagCTaCAGA	0.7		
chr10:13692625	tgACAtcAccCtggcACTGgGGC	0.5		
chr1:171018460	GccactgggGCtggGggTGgGGG	0.4		
chr13:41969958	aAcactgtgGCcggGggTGgTGG	0.3		
chr12:96752307	tAAtAaAAAGgATAcAggGgCCG	0.1		
chr13:62767212	tAAaAagAAtatattgagcaAGA	0.1		

RNF2

Position	DNA sequence at cleavage site	DNA cleavage score	Bulge	Validated
chr17:53928587	GTCATCTTAGTCATTAC-TGAGG	20.6	RNA bulge	
chr10:75832488	GcCATCTTAGTCATT-CCTGGGG	3.4	RNA bulge	
chr11:126074113	GTCAaaTTAGT-ATTACCTGGGG	0.7	RNA bulge	
chr12:54977725	cTCAcagctGaCAcTgCCTcTGG	0.4		
chr11:126765342	cagAgtgagacacTgtCtctTAA	0.3		
chr10:4775921	caCcTCTTAGTCA-TACCTGAGG	0.3	RNA bulge	
chr5:92036966	GgtATCTaAGTCATTACCTGTGG	0.2		
chr2:177556598	GatATCTTAGcCATTACCTaGGA	0.2		

Mismatched bases are in lower case. And deleted bases are indicated with dash. Inserted bases are in purple.

The on-target sites are highlighted in green. Off-target sites validated by deep sequencing is indicated with Y.

Mismatched bases are in lower case. And deleted bases are indicated with dash. Inserted bases are in purple.

Supplementary Table 32. Primers used for pUC19-SpCas9 gRNA cloning

Name	Sequence
EMX1-FP	caccGAGTCCGAGCAGAAGAAGAA
EMX1-RP	aaacTTCTTCTTCTGCTCGGACTC
FANCF-FP	caccGGAATCCCTTCTGCAGCACC
FANCF-RP	aaacGGTGCTGCAGAAGGGATTCC
HEK293-2-FP	caccGAACACAAAGCATAGACTGC
HEK293-2-RP	aaacGCAGTCTATGCTTTGTGTTC
HBG-FP	caccGTGGGGAAGGGGCCCCCAAG
HBG-RP	aaacCTTGGGGGCCCCTTCCCCAC
HBB-FP	caccGACTTCTATGCCCAGCCC
HBB-RP	aaacGGGCTGGGCATAGAAGTC
RNF2-FP	caccGTCATCTTAGTCATTACCTG
RNF2-RP	aaacCAGGTAATGACTAAGATGAC
VEGFA3-FP	caccGGTGAGTGAGTGTGTGCGTG
VEGFA3-FP	aaacCACGCACACACTCACTCACC
HEK293-1-FP	caccGGGAAAGACCCAGCATCCGT
HEK293-1-RP	aaacACGGATGCTGGGTCTTTCCC
Site 1-FP	caccGAACACAAAGCATAGACTGC
Site 1-RP	aaacGCAGTCTATGCTTTGTGTTC
Site 2-FP	caccGAGTATGAGGCATAGACTGC
Site 2-RP	aaacGCAGTCTATGCCTCATACTC
Site 3-FP	caccGTCAAGAAAGCAGAGACTGC
Site 3-RP	aaacGCAGTCTCTGCTTTCTTGAC
Site 5-FP	caccGATGAGATAATGATGAGTCA
Site 5-RP	aaacTGACTCATCATTATCTCATC
Site 6-FP	caccGGATTGACCCAGGCCAGGGC
Site 6-RP	aaacGCCCTGGCCTGGGTCAATCC
Site 14-FP	caccGGCTAAAGACCATAGACTGT
Site 14-RP	aaacACAGTCTATGGTCTTTAGCC
Dmd-FP	caccGTAATAGGGGACGAACAGGG
Dmd-RP	aaacCCCTGTTCGTCCCCTATTAC

Supplementary Table 33. Primers used for amplification of in vitro transcription template

Name	Sequence
EMX1-FP	TGTAATACGACTCACTATAGGAGTCCGAGCAGAAGAAGAAgttttagagctagaaatagc
FANCF-FP	TGTAATACGACTCACTATAGGAATCCCTTCTGCAGCACCgttttagagctagaaatagc
HEK293-2-FP	TGTAATACGACTCACTATAGGAACACAAAGCATAGACTGCgttttagagctagaaatagc
HBG-FP	TGTAATACGACTCACTATAGGTGGGGAAGGGGCCCCCAAGgttttagagctagaaatagc
HBB-28 (T>C)-FP	TGTAATACGACTCACTATAGGACTTCTATGCCCAGCCCgttttagagctagaaatagc
RNF2-FP	TGTAATACGACTCACTATAGGTCATCTTAGTCATTACCTGgttttagagctagaaatagc
Dmd-FP	TGTAATACGACTCACTATAGGTAATAGGGGACGAACAGGGgttttagagctagaaatagc
VEGFA3-FP	TGTAATACGACTCACTATAGGTGAGTGAGTGTGTGCGTGgttttagagctagaaatagc
gRNA-RP	AAAAGCACCGACTCGGTGCC

Supplementary Table 34. Primers used for amplification HEK293-2 and Dmd sites

Name	Sequence
HEK293-2-FP	GCAGGACGTCTGCCCAATA
HEK293-2-RP	GCTAGACTCAAAACCTGGCCC
Dmd-FP	ACTCACTGAAGGTGGCTTTCTG
Dmd-RP	CGGACTGGAGAAGGTAATCCTC

Supplementary Table 35. Primers used for intact gene copy number qPCR

Name	Sequence
EMX1-FP	CTCCGAGACGCAGGTGAAG
EMX1-RP	TTGCCCACCCTAGTCATTGG
FANCF-FP	CGCCGTCTCCAAGGTGAAAG
FANCF-RP	GACCAAAGCGCCGATGGA
HEK293-2-FP	ACAATGATAACAAGACCTGGCTG
HEK293-2-RP	CCCCATCTGTCAAACTGTGCG
HBG2-FP	TGGTGGGAGAAGAAAACTAGC
HBG2-RP	GTGGAGTTTAGCCAGGGACC
HBG1-FP	TCCTGGTATCCTCTATGATGGGA
HBG1-RP	GTGGAGTTTAGCCAGGGACC
HBB-FP	AAGAGCCAAGGACAGGTACG
HBB-RP	ATGGTGTCTGTTTGAGGTTGC
RNF2-FP	AACGGAACTCAACCATTAAGCA
RNF2-RP	CCAACATACAGAAGTCAGGAATGC
VEGFA3-FP	GTGCAGACGGCAGTCACTAGG
VEGFA3-RP	CTATTGGAATCCTGGAGTGACCC
GAPDH-FP	AGCTCACTGGCATGGCCTTC
GAPDH-RP	ACGCCTGCTTCACCACCTTC
Dmd-FP	CCCTCTTGCCCCTTAACTAGG
Dmd-RP	CTGTACTTGTCTTCCAAGTGTGC
Gapdh-FP	TCCCACTCTTCCACCTTCGATGC
Gapdh-RP	GGGTCTGGGATGGAAATTGTGAGG
