# Supplementary material for: Genome-wide profiling of adenine base editor specificity by EndoV-seq
Source: Nat Commun. 2019 Jan 8;10:67. doi: 10.1038/s41467-018-07988-z (PMC6325126; doi:10.1038/s41467-018-07988-z)
Supplement: Supplementary file 2 — Description of Additional Supplementary Files [file 41467_2018_7988_MOESM2_ESM.pdf]

Title: Supplementary software 1

Description: Supplementary software 1 is the software we call Site Allocator. Site Allocator can estimate the edit distance between sites captured by multiplex EndoV-seq and gRNA target sites. The 103 sites are thus divided into 6 groups assuming a site was captured by the gRNA with the smallest edit distance.
